# Allosteric activation of a cell-type-specific GPR120 inhibits amyloid pathology of Alzheimer’s disease

**DOI:** 10.1038/s43587-025-01028-4

**Published:** 2025-12-19

**Authors:** Aodi He, Yue Wang, Yuhang Shen, Zhiqiang Dong, Lingli Luo, Xiangyu Ge, Xinlu Liu, Yue Mao, Tongmei Zhang, Xinyan Li, Hao Li, Wei Jing, Ling-Qiang Zhu, Qifa Zhang, Youming Lu

**Affiliations:** 1 Innovation Center for Brain Medical Sciences of the Ministry of Education, Huazhong University of Science and Technology, Wuhan, China; 2 Department of Anatomy, School of Basic Medicine, Tongji Medical College, Huazhong University of Science and Technology, Wuhan, China; 3 Department of Pathophysiology, School of Basic Medicine, Tongji Medical College, Huazhong University of Science and Technology, Wuhan, China; 4 College of Biomedicine and Health, College of Life Science and Technology, Huazhong Agricultural University, Wuhan, China; 5 Department of Histology and Embryology, School of Basic Medicine, Tongji Medical College, Huazhong University of Science and Technology, Wuhan, China; 6 Department of Physiology, School of Basic Medicine, Tongji Medical College, Huazhong University of Science and Technology, Wuhan, China; 7 National Key Laboratory of Crop Genetic Improvement, Hubei Hongshan Laboratory, Huazhong Agricultural University, Wuhan, China

**Keywords:** Alzheimer's disease, Cognitive ageing, Ageing

## Abstract

Black rice diets are enriched with unsaturated fatty acids that are thought to be beneficial for neurodegenerative disorders in aging. Here we find that α-linolenic acid (ALA) and 11,14-eicosadienoic acid (EDA), which are naturally enriched in black rice, inhibit amyloid pathology, rescue cognition and extend lifespan in mouse preclinical models of Alzheimer’s disease via allosteric activation of G protein-coupled receptor 120 (GPR120) in plaque-associated macrophages and activated microglia. We generate the structures of GPR120 bound to ALA and EDA. We demonstrate that ALA and EDA allosterically modulate and synergistically activate GPR120 for macrophagic phagocytosis and clearance of β-amyloid aggregates in Alzheimer’s disease mice. A cell-type-specific deletion of GPR120, or Gαi1, completely abrogates the therapeutic effects of ALA and EDA. This deletion can be rescued by a constitutive active Gαi1–Q204L. These findings show a cell-type-specific function of GPR120 in the brain and provide an enriched allosteric mechanism of GPR120 activation for the treatment of Alzheimer’s disease.

## Abstract (web summary)

He, Wang, Shen, Dong and colleagues find that unsaturated fatty acids enriched in a black rice diet inhibit amyloid pathology, rescue cognition and restore physiological lifespan in mouse models of Alzheimer’s disease via allosteric activation of GPR120.

## Main

Alzheimer’s disease (AD) is most commonly one of the neurodegenerative disorders in older adults^1^. The pathological hallmarks of AD are the accumulation of extracellular amyloid-beta (Aβ) plaques and the formation of intracellular aggregated phosphorylated tau (tangles) in the brain accompanied by synaptic dysfunction and neuroinflammation^2^. Clinically, the most important manifestation of AD is progressive cognitive decline^3^. Despite great efforts towards the discoveries of an early diagnosis and the treatments for AD, only few disease-modifying therapies are currently available in clinics.

It is now recognized that chronic neuroinflammation is a major component of AD, with activated microglia and astrocytes frequently observed around Aβ plaques^4–6^. Modulating these inflammatory pathways has been recently considered as a promising therapeutic strategy^2,7^. Among factors that are able to regulate neuroinflammation, diet represents a readily accessible intervention^8,9^. For example, black rice diets (BRDs) are highly enriched with unsaturated free fatty acids that are thought to protect against neurodegeneration in aging^10^. Similarly, increased intake of ω-3 fatty acids and carotenoids from fish and plants has been linked to improved brain function and delayed cognitive decline^11^. Evidence suggests that unsaturated fatty acids, such as those from fish oil and BRDs, can act synergistically and in a dose-dependent manner to enhance working memory in cognitively healthy older adults^12,13^ and appear to be protective against the risk of AD^14,15^. Epidemiological studies on the association between diets and cognitive decline suggest a possible role of unsaturated fatty acids in maintaining normal cognitive function and possibly in preventing or delaying the onset of dementia, both of neurodegenerative and vascular origins^16,17^. However, despite these promising associations, substantial inconsistencies persist in clinical studies evaluating polyunsaturated fatty acids (PUFAs) for AD treatment^18^. Variations in study design, dosage, duration, and cohort characteristics likely contribute to these discrepancies. Thus, whether and, if yes, how dietary fatty acids confer therapeutic benefits in AD is still unknown.

In this Article, we demonstrated that administration of BRDs effectively protects against learning and memory declines in both APP/PS1 (expressing mutant human amyloid precursor protein and presenilin 1) and 5×FAD (harboring five familial mutations in APP and PSEN1) transgenic mouse models of AD^19–21^. We further identified α-linolenic acid (ALA) and 11,14-eicosadienoic acid (EDA) as two essential unsaturated fatty acids enriched in BRDs, as key mediators that reduce amyloid pathology and restore cognitive performance to wild-type levels. Along with molecular dynamic simulations and pharmacological investigations, we provided a structural mode of ALA and EDA bound to G protein-coupled receptor 120 (GPR120). Although cryo-electron microscopy (cryo-EM) structures of human GPR120 bound to different ligands including saturated 9-hydroxystearic acid, unsaturated linoleic acid and oleic acid have been reported^22^, the binding modes of ALA together with EDA remain uncharacterized. Here, we found that EDA binds to the allosteric sites of GPR120 and robustly enhances the affinity of ALA binding. This allosteric effect facilitates the interaction of GPR120 with downstream Gαi1, the inhibitory G-protein α-subunit 1. Furthermore, combination of ALA with EDA synergistically activates non-canonical Gαi1–protein kinase B (AKT) and protein kinase B–mammalian target of rapamycin complex 1 (mTORC1) signaling pathway. To establish cell-type-specific function, we generated mice with conditional knockout of GPR120, Gαi1, or mTORC1 scaffolding protein Raptor in amyloid plaque-associated macrophages and activated microglial cells (PAMAs). Loss of any of these proteins in PAMAs completely eliminates the therapeutic effects of ALA and EDA, showing a cell-type-specific effect of GPR120–Gαi1–mTORC1 activation and function. Finally, we found that activation of GPR120 in PAMAs promotes phagocytosis and clearance of Aβ aggregates and alleviates Aβ plaque burden. This study reports a cell-type-specific function of GPR120 in the brain, providing an enriched allosteric mechanism for GPR120 activation, and hence warrants insights into the development of precision targeted therapeutics for the treatment of AD.

## Results

### Enriched ALA and EDA in BRD are effective for the treatment of AD

**Extended Data Fig. 1:**
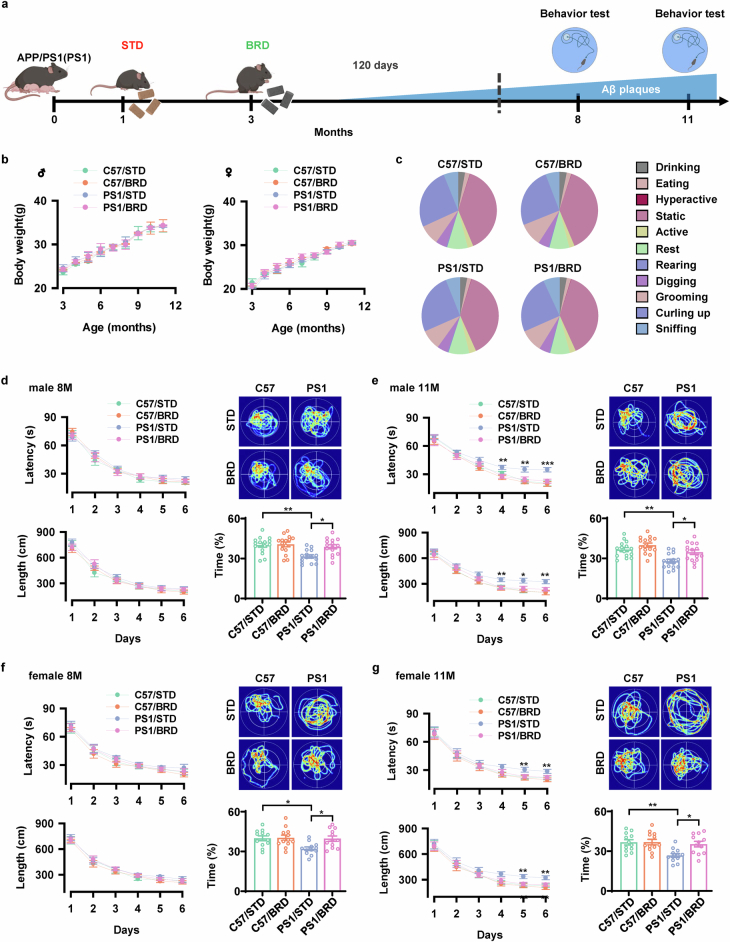
Black rice diet protects against memory declines in APP/PS1 mice. **a**, Experimental schedule for administration of BRD in APP/PS1 (PS1) mice. **b**, Body weight of PS1 and sex-matched non-AD (C57BL/6 J) control mice treated with standard (STD) or black rice diet (BRD). Data are presented as mean ± s.e.m. (for male mice *n* = 15 mice per group, for female mice *n* = 12 mice per group). **c**, Home cage activities of the individual male C57 and PS1 mice treated with STD or BRD. Data are presented as the mean time. **d, e**, The latency (left, up) and the length (left, down) of swim path to reach the hidden platform during the training sessions and the representative heat maps (right, up) and the percentage of time spent in searching of a hidden platform in targeting quadrant (quadrant 2) during the probe trial (right, down) of the individual male C57 and PS1 mice at 8 (**d**) or 11 (**e**) months (M) old of age treated with STD or BRD. Data are presented as mean ± s.e.m., *n* = 15 mice per group. **f, g**, The latency (left, up) and the length (left, down) of swim path to reach the hidden platform during the training sessions and the representative heat maps (right, up) and the percentage of time spent in searching of a hidden platform in targeting quadrant (quadrant 2) during the probe trial (right, down) of the individual female C57 and PS1 mice at 8 (**f**) or 11 (**g**) months old of age treated with STD or BRD. Data are presented as mean ± s.e.m., *n* = 12 mice per group. For **d**-**g**, two-way ANOVA with Bonferroni’s multiple comparisons test was used for analysis of latency and length, one-way ANOVA with Bonferroni’s multiple comparisons test was used for analysis of time. *P < 0.05, **P < 0.01, ***P < 0.001. The exact *P* values are reported as source data for Extended Data Fig. 1. Source data

We first investigated whether diets supplemented with black rice (BRD) exhibit therapeutic efficacy against memory decline of APP/PS1 transgenic mice. We fed 3-month-old APP/PS1 mice with a standard diet (STD) or BRD (supplemented with 50% black rice bran powder) daily for 120 consecutive days (Extended Data Fig. 1a and Supplementary Table 1). The body weight of both APP/PS1 mice and those of age- and sex-matched wild-type control mice fed on the BRD were comparable to those on STD (Extended Data Fig. 1b). Next, we evaluated the performance of the age-matched male mice by using a large battery of behavioral tests. All groups exhibited normal home cage activity (Extended Data Fig. 1c). We then tested these mice in a Morris water maze task with four trails^23,24^. During the spatial training phase, no remarkable differences in path length, latency, or floating time were observed among groups (Extended Data Fig. 1d), consistent with our previous reports that APP/PS1 mice at 8 months of age are normal in either hippocampal-dependent or hippocampal-independent learning^25,26^. However, in the hidden platform of the tests, APP/PS1 mice fed on BRD spend much more time in the target quadrant and made more platform crossings than STD-fed APP/PS1 mice (Extended Data Fig. 1d). When these mice reached the age of 11 months, the learning and memory abilities of STD-fed APP/PS1 mice were markedly reduced compared with the age-matched controls (Extended Data Fig. 1e). However, the beneficial effects of BRD on both learning and memory remained detectable (Extended Data Fig. 1e). Parallel experiments were conducted in female APP/PS1 mice. Similar protective effects were observed in female APP/PS1 mice (Extended Data Fig. 1f,g). To avoid any other potential differences between sexes, we then used male APP/PS1 mice for the following experiments in this study. BRD administration did not alter performance in the open field, in the elevated plus maze, or in novel object recognition (Supplementary Fig. 1). Together, these data show that BRD can protect against learning and memory declines in APP/PS1 mice.

**Fig. 1:**
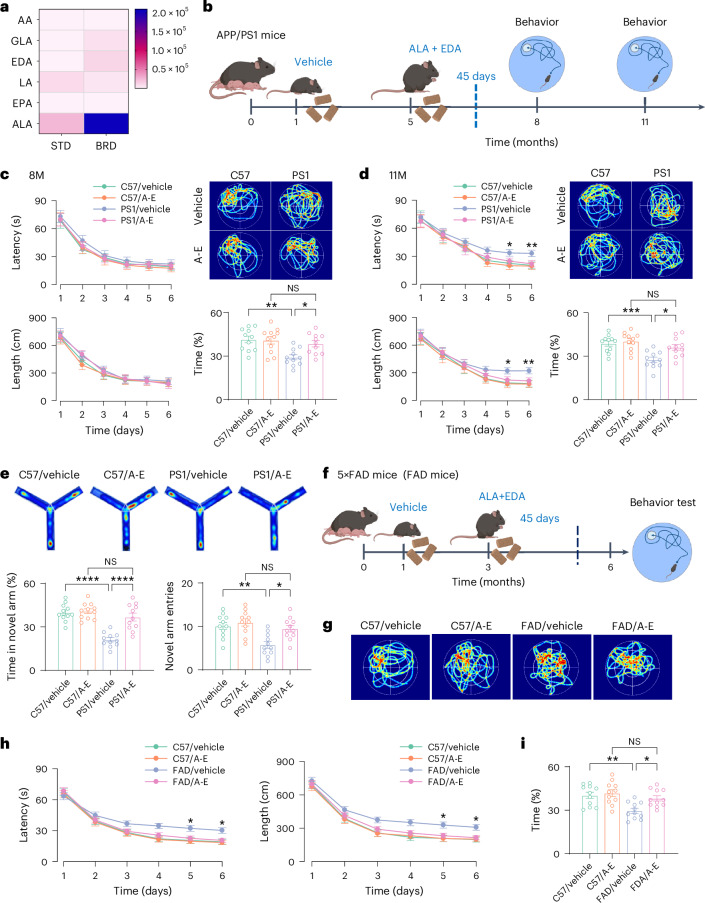
Enriched ALA and EDA in BRD rescue cognition of AD mice. **a**, Heat map of the concentrations of unsaturated free fatty acids, including arachidonic acid (AA), γ-linolenic acid (GLA), EDA, linoleic acid (LA), eicosapentaenoic acid (EPA) and ALA in STD and BRD feed. Data are presented as mean ± s.e.m., *n* = 6 per group. **b**, Experimental schedule for administration of ALA and EDA (A-E) in APP/PS1 mice. Five-month-old APP/PS1 and age-matched control mice were treated with control vehicle (saline) or A-E for 45 consecutive days. Behavior tests were conducted when the mice were 8 or 11 months old. **c**, ALA and EDA attenuate memory declines in 8-month-old AD mice (8M). The latency (left, top) and the length (left, bottom) of swim path to reach a hidden platform during the training sessions and the representative heat maps (right, top) and the percentage (right, bottom) of time spent in searching of a hidden platform in targeting quadrant (quadrant 2) during the probe trial of the individual C57BL/6J (C57) control and APP/PS1 (PS1) mice at 8 months of age treated with saline vehicle (C57/Vehicle, PS1/Vehicle) or combination with ALA and EDA (C57/A-E, PS1/A-E). **d**, ALA and EDA protect against learning and memory declines in 11-month-old AD mice (11M). The latency (left, top) and the length (left, bottom) of swim path to reach a hidden platform during the training sessions and the representative heat maps (right, top) and the percentage (right, bottom) of time spent in searching of a hidden platform in targeting quadrant (quadrant 2) during the probe trial of the individual mice at 11 months of age. **e**, ALA and EDA improve the spatial work memory of PS1 mice in the novel arm Y-maze test. Plots showing the time (left) and entries (right) in novel arm in the individual mice at 8 months of age. **f**, The experimental schedule for administration of A-E in 5×FAD mice. **g**–**i**, ALA and EDA attenuate learning and memory declines in 5×FAD mice. **g**, The representative heat maps during the probe trials for administration of A-E in 5×FAD mice. **h**, The latency and the length of swim path to reach a hidden platform during the training sessions of the individual mice at 6 months of age. **i**, The percentage of time spent in searching of a hidden platform in targeting quadrant (quadrant 2) during the probe trial of the individual mice at 6 months of age. For **c**–**i**, data are presented as mean ± s.e.m., *n* = 11 mice per group. Two-way ANOVA with Bonferroni’s multiple comparisons test was used for **c**, **d**, and **h**, and one-way ANOVA with Bonferroni’s multiple comparisons test for **e** and **i**, and analysis of time in **c** and **d**. *P < 0.05; **P < 0.01; ***P < 0.001; ****P < 0.0001; NS, no significant difference. The exact *P* values are reported as source data. Source data

BRD contains numerous nutrients including proteins, tocotrienols, anthocyanins, and free fatty acids^27^. As anthocyanins exhibit highly limited systemic bioavailability, with minimal detection of anthocyanins in rodent blood or tissues^28,29^, we hence focused on free fatty acids and determined whether and, if yes, which of them are druggable for the treatment of AD. We detected 21 free fatty acids in BRD (Fig. 1a and Supplementary Table 2), among which ALA as a representative ω-3 fatty acid and EDA as a representative ω-6 fatty acid were notably enriched compared to the STD (Fig. 1a). Based on the established health benefits of ω-3 and ω-6 fatty acids in cardiovascular and brain functions^30,31^, we hypothesized that a combination of ALA and EDA might offer therapeutic effects in AD.

**Extended Data Fig. 2:**
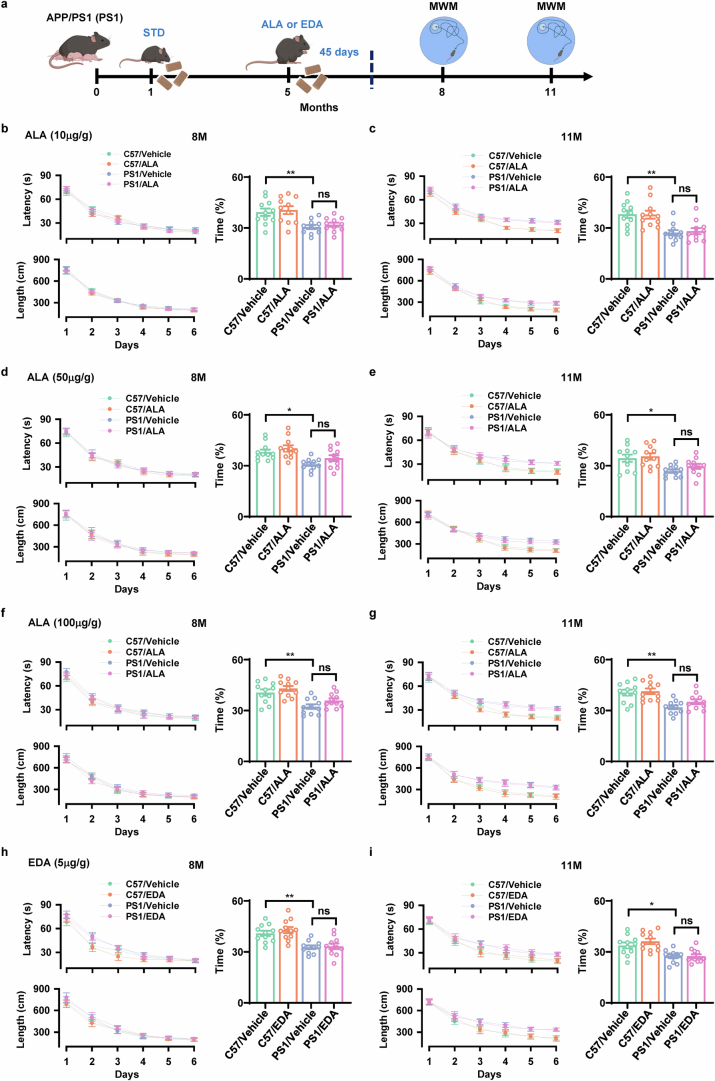
ALA or EDA alone produces no effects on behaviors of APP/PS1 mice. **a**, Experimental schedules for application of ALA or EDA for 45 consecutive days in 5-month APP/PS1 mice. The Morris water maze (MWM) task was performed when the mice were at 8 or 11 months (M) old of age. **b-g**, Application of ALA alone at 10 (**b** and **c**), 50 (**d** and **e**) or 100 (**f** and **g**) μg/day per g body weight produces no effects on learning and memory in AD mice at 8 (**b, d** and **f**) or 11 (**c, e** and **g**) months of age. The latency and the length of swim path to reach the hidden platform during the training sessions and the representative heat maps and the percentage of time spent in searching of a hidden platform in targeting quadrant (quadrant 2) during the probe trial of the individual C57 and PS1 mice treated with saline vehicle or ALA. **h, i**, Application of EDA alone at 5 μg/day per g body weight produces no effects on learning and memory in AD mice at 8 (**h**) or 11 (**i**) months of age. The latency and the length of swim path to reach the hidden platform during the training sessions and the representative heat maps and the percentage of time spent in searching of a hidden platform in targeting quadrant (quadrant 2) during the probe trial of the individual C57and PS1 mice treated with saline vehicle or EDA. For **b**-**i**, data are presented as mean ± s.e.m., *n* = 11 mice per group. Two-way ANOVA with Bonferroni’s multiple comparisons test was used for analysis of latency and length, one-way ANOVA with Bonferroni’s multiple comparisons test was used for analysis of time. *P < 0.05, **P < 0.01, and ns, no significant difference. The exact *P* values are reported as source data for Extended Data Fig. 2. Source data

To test this hypothesis, we treated AD mice (in a mouse model of AD) at 3 or 5 months of age with ALA (100, 50, or 10 μg per g body weight) or EDA (5 μg per g body weight) once daily for 45 or 120 consecutive days. Behavioral tests were then performed when the mice reached the age of 8 or 11 months (Extended Data Fig. 2a and Supplementary Fig. 2a). Our data revealed that administration of either ALA or EDA alone had no effects on spatial learning in the Morris water maze tests (Extended Data Fig. 2b–i and Supplementary Fig. 2b–i). While there was a trend suggesting potential differences during the probe trials, these did not reach statistical significance, indicating that administration of ALA or EDA alone is therapeutically ineffective against learning and memory declines in APP/PS1 mice.

A general problem with currently known fatty acids is their metabolic toxicity and off-target effects when administered at relatively high doses^32,33^. Excessive amounts of ω-6 fatty acids, or an unbalanced ω-6/ω-3 fatty acids ratio, could promote the pathogenesis of cardiovascular, cancer, inflammatory, or autoimmune diseases^32,33^. To overcome these limitations, we combined a relatively low dose of ALA (20 μg per g body weight) with EDA (2 μg per g body weight) per day for 45 consecutive days (Fig. 1b) and explored whether this combination would be therapeutically effective against memory declines in APP/PS1 mice. The combination of ALA with EDA with a ratio of 10:1 was selected as it naturally exists in BRD (Fig. 1a and Supplementary Table 1). Daily intake of either ALA- or EDA- or ALA + EDA-containing diets or calories did not differ between groups (Supplementary Fig. 3a,b). Analysis of the ALA and EDA levels in the plasma and brain tissues revealed that immediately after administration, the levels of ALA and EDA were robustly increased (Supplementary Fig. 3c–h). These results align with previous studies reporting that endogenous ALA and EDA in the brain are undetectable and administration of exogenous PUFAs can rapidly elevate their levels in brain tissues^34^. Moreover, this increase was still detectable even when the mice reached the age of 8 months (Supplementary Fig. 4).

We next assessed the impact of ALA and EDA co-administration on cognitive behavior in AD mice. Consistent with previous studies in which APP/PS1 mice at 8 months of age displayed normal spatial learning^25^, a combination of ALA and EDA had no effects on spatial learning of AD mice (Fig. 1c). However, it substantially increased the number of platform crossings and time spent in the target quadrant during the probe trials (Fig. 1c). The beneficial effects of ALA and EDA remained detectable when these mice were at 11 months of age (Fig. 1d). Furthermore, in the novel arm Y-maze test, ALA and EDA treatment improved the spatial work memory of AD mice (Fig. 1e).

More impressively, the similar therapeutic effects of ALA and EDA were observed in 5×FAD mice, another mouse model of AD (Fig. 1f–i). We conclude that a combination of ALA and EDA, but not ALA or EDA alone, is therapeutically effective for the treatment of AD mice.

### A cell-type-specific GPR120 mediates the therapeutic effects of ALA and EDA

**Fig. 2:**
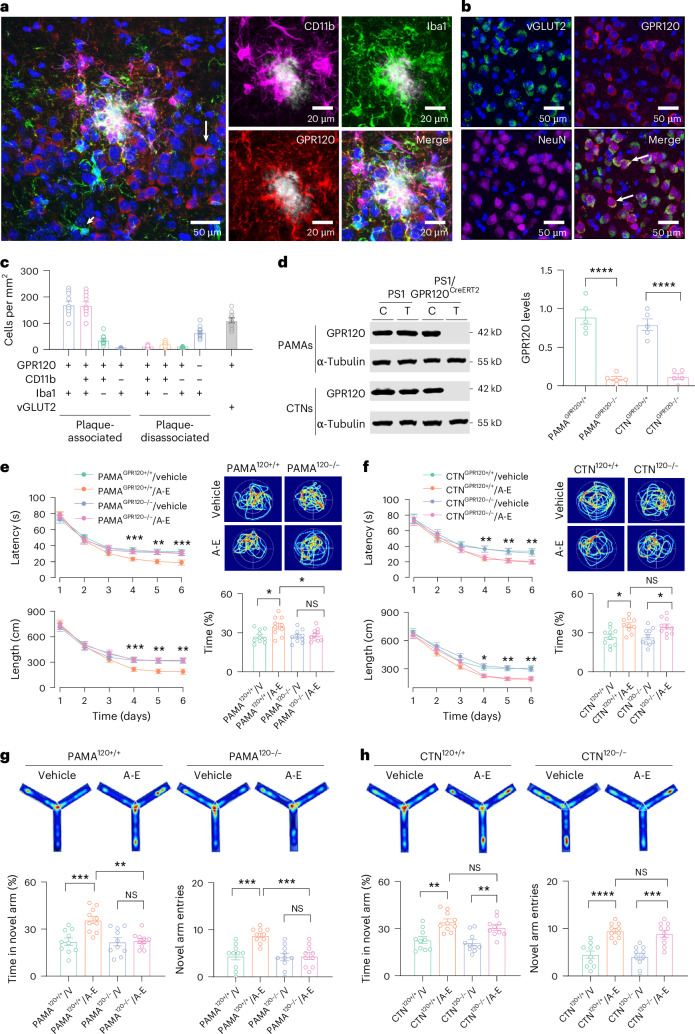
A cell-type-specific GPR120 mediates the therapeutic effects of ALA and EDA. **a**, Representative images showing the labeling of anti-CD11b (magenta), anti-Iba1 (green), and anti-GPR120 (red) with Methoxy-X04 (gray) in the cortex of APP/PS1 mice. DAPI was used for nuclear staining (blue). Plaque-surrounded CD11b^+^ cells indicate PAMAs. In areas away from plaques, CD11b^−^Iba1^−^GPR120^+^ cells (red, long arrow) and CD11b^−^Iba1^+^GPR120^−^ cells (green, short arrow) were detected. **b**, Representative images showing the labeling of vGLUT2 (green), anti-GPR120 (red), anti-NeuN (magenta), and their merged CTNs (arrows) in vGLUT2-Ai6 mice. Experiments were repeated at least three times independently with similar results. **c**, A plot showing the density (number of cells per mm^2^) of GPR120-expressing (GPR120^+^) and CD11b- and Iba1-labeled cells surrounding the amyloid plaques (within 30 μm of X04^+^ plaques, plaque-associated) or located away from plaques (>30 μm range of X04^+^ plaques, plaque-disassociated) in the cortex of APP/PS1 mice at 5 months of age, as well as the numbers of GPR120-expressing neurons (GPR120^+^vGLUT2^+^) in vGLUT2-Ai6 mice. Different cell types are represented by different color blocks, as indicated by the *x*-axis labels. Data are presented as mean ± s.e.m., *n* = 10 mice. **d**, Genetic deletion of GPR120 in PAMAs or CTNs. Representative blots and a plot showing GPR120 in PAMAs and CTNs from APP/PS1 and PS1/GPR120^CreERT2^ mice treated with control vehicle (C) or tamoxifen (T). Data are presented as mean ± s.e.m., *n* = 5 mice per group, ****P < 0.0001, one-way ANOVA with Bonferroni’s multiple comparisons test. **e**,**f**, Deletion of GPR120 in PAMAs, but not in CTNs, eliminates the therapeutic effects of ALA and EDA. The latency and the length of swim path to reach a hidden platform during the training sessions and the percentage of time spent in searching for a hidden platform in targeting quadrant (quadrant 2) during the probe trial of the individual PS1 mice at 11 months of age with deletion of GPR120 in PAMAs (**e**) or CTNs (**f**). The mice were treated with saline vehicle (V) or ALA and EDA (A-E). **g**,**h**, GPR120 in PAMAs mediates the therapeutic effects of A-E. Plots showing the time in novel arm (left) and the total numbers of visits to each arm (right) in the individual mice at 8 months of age with deletion of GPR120 in PAMAs (**g**) or CTNs (**h**). The mice were treated with saline vehicle or A-E. For **e**–**h**, data are presented as mean ± s.e.m., *n* = 10 mice per group. Two-way ANOVA with Bonferroni’s multiple comparisons test was used for **e** and **f**, and one-way ANOVA with Bonferroni’s multiple comparisons test for **g** and **h**, and analysis of time in **e** and **f**. *P < 0.05; **P < 0.01; ***P < 0.001; ****P < 0.0001; NS, no significant difference. The exact *P* values are reported as source data. Source data

**Extended Data Fig. 3:**
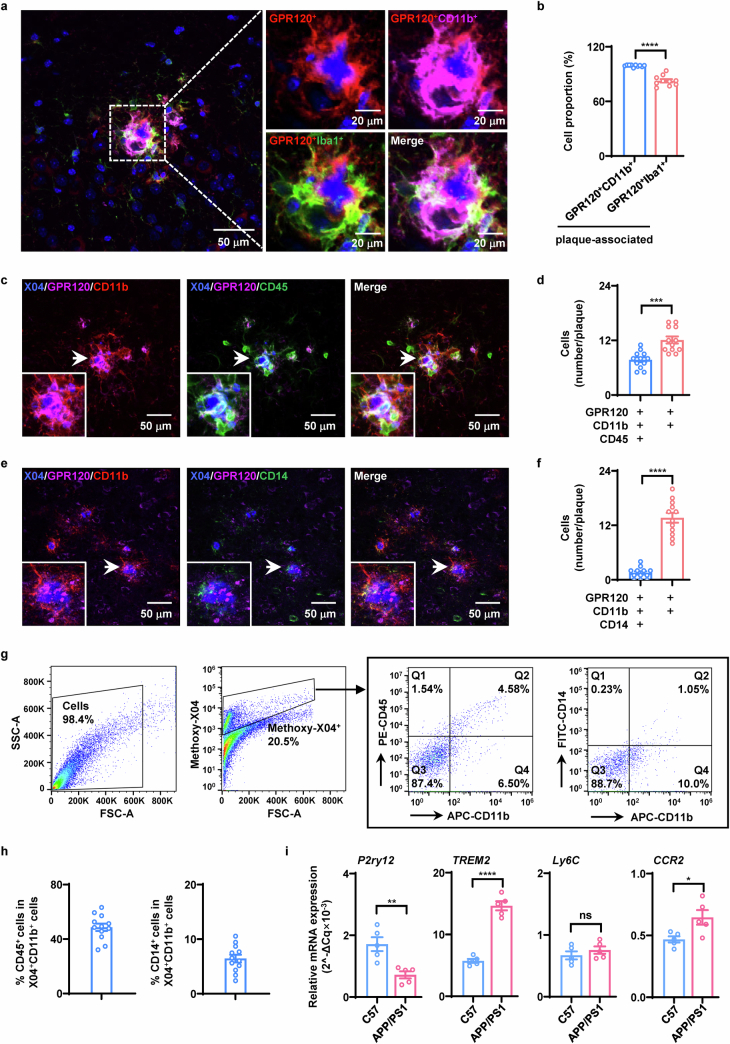
GPR120 is expressed in CD11b-positive PAMAs. **a**, Representative images showing the labeling of anti-GPR120 (red), anti-Iba1 (green) and anti-CD11b (magenta) with DAPI (blue) in the cortex of APP/PS1 mice. **b**, Quantification of the proportion of GPR120-positive cells surrounding the amyloid plaques (within 30 μm of X04^+^ plaques, plaque-associated) that colocalize with CD11b or Iba1 in the cortex of APP/PS1 mice. Data are presented as mean ± s.e.m., *n* = 10 mice per group. **c, d**, Representative images (**c**) and a plot (**d**) showing the labeling of anti-CD11b (red), anti-CD45 (green) and anti-GPR120 (magenta) with Methoxy-X04 (blue) in the cortex of APP/PS1 mice. Their merged cells indicating for CD45-positive PAMAs (merge). Data are presented as mean ± s.e.m., *n* = 12 biological replicates from 4 mice per group. **e, f**, Representative images (**e**) and a plot (**f**) showing the labeling of anti-CD11b (red), anti-CD14 (green) and anti-GPR120 (magenta) with Methoxy-X04 (blue) in the cortex of APP/PS1 mice. Their merged cells indicating for CD14-positive PAMAs (merge). Data are presented as mean ± s.e.m., n = 12 biological replicates from 4 mice per group. **g**, Gating strategy and the proportion of CD45^+^ macrophages and CD14^+^ peripheral monocytes among Aβ-associated CD11b-positive PAMAs in APP/PS1 mice. **h**, Plots showing the ratio of CD45^+^CD11b^+^ and CD14^+^CD11b^+^ cells in Aβ-associated CD11b-positive PAMAs. Data are presented as mean ± s.e.m., *n* = 12 biological replicates from 4 mice per group. **i**, Relative mRNA expression levels of the indicated markers (*P2ry12*, *TREM2*, *Ly6C, CCR2*) on CD11b-positive cells isolated from brain tissues of C57 and APP/PS1 mice at 5 months (M) old of age. Data are presented as mean ± s.e.m., *n* = 5 mice per group. Experiments were repeated at least three times independently with similar results. Unpaired Student’s t-test was used for **b**, **d**, **f** and **i**, *P < 0.05, **P < 0.01, ***P < 0.001, ****P < 0.0001 and ns, no significant difference. The exact *P* values are reported as source data for Extended Data Fig. 3. Source data

G-protein-coupled receptors (GPCRs) are important signaling molecules for many aspects of cellular function^35^. Five of them, GPR40, GPR41, GPR43, GPR84, and GPR120, are activated by free fatty acids^36^. Short-chain fatty acids are specific agonists for GPR41 and GPR43 (ref. ^37^) and medium-chain fatty acids for GPR84 (ref. ^38^). Long-chain fatty acids activate GPR40 (ref. ^39^) and GPR120 (ref. ^40^). Selective activation of GPR120 or free fatty acid receptor 4 (FFAR4) has therapeutic potential for the treatment of many diseases, including type 2 diabetes mellitus, osteoporosis, inflammation, and obesity^41–43^. Here, we found GPR120 highly expressed in integrin α M (ITGAM, also known as CD11b)-positive cells surrounding amyloid plaques^44^ (amyloid plaques-associated CD11b^+^GPR120^+^ cells), comprising mainly activated microglia (CD11b^+^Iba1^+^ cells), macrophages^45^ (CD11b^+^CD45^+^ cells), and a small fraction of monocyte-derived macrophages (CD11b^+^CD14^+^ cells) (Fig. 2a,c and Extended Data Fig. 3). These plaque-associated immune populations were collectively designated as CD11b-positive PAMAs. These data are consistent with a recent work that PAMAs include a minor subset (~6%) of GPR120-positive peripheral monocytes (CD11b^+^CD14^+^ cells) only^46^, which shows minimal effects on the formation of pathological amyloid plaques in 5×FAD mice, a model that exhibits high plaque burden in the cortex, hippocampus, and thalamus of 6-month-old mice^47,48^ (Supplementary Fig. 5). By contrast, CD11b-negative microglia distal to plaques rarely expressed GPR120 (plaque-disassociated CD11b^−^Iba1^+^GPR120^−^ cells; Fig. 2a,c). Beyond PAMAs, GPR120 was also found to be expressed in vesicular glutamate transporter-2 (vGlut2^+^) cortical neurons (CTNs) that were sparsely distributed in the prefrontal cortex (Fig. 2b,c and Supplementary Fig. 6). To gain molecular insights into the actions of ALA and EDA, we set out to determine whether the therapeutic effects of ALA and EDA could be achieved through activation of a cell-type-specific GPR120 in either PAMAs or CTNs.

We generated mouse models that conditionally delete GPR120 in either PAMAs or CTNs. GPR120^loxP^ mice (Supplementary Fig. 7) were crossed with APP/PS1 and CTN^CreERT2+/+^, in which the Cre recombinase fused to a mutant estrogen receptor ligand-binding domain (CreERT2) was expressed under the control of calcium/calmodulin-dependent protein kinase II α (CaMKIIα) promoter, or PAMA^CreERT2+/+^ mice, in which CreERT2 was driven by *CD11b* promoter. Administration of tamoxifen in the mice at 5 months of age resulted in the production of APP/PS1-CTN^GPR120−/−^ and APP/PS1-PAMA^GPR120−/−^ mice, in which GPR120 was deleted in CTNs or PAMAs of APP/PS1 mice (Fig. 2d).

The behavioral performance in the Morris water maze and the novel arm Y- maze tests exhibited a similar degree of the impairments in both APP/PS1-CTN^GPR120−/−^ and APP/PS1-PAMA^GPR120−/−^ mice, showing that deletion of GPR120 itself does not affect the performance of the mice (Fig. 2e–h). Administration of exogenous ALA and EDA led to a notable improvement of learning and memory in APP/PS1-CTN^GPR120−/−^, APP/PS1-CTN^GPR120+/+^ and APP/PS1-PAMA^GPR120+/+^ mice, based on the Morris water maze (Fig. 2e,f) and the novel arm Y-maze tests (Fig. 2g,h), while the same treatment was entirely without effect or much less effective in protecting against the memory declines of APP/PS1-PAMA^GPR120−/−^ mice (Fig. 2e,g). Thus, a cell-type-specific GPR120 in PAMAs, but not in CTNs, mediates the therapeutic effects of ALA and EDA.

GPR120 is also expressed in peripheral tissues^40^. We then performed experiments by intraventricular application of tamoxifen in APP/PS1-PAMA^GPR120−/−^ mice. We found that this application effectively deleted GPR120 in PAMAs, without affecting GPR120 in the intestinal tissues (Supplementary Fig. 8a,b). Particularly, we found that this application generated pathological and behavioral phenotypes in APP/PS1 mice, similar with those induced by systematic application of tamoxifen (Supplementary Fig. 8c,d), and hence excluded the peripheral contributions.

### Structures of ALA and EDA bound to GPR120

Recent advancements in structural biology, particularly through cryo-EM studies, have yielded profound insights into the structural dynamics of GPR120 in complex with various ligands^22^. These studies have elucidated the active conformational states of GPR120 bound to different effector G proteins. In parallel, computational methods such as Alpha-fold have provided valuable predictions of the inactive state of GPR120. Here, we aimed to bridge the knowledge gap between the inactive and active conformational states of GPR120 and elucidate the molecular mechanisms underlying its interaction with ALA and EDA. We then solved the cryo-EM structure of an ALA and EDA-bound GPR120–Gi (guanine nucleotide-binding inhibitory protein) complex at 3.0 Å. The cryo-EM densities allowed assignment of the bound ligands and most residues of the GPR120 receptor and Gi heterotrimer. The binding modes of the ligands in the corresponding structures were further supported by molecular dynamics simulations, as described below.

**Fig. 3:**
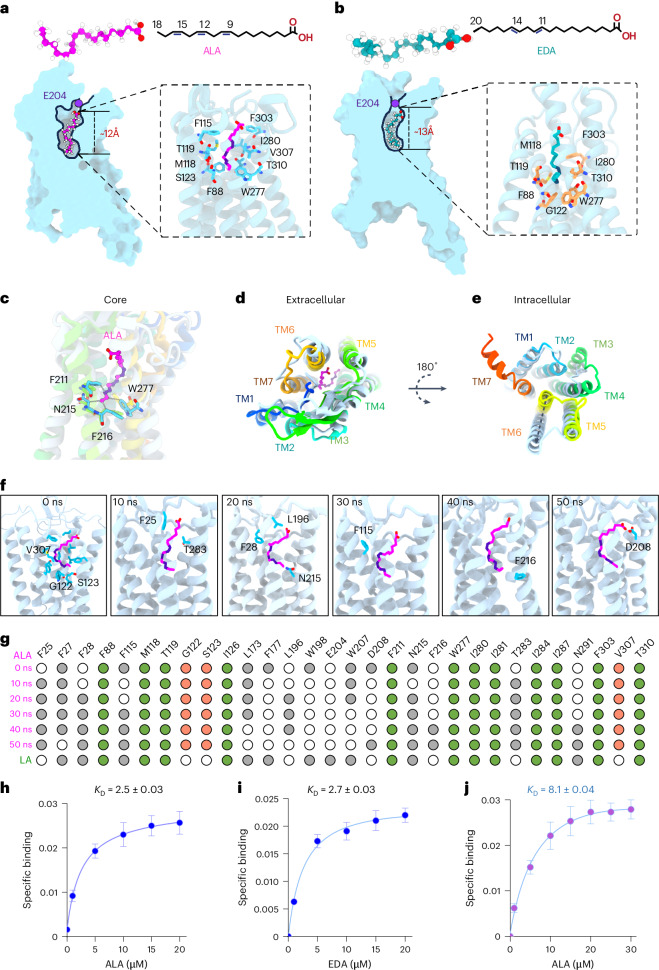
Structures of GPR120 bound to ALA and EDA. **a**,**b**, Binding of ALA (**a**) and EDA (**b**) within the ligand binding pocket. The enlarged view highlights the interactions involving the unsaturated bonds with GPR120. **c**–**e**, ALA activates GPR120. Structures of ALA bound to GPR120, with the core (**c**), the extracellular (**d**), and the intracellular views (**e**), generated by Alpha-fold, as shown in rainbow color. Residues participating in the interactions with ALA C-terminal are labeled in **c**. The gray dashed line illustrates the notable changes in TM6 between the ALA-activated GPR120 and the Alpha-fold-predicted inactive form in **e**. **f**, The binding pocket residues of GPR120 interacting with ALA. Throughout the 50 ns simulation, residues V307, G122, and S123 exhibit stable interactions with ALA, while other residues show variable interactions. **g**, Dot plot visualization of the binding-pocket residues of GPR120 involved in interactions with the ligand ALA. Green dots denote stable interactions consistent with the cryo-EM structure of linoleic acid bound to GPR120 (PDB 8ID6), while orange dots indicate stable interactions observed in our simulation. Gray dots represent dynamic interactions. **h**–**j**, Saturation binding curves of FITC–ALA (**h**) or FITC–EDA (**i**) or FITC–ALA in the presence of 10 μM EDA (**j**) toward GPR120. Data are presented as mean ± s.e.m. from three independent experiments performed in triplicates (*n* = 9). Source data

**Extended Data Fig. 4:**
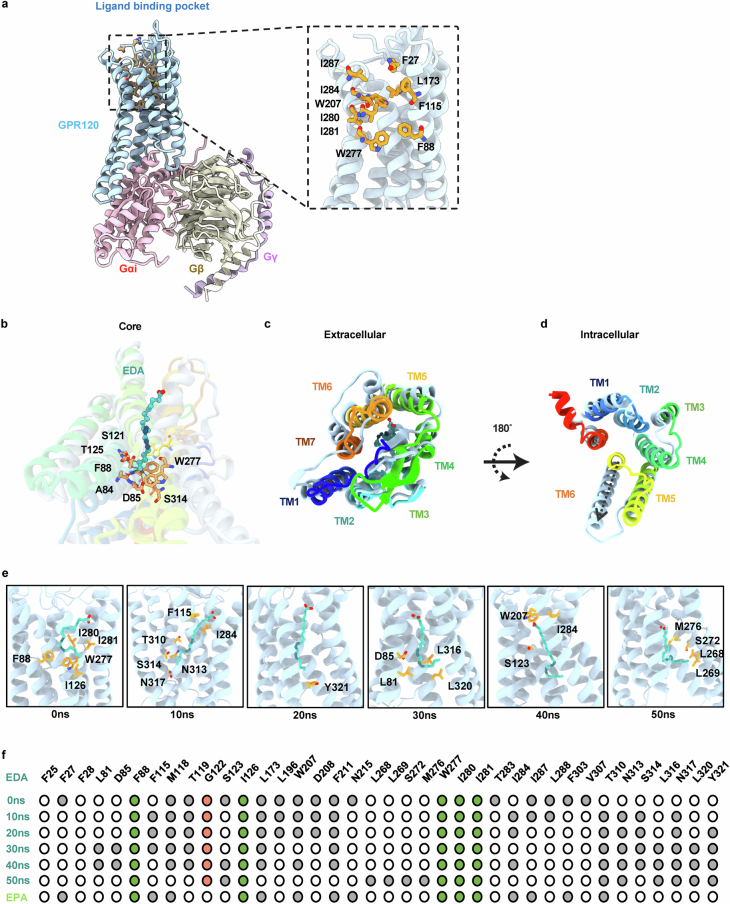
Structures of GPR120 bound to EDA. **a**, The entire system comprising GPR120 and Gαi1/Gβ/Gγ complex was illustrated. The ligand binding pocket was delineated by a dashed line box, while the enlarged view on the right emphasized the hydrophobic amino acids within the binding pocket. **b, c**, EDA activates GPR120. Structures of EDA bound to GPR120, with the core (**b**), the extracellular (**c**) and the intracellular views (**d**). Residues involved in the interactions with the EDA C-terminal are labeled in panel (**d**). The gray dashed line indicates the notable alterations in TM6 between the EDA-activated GPR120 and the AF-predicted inactive form (**d**). **e**, Illustrates the binding pocket residues of GPR120 interacting with EDA. Throughout the 50 nanosecond simulation, residues G122 exhibit stable interactions with EDA in our experiment, while other residues demonstrate variable interactions. **f**, A dot-plot of the binding-pocket residues of GPR120 involved in interactions with EDA. Green dots denote stable interactions consistent with the cryo-EM structure of EPA bound to GPR120 (PDB:8ID4), while orange dots indicate stable interactions observed in our simulation. Gray dots represent dynamic interactions.

Through molecular dynamics simulations, we explored the intricate details of how the combined administration of ALA and EDA, rather than either of them alone, exerts therapeutic efficacy against the progression of AD. We constructed complexes with the α subunits of inhibitory (Gαi), phospholipase C-activating (Gαq), and stimulatory (mini-Gαs) G proteins (Supplementary Table 3). Subsequently, these initial complexes were embedded within a simplified phosphatidylcholine bilayer environment. GPCRs also interact with cholesterol and anionic lipids in the membrane. Accordingly, we first applied phosphatidylcholine as a model bilayer and later incorporated ALA and EDA into the lipid bilayer composite to mimic a native membrane environment (Supplementary Table 4). Our data revealed that ALA or EDA adopted an “L” shape and stably bound to GPR120 in the ligand-binding pocket. This pocket was composed of hydrophobic amino acids including F27, F88, F115, L173, W207, W277, I280, I281, I284, and I287, which provided ample space for accommodating ALA and EDA (Extended Data Fig. 4a). ALA extended deeply into the pocket, with a maximal depth of about 12 Å (Fig. 3a), while EDA exhibited additional π–π interactions (characterized by dispersive attraction between π-electron clouds) between C11 and C14, penetrating the pocket with a maximum depth of approximately 13 Å (Fig. 3b). The ligand-binding pocket of GPR120 was dictated by residue E204, which can establish a water-mediated hydrogen-bond network with adjacent residues and the hydrophobic aryl chain ends of the ligand’s C-terminal and hence effectively prevent ligand dissociation. Encircling ALA and EDA was multiple π–π stacking interactions with residues like F88, F115, W277, and F303 for ALA (containing three double bonds) (Fig. 3a), and F88, W277, and F303 for EDA (containing two double bonds) (Fig. 3b). The stability of ALA bound to GPR120 was bolstered by hydrophobic interactions involving M118, I280, and V307, along with polar interactions involving T119, S123, and T310 (Fig. 3a). Similarly, the binding stability of EDA was reinforced by hydrophobic interactions involving M118, G122, and I280, as well as polar interactions involving T119 and T310 (Fig. 3b).

In the core region, the N-terminal of ALA is effectively anchored by residues F211, N215, F216, and W277 (Fig. 3c). Although the fifth and sixth transmembrane segments of GPR120 (TM5 and TM6) constituted a larger opening tunnel, facilitating ligand entry and exit^49^, the π–π stacking interactions firmly secured ALA in place. EDA had an elongated C-terminal (C19–C20) and thus enhanced insertion, altering the surrounding residues including A84, D85, F88, S121, T125, and S314 (Extended Data Fig. 4b). In addition, W277 was implicated in establishing an extensive π–π interaction network over long distances to ensure closure of the bottleneck side. We then overlaid ALA or EDA binding onto the active structure of GPR120 with the Alpha-fold-predicted inactive structure of GPR120. We observed a substantial outward movement at the extracellular side (Fig. 3d and Extended Data Fig. 4c). The most important conformational change was involved in the outward movement of TM6 (Fig. 3e, Extended Data Fig. 4d, and Supplementary Videos 1 and 2). Consistent with the findings from cryo-EM studies revealing linoleic acid binding to GPR120 (PDB 8ID6), we observed a pivotal role of the hydrophobic interaction network in facilitating ALA binding. Certain residues, including F25, F28, F115, F216, D208, N215, and T283, exhibit dynamic involvement in the interaction with GPR120 (Fig. 3f). Furthermore, our investigation identified residues G122 and S123, which engage in backbone Van Der Waals forces interactions with ALA, while residue V307 interacted with ALA through hydrophobic interactions (Fig. 3f,g). Subsequent mutation of V307 to a hydrophilic residue led to a notable alteration in binding affinity. Additional dynamic interactions were observed in the EDA-bound state (Extended Data Fig. 4e,f), with the noteworthy involvement of G122 in backbone Van Der Waals forces interactions with EDA, mirroring its interaction with ALA (Extended Data Fig. 4f). Consistent with this conformational change, the ligand binding assay revealed that ALA and EDA can bind to GPR120 (Fig. 3h,i), while in the presence of EDA, the affinity of ALA binding to GPR120 was robustly increased (Fig. 3j).

### Allosteric modulation and synergistic activation of GPR120

**Extended Data Fig. 5:**
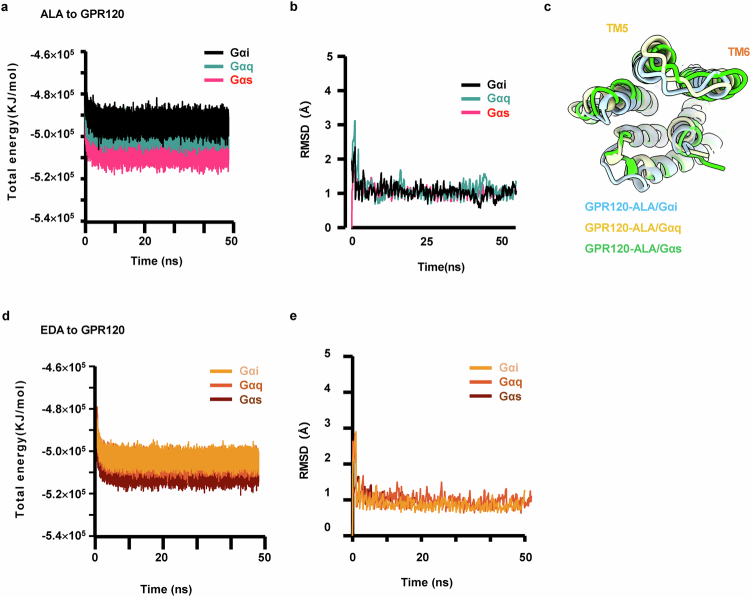
MD simulations of ALA or EDA bound to GPR120-Gα. **a**, MD simulations of ALA bound to GPCR120 in complex with Gαi, Gαq, and Gαs. Total energy associated with Gαs binding was the lowest, followed by Gαq and Gαi1. **b**, The root mean square deviation (RMSD) value of ALA extracted from a 50-ms MD simulation between GPR120 and ALA with different G proteins. **c**, The distance between TM6 and TM7 was larger in the case of Gαs binding compared to the other two scenarios. **d, e**, MD simulations of EDA bound to GPCR120 in complex with Gαi, Gαq, and Gαs consistently demonstrate total energy values similar to those of ALA (**d**) and stable RMSD variation (**e**).

Various G proteins engage with GPR120 to regulate energy and suppress inflammation^41,50^. Accordingly, we investigated the interactions between GPR120 and ALA with different G proteins. Our analysis indicated that the total energy associated with Gαs binding was the lowest, followed by Gαq and Gαi (Extended Data Fig. 5a,b). Upon superimposing the three complexes, we observed that the distance between TM6 and TM7 was larger in the case of Gαs binding, compared to the other two scenarios (Extended Data Fig. 5c). Similar to ALA, EDA bound to GPR120 in the ligand binding pocket. The total energy associated with Gαs binding was the lowest, followed by Gαi and Gαq (Extended Data Fig. 5d,e).

**Fig. 4:**
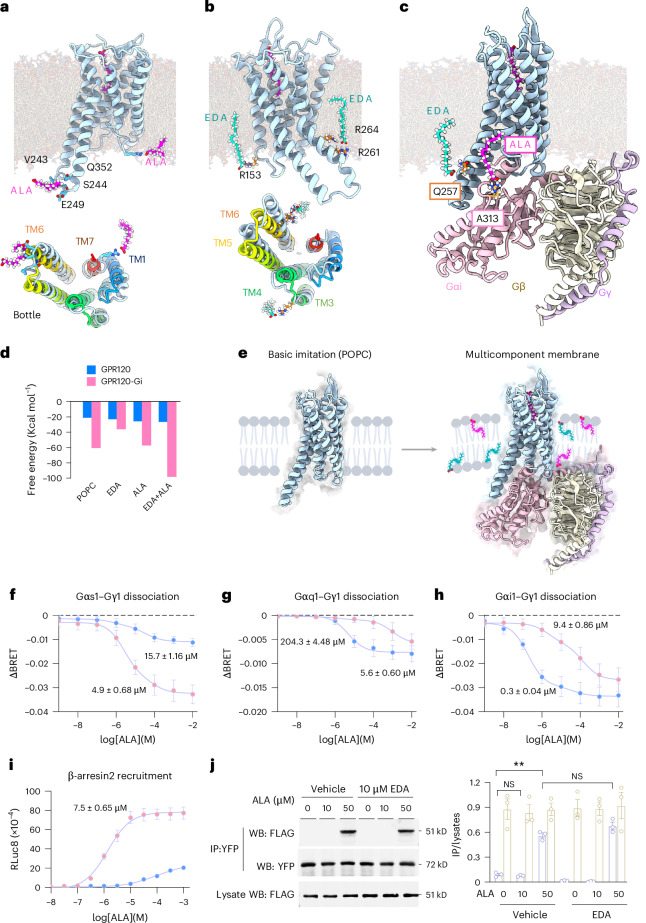
Allosteric modulation and synergistic activation of GPR120. **a**, ALA in the lipid membrane encircling GPR120 causes the conformational changes of transmembrane helices 6 and 7 (TM6 and TM7). **b**, EDA in the lipid membrane encircling GPR120 causes the conformational changes between TM3 and TM4, as well as TM6 and TM7. **c**, EDA binds to GPR120 between TM6 and TM7. ALA engages with Gαi through charge–charge interactions. **d**, EDA modulates the interaction of ALA to GPR120 and Gαi. A bar graph showing free binding energies of ALA toward GPR120 and GPR120 interaction with Gαi under different membrane composition conditions. **e**, Illustrations showing that GPR120 is inserted into a POPC membrane and subsequently transferred into a multicomponent membrane containing ALA and EDA. The presence of ALA and EDA allosterically modulates and ultimately enhances GPR120 interaction with Gαi. **f**–**h**, Dose–response curves of GPR120 in response to stimulation with ALA in the absence (pink) or the presence of EDA (blue) by Gαs1–Gγ1 dissociation (**f**), Gαq1–Gγ1 dissociation (**g**) or Gαi1–Gγ1 dissociation (**h**). Data are mean ± s.e.m. from *n* = 5 independent experiments. EC_50_ values are marked in each panel. **i**, Dose–response curves of β-arrestin2 recruitment in response to stimulation with ALA in the absence (pink) or the presence of EDA (blue). Data are presented as mean ± s.e.m. from *n* = 3 independent experiments. EC_50_ values are marked in each panel. **j**, Co-immunoprecipitation between GPR120 (YFP-labeled, gold plots) and β-arrestin2 (FLAG-labeled, light purple plots) with ALA stimulation in the presence of vehicle control or EDA in HEK 293 cells. Quantification was performed by normalizing the immunoprecipitation (IP) signal relative to the corresponding input lysate. Data are presented as mean ± s.e.m. from *n* = 3 independent experiments. **P < 0.01; NS, no significant difference, one-way ANOVA with Tukey’s multiple comparisons test. The exact *P* values are reported as source data. WB, western blot; FLAG, FLAG epitope tag; YFP, yellow fluorescent protein. Source data

**Extended Data Fig. 6:**
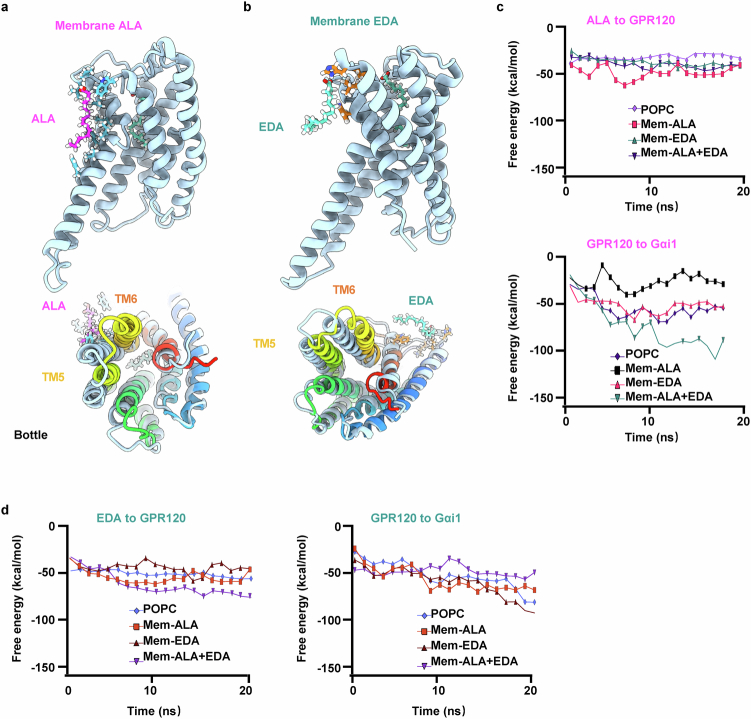
EDA allosterically modulates ALA binding to GPR120. **a, b**, MD simulation spanning 50-ns showing that EDA bound to GPR120 increases ALA molecules surrounding GPR120 in the membrane environment, affecting the conformation of TM5 and TM6 (**a**). Similarly, EDA present in the membrane environment influences the regions between TM5 and TM6 of GPR120 (**b**). **c, d**, Free binding energy profiles of ALA to GPR120 (**c, top**), GPR120 to Gαi (**c, bottom**), and EDA to GPR120 (**d, left**), GPR120 to Gαi (**d, right**) under conditions of pure POPC membrane, membrane-inserted ALA, membrane-inserted EDA, and membrane-inserted ALA and EDA.

Previous studies highlighted the role of membrane phospholipids in allosterically modulating the activity of GPCRs^51^. This modulation influences the binding affinity of cholesterol with GPCRs^52^, suggesting a broader impact beyond traditional allosteric modulation. As a molecule with both hydrophilic and hydrophobic properties, ALA can permeate the lipid bilayer of cell membranes, regulate membrane-bound receptors and transporters, and possibly affect cellular metabolism, energy production, and lipid homeostasis^53^. To explore the impact of ALA and EDA on membrane dynamics, we introduced these molecules into the membrane component and conducted molecular dynamics simulation. We found that ALA in the membrane environment of the receptor caused a marked separation of TM6 (Fig. 4a and Extended Data Fig. 6a), whereas EDA exerted forces on TM6, as well as the loop between TM3 and TM4 (Fig. 4b and Extended Data Fig. 6b). When both ALA and EDA were present in the membrane at a ratio of 1:10, ALA was engaged with Gαi through the hydrogen bonding between A313 and ALA carboxyl group, introducing an anchoring point for Gαi protein membrane docking (Fig. 4c). Concurrently, EDA in the membrane environment interacted with Q257 and caused further displacement of TM6 from TM7, creating additional space for Gαi binding (Fig. 4c and Supplementary Video 3).

Using molecular dynamics simulation (Extended Data Fig. 6c,d) with ligand binding assay (Fig. 3h–j), we conclude that membrane ALA and EDA binds to the allosteric sites of GPR120 and robustly enhances the affinity of ALA binding to GPR120 (Fig. 4d). This allosteric effect facilitates the interaction of GPR120 with downstream Gαi protein (Fig. 4e). These findings contribute to a deeper understanding of the structural interplay between cell membrane components and GPCR function and provide insights into the development of druggable allosteric agonists of a cell-type-specific GPR120 for the precision treatment of AD.

### ALA and EDA synergistically activate GPR120–Gαi1–mTORC1

**Extended Data Fig. 7:**
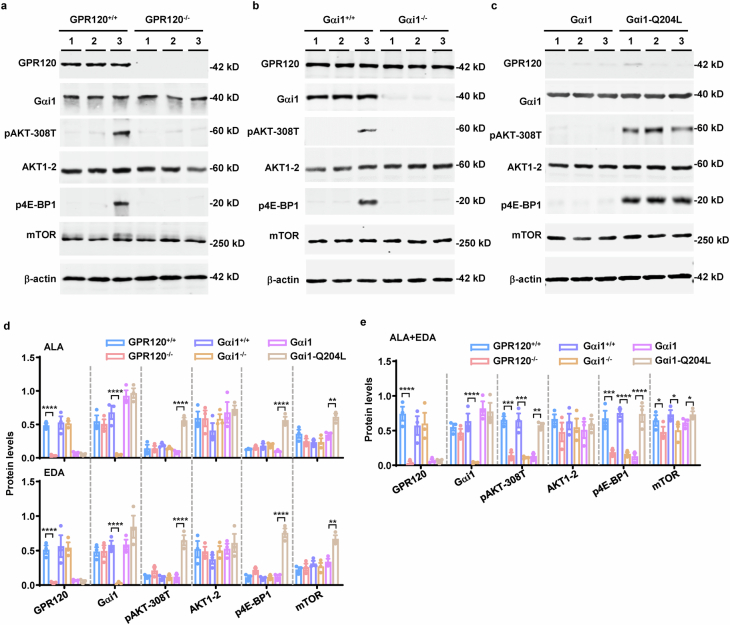
ALA with EDA activate Gαi1-AKT-mTORC1 pathway. **a-c**, Representative blots showing protein levels in HEK 293 cells which were GPR120 positive (GPR120^+/+^) and GPR120 negative (GPR120^−/−^) (**a**), or Gαi1 positive (Gαi1^+/+^) and Gαi1 negative (Gαi1^−/−^) (**b**) or GPR120 negative cells with overexpressing Gαi1 or Gαi1-Q204L (**c**) in response to stimulation with ALA (lane 1), EDA (lane 2) or ALA + EDA (lane 3). **d, e**, Plots showing the protein levels normalized to the respective β-actin, defined as 1.0, in cells in response to stimulation with ALA (top, **d**), EDA (bottom, **d**) or ALA together with EDA (**e**). Data are presented as mean ± s.e.m. from three independent experiments (*n* = 3) performed in triplicates. Two-way ANOVA with Tukey’s multiple comparisons test was used. *P < 0.05, **P < 0.01, ***P < 0.001, ****P < 0.0001. The exact *P* values are reported as source data for Extended Data Fig. 7. Source data

We next performed studies in vitro to determine the biological consequences of GPR120 allosteric modulation. We established a cell-based reporter system by transfecting human embryonic kidney (HEK) 293 cells with constructs for GPR120 along with a serum response element-luciferase promoter/reporter (RLuc8). GPR120 couples to several transducers to mediate different downstream signals including Gαs–cAMP (cyclic adenosine monophosphate), Gαi1–cAMP, Gαq–IP3 (inositol triphosphate), and β-arrestin2 (ref. ^54^). To determine potential signaling actions and evaluate the G-protein selectivity landscape of GPR120 with ALA and EDA, we implemented a cell-based bioluminescence resonance energy transfer (BRET) assay. ALA and EDA were profiled against these multiple transducers by treating the reporter cells with ALA or EDA alone, or ALA together with EDA (Fig. 4f–j). ALA alone preferentially activated the Gαs–cAMP pathway, which had little effects on Gαq–IP3 signaling, whereas EDA showed Gαi-biased activity only when a high dose was applied (Fig. 4f–h and Supplementary Fig. 9). A combination of ALA with EDA did not affect β-arrestin2 recruitments (Fig. 4i), but it allowed GPR120 to activate a cAMP-independent non-canonical Gαi1 signaling pathway (Fig. 4j and Extended Data Fig. 7). This included protein kinase B (AKT) and metabolic regulator protein kinase B–mTORC1, as marked by phosphorylation of eukaryotic translation initiation factor 4E-binding protein 1 (p-4E-BP1). This activation was completely abolished by deletion of GPR120 or Gαi1 and rescued by introduction of a Gαi1 constitutively active mutant Gαi1–Q204L (glutamine (Q) at position 204 of replaced by leucine (L)) (Extended Data Fig. 7). Together, these data show that combination of ALA and EDA enables GPR120 activation of non-canonical Gαi1–mTORC1 signaling.

### ALA with EDA inhibits amyloid pathology via GPR120–Gαi1–mTORC1

**Fig. 5:**
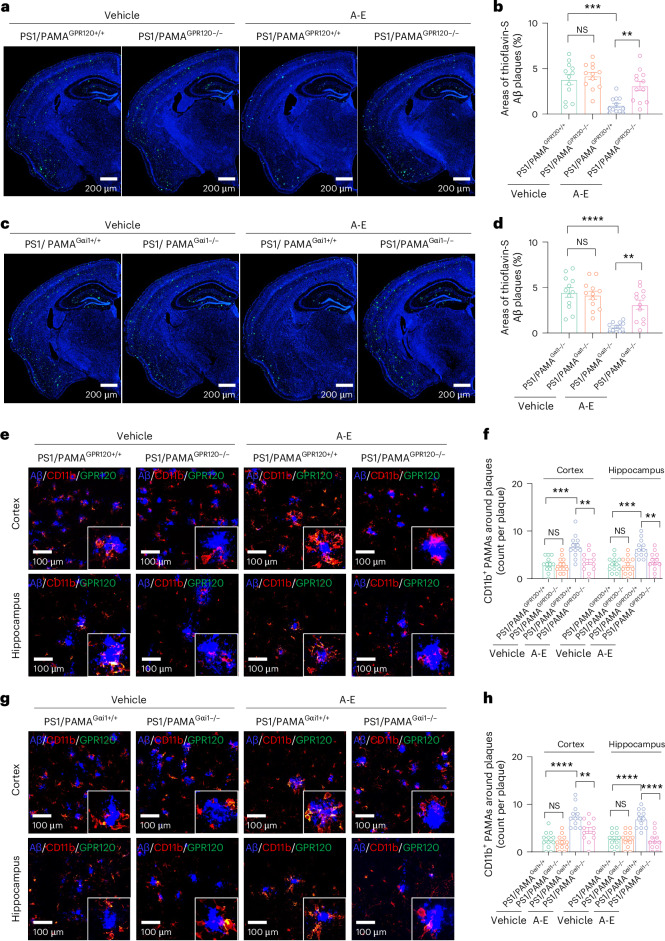
ALA and EDA attenuate Aβ pathology via GPR120–Gαi1 in PAMAs. **a**–**d**, ALA and EDA attenuate Aβ plaques by activation of GPR120–Gαi1. Representative images (**a** and **c**) showing brain sections stained with thioflavin-S (green). DAPI was used for nuclear staining (blue). Plots (**b** and **d**) showing the areas of thioflavin-S-labeled amyloid plaques throughout the cortex and the hippocampus of PS1/PAMA^GPR120+/+^ and PS1/PAMA^GPR120−/−^ mice (**a** and **b**), or PS1/PAMA^Gαi1+/+^ and PS1/PAMA^Gαi1−/−^ mice (**c** and **d**) at 8 months of age after administration of ALA and EDA (A-E) for 45 consecutive days. Data are presented as mean ± s.e.m., *n* = 12 slides from 6 mice per group. **P < 0.01; ***P < 0.001; ****P < 0.0001; NS, no significant difference, one-way ANOVA with Bonferroni’s multiple comparisons test. **e**–**h**, ALA and EDA activate and recruit PAMAs into Aβ plaques. Representative images (**e** and **g**) and plots (**f** and **h**) showing Aβ plaque-associated PAMAs (within 30 μm of Aβ plaques) labeled with anti-CD11b in the cortex and hippocampus of PS1/PAMA^GPR120+/+^ and PS1/PAMA^GPR120−/−^ mice (**e** and **f**) or PS1/PAMA^Gαi1+/+^ and PS1/PAMA^Gαi1−/−^ mice (**g** and **h**) at 8 months of age. The mice at 5 months of age were treated with control vehicle or A-E for 45 consecutive days. Brain sections were prepared from the mice at 8 months of age and stained with anti-GPR120 (green), anti-CD11b (red) and anti-Aβ_1-16_ (blue). Data are presented as mean ± s.e.m., *n* = 12 slides from 3 mice per group. **P < 0.01; ***P < 0.001; ****P < 0.0001; NS, no significant difference, one-way ANOVA with Bonferroni’s multiple comparisons test. Experiments were repeated at least three times independently with similar results. The exact *P* values are reported as source data. Source data

Having determined that allosteric modulation and synergistic activation of GRP120 generates the therapeutic effects against learning and memory declines in AD mice, we next wanted to examine the mechanisms underlying how these effects are achieved. Aβ plaques are among the major pathological hallmarks of AD^55,56^. Accordingly, we examined the effects of ALA and EDA on Aβ pathology and AD-related pathways in vivo. Both APP/PS1-PAMA^GPR120−/−^ and APP/PS1-PAMA^GPR120+/+^ mice at 5 months of age were treated with ALA and EDA once per day for 45 consecutive days, and brain sections were collected at the age of 8 months. We found that this treatment reduced the amyloid burden in the brain, whereas treatment with vehicle control had no detectable effects (Fig. 5a,b and Supplementary Fig. 10a,b). Similarly, levels of soluble and insoluble Aβ_1-42_ were lowering in AD mice treated with ALA and EDA (Supplementary Fig. 11a–c). The protective effects of ALA and EDA remained detectable in AD mice at 8 months of age and was completely eliminated by deletion of Gαi1 in APP/PS1-PAMA^Gαi1−/−^ mice (Fig. 5c,d and Supplementary Fig. 10c,d). Furthermore, immunoblotting of cortical homogenates showed that treatment with EDA and ALA did not affect the levels of full-length amyloid precursor protein (APP), cleaved C-terminal fragments (APP-CTFs), and APP-processing/Aβ-degrading enzymes, including PS1, BACE1, ADAM17 and insulin-degrading enzyme (Supplementary Fig. 11d,e). These data indicate that APP processing was not perturbed by either administration of ALA and EDA or deletion of GPR120 or Gαi1.

**Fig. 6:**
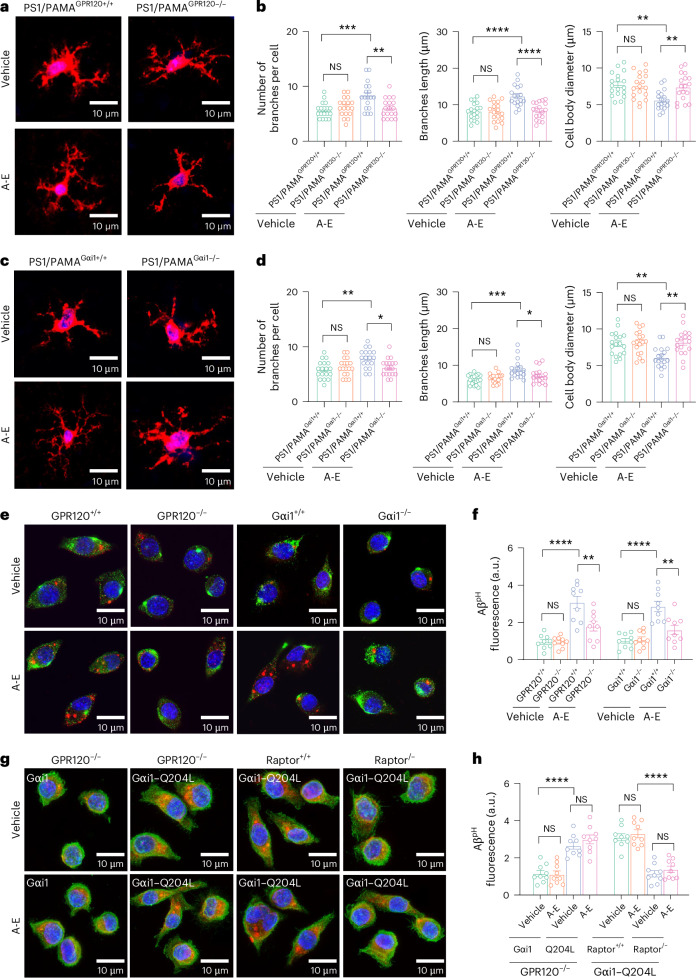
Activation of PAMAs by ALA and EDA promotes amyloid phagocytosis. **a**–**d**, Representative images (**a** and **c**) and plots (**b** and **d**) showing the morphologies of PAMAs in the cortex of PS1/PAMA^GPR120+/+^ and PS1/PAMA^GPR120−/−^ mice (**a** and **b**), or PS1/PAMA^Gαi1+/+^ and PS1/PAMA^Gαi1−/−^ mice (**c** and **d**). Mice at 5 months of age were treated with control vehicle or A-E for 45 consecutive days. Brain sections were prepared from the mice at 8 months of age and stained with anti-CD11b (red). DAPI was used for nuclear staining (blue). The number of branches, average branch length and cell body diameter were quantified. Experiments were repeated at least three times independently with similar results. Data are presented as mean ± s.e.m., *n* = 18 cells from 6 mice per group. *P < 0.05; **P < 0.01; ***P < 0.001; ****P < 0.0001; NS, no significant difference, one-way ANOVA with Bonferroni’s multiple comparisons test. **e**,**f**, Representative images (**e**) and a plot (**f**) showing phagocytosed Aβ^pH^ (red) in the cultured PAMAs (CD11b^+^, green) from PAMA^GPR120+/+^ and PAMA^GPR120−/−^, or PAMA^Gαi1+/+^ and PAMA^Gαi1−/−^ mice at 3 months of age. The cultured PAMAs were treated with vehicle or A-E for 10 min, and then pHrodo Red-labeled Aβ1-42 oligomers (Aβ^pH^) were added. After 2 h incubation, the phagocytosed Aβ^pH^ fluorescence was quantified and normalized to the baseline. **g**,**h**, Representative images (**g**) and a plot (**h**) showing phagocytosed Aβ^pH^ (red) in the cultured PAMAs (green) from PAMA^GPR120−/−^, PAMA^Raptor+/+^, or PAMA^Raptor−/−^ mice at 3 months of age. The cultured PAMAs were expressed with exogenous Gαi1 or Gαi1–Q204L using the Lenti-CMV–Gαi1–IRES-GFP or Lenti-CMV–Gαi1–Q204L–IRES-GFP virus particles (0.1 μl of 6.5 × 10^13^ genomic particles per ml). The cultures were treated with vehicle or A-E for 10 min, and then pHrodo Red-labeled Aβ_1-42_ oligomers (Aβ^pH^) were added. After 2 h incubation, the phagocytosed Aβ^pH^ fluorescence was quantified and normalized to the baseline. For **e**–**h**, data are presented as mean ± s.e.m. from 3 independent experiments performed in triplicates (*n* = 9); one-way ANOVA with Bonferroni’s multiple comparisons test was used. **P < 0.01; ****P < 0.0001; NS, no significant difference. The exact *P* values are reported as source data. Source data

To determine how ALA and EDA affect function of PAMAs in the context of Aβ pathology, we assessed activation and migration of PAMAs to Aβ plaques. We evaluated the plaque-associated PAMAs. We found that the densities of the PAMAs clustering were considerably increased from AD mice between the treatments with vehicle and ALA and EDA, with over 50% increase in the number of GPR120-positive PAMAs in both cortex and hippocampus (Fig. 5e,f). This increase of PAMAs surrounding the plaques was completely ablated by deletion of GPR120 or Gαi1 in PAMAs (Fig. 5e–h). We also found that AD mice treated with ALA and EDA had greater ramified PAMAs, with longer and many more branches and decreased size of the cell body (Fig. 6a–d) in the cortex, compared to those from mice treated with vehicle, suggesting that ALA and EDA activate PAMAs.

**Extended Data Fig. 8:**
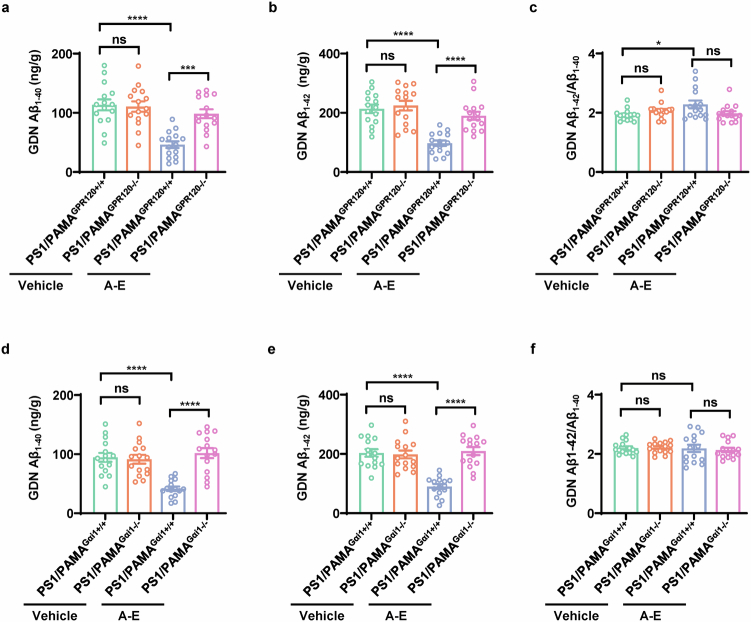
Deletion of GPR120 or Gαi1 blocks the effects of ALA and EDA on Aβ accumulation. **a**-**c**, Plots showing insoluble Aβ_1-40_ (**a**, GDN Aβ_1-40_) and Aβ_1-42_ (**b**, GDN Aβ_1-42_) peptides levels and their ratios (**c**) in the cortical tissues from PS1/PAMA^GPR120+/+^ and PS1/PAMA^GPR120−/−^ mice treated with vehicle or ALA + EDA (A-E). **d**-**f**, Plots showing GDN Aβ_1-40_ (**d**) and Aβ_1-42_ (**e**) levels and their ratios (**f**) in the cortical tissues from PS1/PAMA^Gαi1+/+^ and PS1/PAMA^Gαi1−/−^ mice treated with vehicle or A-E. Data are presented as mean ± s.e.m., *n* = 15 biological replicates from 5 mice per group. One-way ANOVA with Bonferroni’s multiple comparisons test was used. *P < 0.05, ***P < 0.001, ****P < 0.0001, ns, no significant difference. The exact *P* values are reported as source data for Extended Data Fig. 8. Source data

Next, we investigated the mechanisms underlying how activation of PAMAs affects amyloid pathology. Early studies indicated that macrophages and microglia contribute to amyloid clearance through the phago-lysosome pathway^57,58^. However, the specific role of PAMAs in amyloid pathogenesis is still unknown. We hypothesized that ALA and EDA activate GPR120–Gαi1–mTORC1 in PAMAs, promote Aβ phagocytosis, and hence attenuate the disease progression of AD. To test this hypothesis, we assessed internalization of Aβ-pHrodo (Aβ^pH^) in PAMAs from PAMA^GPR120−/−^ and PAMA^Gαi1−/−^ mice and the respective control PAMA^GPR120+/+^ and PAMA^Gαi1+/+^ mice. Treatment with ALA and EDA stimulated Aβ phagocytosis and clearance (Fig. 6e,f and Supplementary Fig. 12) and robustly reduced insoluble Aβ_1-40_ and Aβ_1-42_ levels in the brain of APP/PS1-PAMA^GPR120+/+^ and APP/PS1-PAMA^Gαi1+/+^ mice (Extended Data Fig. 8), whereas none of these effects were observed by application of ALA and EDA in PAMA^GPR120−/−^ (Supplementary Fig. 12) or PAMA^Gαi1−/−^ cells (Fig. 6e,f and Extended Data Fig. 8), showing that deletion of GPR120 or Gαi1 in PAMAs completely eliminates the beneficial effects of ALA and EDA in amyloid phagocytosis and clearance. Particularly, this deletion was completely corrected by engineering PAMA^GPR120−/−^ cells with the expression of a constitutively active mutant Gαi1–Q204L, but not Gαi1 (Fig. 6g,h).

**Extended Data Fig. 9:**
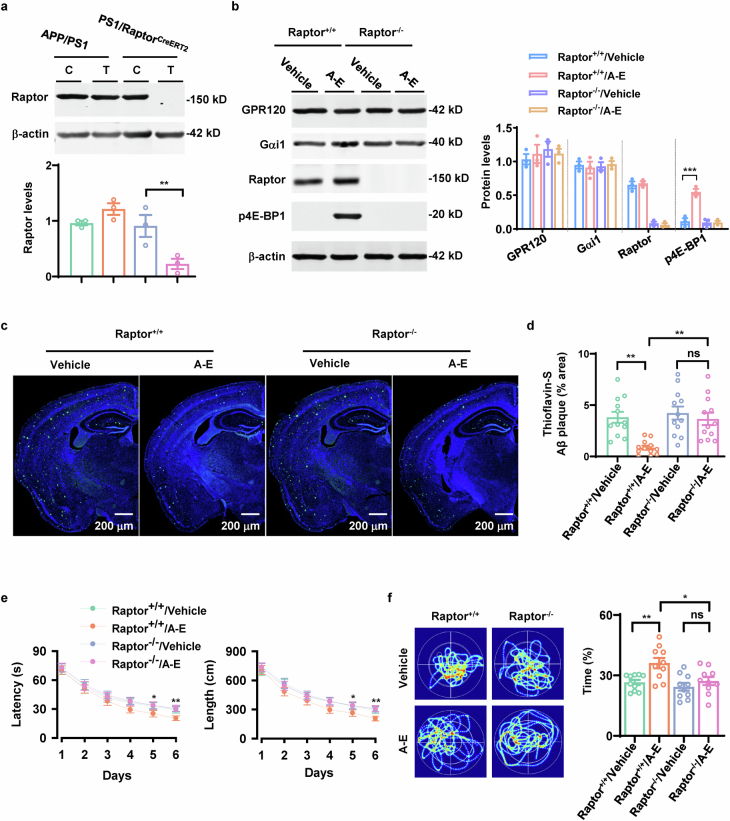
Raptor deletion blocks the therapeutic effects of ALA and EDA in AD mice. **a**, Raptor^LoxP^ mice were crossed with hCD11b-CreERT2 knock-in and APP/PS1 mice, resulting in the APP/PS1/PAMA^RaptorERT2^ mice. The mice were then administered with saline (lane C) or tamoxifen (lane T). Representative blots and a plot showing a selective deletion of Raptor protein in PAMAs. Data are presented as mean ± s.e.m., *n* = 3 mice per group. **b**, Representative blots and a plot showing that deletion of Raptor inactivates mTORC1 in PAMAs of AD mice. Data are presented as mean ± s.e.m., *n* = 3 mice per group. **c, d**, Representative images **(c)** and a plot **(d)** showing the areas of thioflavin-S-labeled amyloid plaques throughout the cortex and the hippocampus of PS1/PAMAs^Raptor+/+^ and PS1/PAMAs^Raptor−/−^ mice at 8 months old of age. Data are presented as mean ± s.e.m., *n* = 12 slides from 4 mice per group. **e, f**, The latency (**e**, left) and the length (**e**, right) of swim path to reach a hidden platform during the training sessions and the representative heat maps (**f**, left) and the percentage (**f**, right) of time spent in searching of a hidden platform in targeting quadrant (quadrant 2) during the probe trial of the individual mice at 11 months old of age. In this study, the mice were treated with vehicle or A-E for 45 consecutive days. The behavioral tests were performed when the mice were at 11 months old of age. Data are presented as mean ± s.e.m., *n* = 10 mice per group. All experiments were repeated at least three times independently with similar results. One -way ANOVA with Bonferroni’s multiple comparisons test was used for **a**, **b**, **d** and **f**, and two-way ANOVA with Bonferroni’s multiple comparisons test was used in **e**. *P < 0.05, **P < 0.01, ***P < 0.001, ns, no significant difference. The exact *P* values are reported as source data for Extended Data Fig. 9. Source data

We then determined mTORC1 function in PAMAs for amyloid phagocytosis and clearance. We crossed PAMA^CreERT2+/+^ mice with Raptor^loxP^ and APP/PS1 mice. Following administration of TAX, mTORC1 scaffolding protein Raptor was deleted in PAMAs (APP/PS1-PAMA^Raptor−/−^ mice; Extended Data Fig. 9a). Raptor deficiency generated the effects similar to that produced by deletion of GPR120 or Gαi1, completely blocking the actions of ALA and EDA on p-4E-BP1 (Extended Data Fig. 9b) and Gαi1–Q204L on amyloid phagocytosis (Fig. 6g,h). More impressively, genetic deletion of Raptor in PAMAs suppressed the therapeutic effects of ALA and EDA in APP/PS1 mice (Extended Data Fig. 9c–f). Recent studies indicate that mTOR can phosphorylate mammalian autophagy-initiating kinase ULK1, a homologue of yeast *ATG1*, on Ser^757^ (p-ULK1), thereby inhibiting autophagy^59^. Here, we found, however, that incubation of PAMAs with ALA and EDA did not change the levels of p-ULK1 (Supplementary Fig. 13).

In addition, considering the role of macrophages and microglia in neuroinflammatory responses during the progression of AD, we examined the effects of ALA and EDA on neuroinflammation in APP/PS1 mice. The results revealed that administration of ALA and EDA effectively inhibited inflammation responses in the brain tissues of mice, whereas this inhibitory effect was abolished following the knockout of GPR120 or Gαi1 in PAMAs (Supplementary Fig. 14).

### ALA and EDA inhibit amyloid pathology and rescue cognition in AD mice

**Fig. 7:**
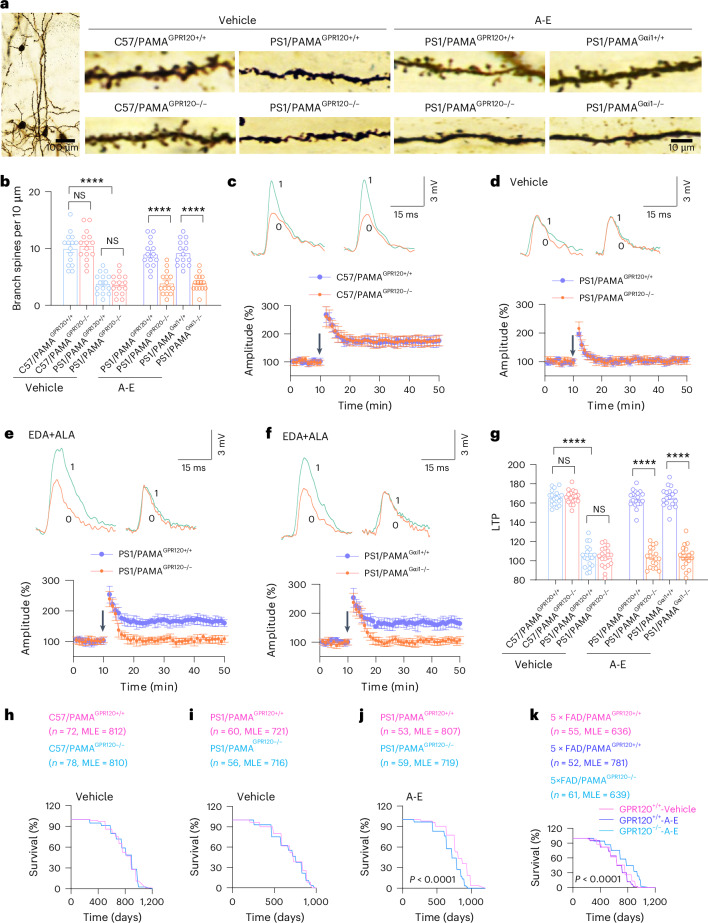
ALA and EDA rescue cognition and restore lifespan of AD mice to normal levels. **a**,**b**, Images (**a**) showing dendritic spines from CA1 pyramidal cells of C57/PAMA^GPR120+/+^, C57/PAMA^GPR120−/−^, PS1/PAMA^GPR120+/+^, PS1/PAMA^GPR120−/−^, PS1/PAMA^Gαi1+/+^ and PS1/PAMA^Gαi1−/−^ mice at 8 months of age. The individual mice at 5 months of age were treated with vehicle or A-E for 45 consecutive days. Experiments were repeated at least three times independently with similar results. **b**, Quantification of dendritic spine densities (the number of spines per 10 μm). Data are presented as mean ± s.e.m., *n* = 15 neurons from 5 mice per group. ****P < 0.0001; NS, no significant difference, one-way ANOVA with Tukey’s multiple comparisons test. **c**–**g**, ALA and EDA restore LTP. EPSPs intracellularly recorded in CA1 pyramidal neurons of the slices from C57/PAMA^GPR120+/+^ and C57/PAMA^GPR120−/−^ (**c**) or PS1/PAMA^GPR120+/+^ and PS1/PAMA^GPR120−/−^ mice (**d**) or PS1/PAMA^Gαi1+/+^ and PS1/PAMA^Gαi1−/−^ mice (**f**) treated with saline vehicle (**c** and **d**) or A-E (**e** and **f**). EPSPs evoked by stimulation of the Schaffer collateral fibers are normalized with respect to 10 min immediately preceding the tetanus (arrow). Representative traces are EPSPs immediately before (0) and 30 min after (1) the tetanus. Averages of the last 10 min of EPSPs were quantified (**g**). Data are presented as mean ± s.e.m., *n* = 18 recordings from 6 mice per group. ****P < 0.0001; NS, no significant difference, one-way ANOVA with Tukey’s multiple comparisons test. **h**–**k**, ALA and EDA restore lifespan of AD mice to normal levels. **h**–**j**, The MLE and survival curves of C57/PAMA^GPR120+/+^ and C57/PAMA^GPR120−/−^ mice (**h**), PS1/PAMA^GPR120+/+^ and PS1/PAMA^GPR120−/−^ mice (**i** and **j**) treated with control vehicle (**h** and **i**) or A-E (**j**). **k**, The MLE and survival curves of 5×FAD/PAMA^GPR120+/+^ (magenta and dark blue) and 5×FAD/PAMA^GPR120−/−^ mice (light blue) treated with control vehicle (magenta) or A-E (dark blue and light blue). *P* < 0.0001, log-rank (Mantel–Cox) test. The exact *P* values are reported as source data. Source data

Integrity of neurons and synapses is critically dependent on brain-resident macrophages and microglial cells that continuously monitor and clear phagocytic targets across the lifespan and contribute a selective synapse loss in the brain of AD^60,61^. Accordingly, we determined the effects of ALA and EDA on synaptic transmission in AD mice. We analyzed postsynaptic spines in the CA1 hippocampus. While we observed synaptic loss in matured synaptic puncta density in AD mice, treatment with ALA and EDA completely restored this synapse loss (Fig. 7a,b). Likewise, we observed that APP/PS1 mice treated with ALA and EDA were normal in the induction of long-term potentiation (LTP; Fig. 7c–g), which has been widely considered as a cellular substrate of learning and memory^62^. This improvement of synaptic function was not observed in either APP/PS1-PAMA^GPR120−/−^ or APP/PS1-PAMA^Gαi1−/−^ mice (Fig. 7g). Together, these results show that activation of GPR120–Gαi1 by ALA and EDA enables the induction of LTP in AD mice.

Having determined that allosteric activation of a cell-type-specific GPR120–Gαi1–mTORC1 in PAMAs with ALA and EDA is therapeutically effective against synaptic loss and memory declines, we next wanted to test whether the same treatment affects the lifespan or life expectancy of mice. We followed two mutant strains of AD mice, including APP/PS1 and 5×FAD with (APP/PS1-PAMA^GRP120−/−^ versus 5×FAD-PAMA^GPR120−/−^ mice) or without (APP/PS1-PAMA^GRP120+/+^ versus 5×FAD-PAMA^GPR120+/+^ mice) deletion of GPR120. In this study, non-AD mice were used as additional controls (C57BL/6-PAMA^GPR120+/+^ and C57BL/6-PAMA^GPR120−/−^ mice). Our data revealed that APP/PS1-PAMA^GPR120+/+^ mice had median life expectancy (MLE) of 721 days, compared to 812 days for C57BL/6-PAMA^GPR120+/+^ mice (Fig. 7h,i), showing that AD mice had shorter lifespan than non-AD control mice. When ALA and EDA were administered, APP/PS1-PAMA^GPR120+/+^ mice displayed a markedly longer lifespan (Fig. 7j), almost the same with the control mice. Administration of ALA and EDA offered similar effects in 5×FAD-PAMA^GPR120+/+^ mice (Fig. 7k). The effects of ALA and EDA on aging processes of AD mice was undetectable in either APP/PS1-PAMA^GPR120−/−^ or 5×FAD-PAMA^GPR120−/−^ mice (Fig. 7i–k). We therefore conclude that allosteric modulation and synergistic activation of a cell-type-specific GPR120 by ALA and EDA not only suppresses the disease progression but also counteracts the negative effects of AD pathology.

## Discussion

**Extended Data Fig. 10:**
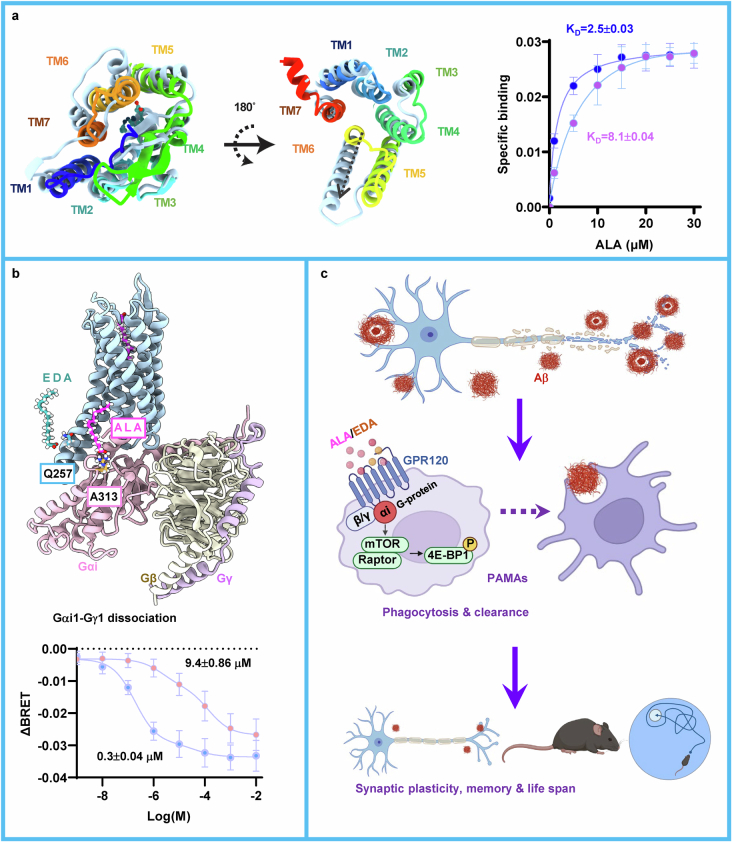
A working model: Allosteric activation of a cell-type specific GPR120 rescues cognition and extends lifespan of Alzheimer’s disease. **a**, EDA binds to GPR120 and causes conformational change, enhancing ALA binding affinity to GPR120. **b**, ALA and EDA allosterically modulate and synergistically activate a cell-type specific GPR120 and GPR120-Gαi1 signaling pathway. **c**, Allosteric activation of a cell-type specific GPR120-Gαi1-mTORC1 in plaque-associated macrophages and activated microglial cells (PAMAs) rescues cognition and extends lifespan in Alzheimer’s disease mice.

In this study, we have shown a cell-type-specific function of GPR120 in the brain, identified allosteric agonists of GPR120 from BRDs, and discovered that allosteric activation of a cell-type-specific GPR120 with ALA and EDA inhibits amyloid pathology, rescues cognition, and restores lifespan to normal levels in mouse preclinical models of AD. Specifically, we have generated the structures of GPR120 bound to ALA with EDA and identified allosteric sites that dictate a cell-type-specific GPR120–Gαi1–mTORC1 activation in PAMAs. In our working model (Extended Data Fig. 10), ALA extends deeply into the binding pocket of GPR120, with a maximal depth of about 12 Å, while EDA penetrates the pocket with a maximum depth of approximately 13 Å. This penetration causes the outward movement of TM6, in turn causes over 5-fold increase of the affinity for ALA-GPR120 binding, and facilitates the opening of an intracellular loop of GPR120 for Gαi1 activation. In addition, ALA in the membrane environment can induce a pronounced separation of TM6, whereas EDA generates forces on both TM6 and TM7, as well as the loop between TM3 and TM4. When both ALA and EDA are present in the membrane at a ratio of 1:10, ALA is engaged with Gαi1 through charge–charge interactions, introducing an anchoring point for Gαi1 protein membrane docking. Concurrently, EDA in the membrane environment causes further displacement of TM6 from TM7, creating additional space for Gαi1 activation. Thus, application of ALA and EDA as allosteric agonists of GPR120 activates a cAMP-independent non-canonical Gαi1 signaling pathway, hence generating the therapeutic effects against amyloid pathology in mouse preclinical models of AD. Together, we concluded that the therapeutic effects of ALA and EDA against amyloid pathology in AD can be achieved by allosteric activation of a cell-type-specific GPR120.

Although the nutritional values of BRDs have been recognized, their clinical translation has been hampered by the impractically high intake required for efficacy and potential risks associated with excessive free fatty acid consumption^63,64^. These limitations reflect broader challenges in PUFA trials for AD, where inconsistent outcomes may stem from the heterogeneity in dosing strategies, treatment duration, and participant demographics. Here, we demonstrate that a low-dose combination of ALA and EDA, but not ALA or EDA alone, is therapeutically effective against Alzheimer’s amyloid pathology. The efficient and sustained enrichment of ALA and EDA in the brain may be attributed to selective uptake and transporter preference, facilitating its incorporation into lipid bilayers and limiting clearance^65^. It could be the underlying mechanism for how administration of ALA and EDA at a relative low dose can make them enriched in brain for a relatively long time.

Free fatty acids produce their physiological functions through engagement with membrane fatty acid receptors^36^, many of which are GPCRs. Over hundreds of GPCRs have been identified^66^. Of these, we have focused our efforts on GPR120, as we have found that it was expressed in PAMAs. To examine whether ALA and EDA may contribute their therapeutic effects through allosteric activation of a cell-type-specific GPR120, we have genetically deleted GPR120 in CTNs or PAMAs and treated these mutant mice with ALA and EDA. Our data have revealed that deletion of GPR120 in PAMAs, but not in CTNs, completely blocks the actions of ALA and EDA in AD mice. These data show that allosteric activation of a cell-type-specific GPR120 in PAMAs inhibits amyloid pathology, rescues cognition, and restores lifespan to normal levels in AD mice.

GPR120 mediates insulin sensitization, stimulates glucagon-like peptide 1 secretion, and controls adipogenesis through coupling to distinct downstream effectors, including the guanine nucleotide–binding (G) proteins Gs, Gi, and β-arrestins1/2 (refs. ^50,67^), but the functions of GPR120 in the brain are yet to be studied. To determine how ALA and EDA synergistically activate GPR120 in PAMAs, we have generated the structures of ALA and EDA bound to GPR120 and demonstrated that synergistic activation of GPR120 occurs via distinct allosteric sites, as well as a cytoplasmic structural rearrangement for selective Gαs versus Gαi1 coupling. EDA-induced conformational change robustly enhances the affinity of ALA binding to GPR120 and enables activation of GPR120–Gαi1–mTORC1. These observations provide an allosteric mechanism for how the therapeutic effects can be achieved by ALA and EDA, but not ALA or EDA alone. Thus, this study can serve as the foundation for the development of precision targeted drugs in the clinical treatment of AD.

Amyloid deposition plays a key role in the disease progression of AD and has been considered as a major contributor to cognitive impairment and synaptic degeneration^55^. Microglia and macrophage, the brain’s innate immune cells, are posited to be the first sensors of local damage and can become dysfunctional with age^68^. Early activation of those immune cells around amyloid plaques is thought to be protective as it limits plaque growth as well as amyloid-related neuronal damage^69^. In this study, we have demonstrated that ALA and EDA activate GPR120 in PAMAs and promote phagocytosis and clearance of amyloid peptides. It should be noted that GPR120 is expressed in activated macrophages and microglial cells within and/or surrounding amyloid plaques of APP/PS1 mice only, but not in these cell types of control non-AD mice. This finding indicates that expression of GPR120 is induced by amyloid aggregates during the disease progression of AD. Hence, this study provides insights into our understanding of how expansion of microglial cells around amyloid plaques inhibits the disease progression of AD during aging.

In addition to amyloid plaques, neurofibrillary tau tangles, as a result of tau protein misfolding, are also described as the pathological hallmarks of AD. In this study, we have primarily focused our efforts on the inhibition of amyloid aggregates by ALA and EDA. Further studies will determine whether and, if yes, how ALA and EDA affect tau protein misfolding by using tau pathological AD mouse models such as rTg4510 mice. Together, these studies will generate promising strategies for therapeutic intervention of the disease progression including amyloid aggregates and tau protein misfolding in the brain of mice with AD.

## Methods

### Animals

All animal experiments were conducted in accordance with the National Institutes of Health Guide for the Care and Use of Laboratory Animals and were approved by the Committee on the Ethics of Animal Experiments of Huazhong University of Science and Technology, Wuhan, China ((2023) Institutional Animal Care and Use Committee number 4136). Mice were housed under a 12 h light–dark cycle (lights on at 8:00 a.m.) at 21 ± 1 °C and 50 ± 5% humidity, with 3–5 mice per cage. Male mice were used to avoid the potential differences between sexes. APP/PS1 (stock number 034832-JAX), 5×FAD (stock number 034840-JAX), CaMKIIα-CreERT2 (stock number 012362), hCD11b-CreERT2 knock-in (stock number 038175), Cg-Rptortm1.1Dmsa/J (stock number 013188), Vglut2-Cre (stock number 028863), and Ai6 (stock number 007906) mice were purchased from the Jackson Laboratory.

GPR120^*loxP*^ mice were generated by inserting *loxP* sites flanking exon 1 into C57BL/6J F1-derived V6.4 embryonic stem cells. After neo cassette excision by flippase recombination enzyme recombination, mice were backcrossed to C57BL/6 for ≥6 generations. GPR120^*loxP*^ mice were crossed with CaMKIIα-CreERT2, or hCD11b-CreERT2 mice and APP/PS1 or 5×FAD mice, to obtain PS1/CTN^GPR120−/−^ or 5×FAD/CTN^GPR120−/−^ and PS1/PAMA^GPR120−/−^ or 5×FAD/PAMA^GPR120−/−^ mice, in which GPR120 was deleted in CTNs or PAMAs upon administration of tamoxifen. Genotyping of transgenic mice was performed by PCR amplification of genomic DNA isolated from tail samples with the following primer sequences: APP (5′-CGACAGTGATCGTCATCACCT-3′, 5′-CTTAGGCAAGAGAAGCAGCTG-3′), PS1 (5′-CAGGTGCTATAAGGTCATCC-3′, 5′-ATCACAGCCAAGATGAGCCA-3′), 5×FAD (5′-AATAGAGAACGGCAGG AGCA-3′, 5′-GCCATGAGGGCACTAATCAT-3′), free fatty acid receptor 4 (5′-AATCCCTCTCCCT AAAGTCACCC-3′, 5′-CCCATGACTGTTTCCTACCCTT-3′), CD11b (5′-ATACC GGAGATCATGCAAGC-3′, 5′-GTAGGTGGAAATTCTAGCATCATCC-3′), CaMK2α-CreERT2 (5′-GACCTGGATGCTGACGAAG-3′, 5′-AGGCAAATTTTGG TGTACGG-3′), and Raptor (5′-CTCAGTAGTGGTATGTGCTCAG-3′, 5′-GGGTACAG TATGTCAGCACAG-3′).

The BRD diet, containing 50% black rice, was provided by the National Key Laboratory of Crop Genetic Improvement at Huazhong Agricultural University and formulated according to the American Institute of Nutrition-1993 Maintenance diet guidelines, with compositional adjustments to maintain equivalent nutrient ratios with the STD diet, as previously described^70^. The comprehensive nutritional profile, including protein, carbohydrate, fat, vitamin, and mineral content, is detailed in Supplementary Table 1. Each mouse was fed 4–5 g of BRD or STD daily. Fresh diet was replenished daily to minimize oxidative degradation, and intake was monitored gravimetrically to confirm consistency in nutrient delivery between BRD and STD groups.

### Cell lines

We purchased HEK 293 cells from the Cell Resource Center of Shanghai Institute for Biological Sciences (Chinese Academy of Sciences).

### Constructs

For the G protein α and γ subunit dissociation assay, the human GPR120 (plasmid 106041) and G protein BRET probes including Gαi1–RLuc8 (plasmid 140973), Gαq1–RLuc8 (plasmid 140982), Gαs1–RLuc8 (plasmid 140980), and Gγ1–YFP (plasmid 42185) were purchased from AddGene. For EDA and ALA binding assays, a Renilla luciferase (RLuc8) tag was fused to the N-terminus of GPR120 (pcDNA3.1–RLuc8–GPR120). For β-arrestin 1/2 recruitment assays, a yellow fluorescent protein (YFP) tag was appended to the C terminus of GPR120. β-arrestin1 (plasmid 36916), β-arrestin2 (plasmid 36917), and pcDNA–RLuc8 (plasmid 87121) were purchased from AddGene. All constructs were verified by DNA sequencing. For the expression of Gαi1 or its constitutively active mutant Gαi1–Q204L, Lenti-Cytomegalovirus Promoter (CMV)–Gαi1–Green Fluorescent Protein (GFP) or Lenti-CMV–Gαi1–Q204L–GFP virus particles (6.5 × 10^13^ genomic particles per ml) were generated.

### Chemicals and reagents

ALA, EDA, flourescein isothiocyanate (FITC)–ALA, and FITC–EDA were purchased from Shanghai Yuanye Bio-Technology. All other chemicals were purchased from Sigma unless mentioned otherwise.

### Behavioral analyses

#### Home cage behaviors

Natural exploratory motivation and repetitive behaviors were assessed in PhenoTyper home cages (40 cm × 40 cm × 40 cm, Noldus) under controlled light (12 h/12 h) and temperature conditions. Mice were individually housed with free access to food and water. After 24 h of habituation, locomotor activity and spontaneous behaviors were recorded for 48 h via an infrared sensor array. Videos were analyzed using EthoVision XT 17 software (Noldus). Body weight and daily food intake (measured gravimetrically) were monitored weekly throughout the study.

#### Morris water maze

Spatial learning and memory were evaluated using a WMT-100 system (Tai Meng Technology) in a circular pool (150 cm diameter) maintained at 25 ± 1 °C. The water was opacified with non-toxic titanium dioxide^23,24^. After 1–2 days of acclimation to the test room, mice underwent 6 days of hidden-platform training (4 trials per day, 90 s per trial). A probe trial was conducted 48 h after the last training session with the platform removed. Time spent in the target quadrant and swim paths were recorded and analyzed. All tests were performed by experimenters blinded to genotype and treatment.

#### Novel arm Y-maze test

Spatial working memory was assessed using a Y-maze with three identical arms (35 cm each) placed at 120° angles, following established protocols^71,72^. Distinct visual cues were placed on the walls of each arm to facilitate spatial discrimination. Three arms were randomly designated as the start arm, novel arm, and other arm. During the first trial (8 min), one arm was blocked. After a 60 min interval, all arms were accessible in a second trial (5 min). Arm entries and time spent in each arm were tracked using EthoVision XT (Noldus). Novel arm preference and total arm entries were analyzed to evaluate working memory and general locomotion, respectively.

#### Medium- and long-chain fatty acids in BRD

STD or BRD feed samples were dissolved in 600 μl extraction reagent (methanol/acetone/water = 65:25:10, including internal standard Lyso PC17:0), using bead-assisted grinding (60 Hz, 2 min) followed by ice-water bath sonication for 20 min. After centrifugation (20,000 *g*, 10 min, 4 °C), 500 μl of the supernatant was collected and dried. The residue was reconstituted in 300 μl of methanol/isopropanol (1:1) containing L-2-chlorophenylalanine as internal standard and centrifuged again, and 200 μl of the supernatant was filtered through a 0.22 μm organic phase membrane. Extracts were analyzed by LC‑MS, with quality control samples prepared from pooled aliquots of all samples.

#### EDA and ALA levels in mouse plasma and brain tissues

Following 45 days of intragastric co-administration of ALA (20 μg g^−1^) and EDA (2 μg g^−1^), plasma and brain homogenates were collected. Fatty acids were methylated by reaction with 1 ml hexane and 1 ml 14% BF₃/MeOH at 100 °C for 1 h. Fatty acid methyl esters were extracted in hexane and analyzed using an HP6890 gas chromatography system equipped with a flame-ionization detector (Agilent Technologies). Peaks were identified by comparison with commercial standards (Nu-Chek Prep), and relative abundances were quantified via GC ChemStation software (C.01.10).

For pharmacokinetic profiling, blood levels of ALA and EDA were measured in C57 and APP/PS1 mice at 0, 1, 2, 4, 8, and 24 h after administration.

#### Protein complexes with ALA and EDA

ALA and EDA from Pub-chem were converted to PDB format using Open Babel v2.3.1 (ref. ^73^), adding hydrogen atoms. The docking process involved ALA with the GPR120/Gαi/Gβ/γ referencing the high-resolution cryo-EM structure of linoleic acid in GPR120/ Gαi/Gβ/γ (PDB 8ID4). EDA was docked to the GPR120/ Gai/Gβ/γ complex based on EPA in GPR120/ Gαi/Gβ/γ (PDB 8ID9). For Gαs, we generated the complex based on spermidine (SPE)-bound mouse trace amine-associated receptor 9 (mTAAR9)–Gαs complex (PDB 8IW4), and for Gαq, docosahexaenoic acid (DHA) bound FFA4 (GPR120)-Gαq complex was used (PDB 8G59).

#### Molecular dynamics simulation

We performed molecular dynamics simulations to meticulously assess the stability of the EDA–GPR120 complex, ensuring a comprehensive representation of the biological system. The molecular dynamics simulations were set up using CHARMM-GUI (v3.7)^74^ membrane builder, allowing for the comprehensive generation of the molecular dynamics input.

In this study, the refined ALA or EDA in complex with GPR120/Gαi/Gβ/γ was strategically positioned within a 154.3 Å × 154.3 Å × 75.17 Å palmitoyl-oleoyl-phosphatidylcholine (POPC) bilayer using the TIP3P model to represent water molecules on both the intracellular and extracellular sides of the membrane. Given the established interactions of GPCRs with cholesterol and anionic lipids in membranes from previous investigations, we conducted an estimation of the insertion of ALA or EDA into the POPC-based bilayer. Although previous research has shown that GPCRs can engage with cholesterol and anionic lipids within the membrane^49,75,76^, we initially opted to use POPC as a model bilayer for its simplicity. Subsequently, contemplating the inclusion of PUFAs into the lipid bilayer composite aimed to create a native-like membrane environment. The specific lipid composition is detailed in Supplementary Tables 3 and 4.

To maintain electrostatic neutrality within the GPR120–EDA complex, 0.15 M NaCl was added using the Monte Carlo method. The force fields used were CHARMM36m for GPR120 and the membrane system, while CHARMM General Force Field (CGenFF) generated the ligand force field parameters^77^. For the construction of the multicomponent membrane systems, we used the CHARMM-GUI membrane builder^78^ for the preparation process. Subsequently, the systems underwent an initial energy minimization phase comprising 20,000 steps using version 2023.3 (ref. ^79^). This was followed by a controlled heating process, gradually raising the temperature from 0 K to 298.15 K (25 °C) in the NVT (maintaining conserving number of particles, volume, and temperature) ensemble over a simulation period exceeding 1,000 ps.

Further simulations were conducted under a constant pressure of 1 atm in the NPT (conserving number particles, pressure, and temperature) for 20–50 ns, using 10.0 kcal mol^−1^ Å^−2^ harmonic restraints. The simulations, including both the NVT and NPT ensembles and the subsequent molecular dynamics production, used a 2 fs time step for 50 ns. The comprehensive analysis of simulation outputs and structural features was carried out using Gromacs (2023.4)^79^, ChimeraX (v1.7)^80^, Pymol (v3.0.1)^81^, and VMD (v1.9.3)^82^.

#### ALA and EDA-GPR120 binding assays

Binding of ALA and EDA to GPR120 was assessed using a BRET assay^83^. HEK 293 cells were cultured in DMEM supplemented with 10% newborn calf serum (B7447, Sigma Aldrich) and transiently transfected with pcDNA3.1–RLuc8–GPR120 using Lipofectamine 2000 (Thermo Fisher). Then 24 h after transfection, cells were seeded into a black 96-well plate (3.5 × 10^4^ cells per well) and further incubated for 24 h.

The saturation binding curve of FITC–EDA and ALA (FITC conjugated labeled at the carboxyl end of EDA or ALA) to RLuc8–GPR120 was determined by treating the transfected cells with FITC–EDA or ALA ranging from 0 to 10 mM in Hanks’ balanced salt solution (HBSS) buffer for 40 min at 37 °C. Coelenterazine 400A at 5 μM (CAS number 70217-82-2, Shanghai Yuanye Bio-Technology) prepared with 1% Pluronic F-127 in Tyrode’s buffer was added 5 min before light emissions were measured using a MithrasLB940 microplate reader (Berthold Technologies) with BRET filter sets. The BRET signal was determined by calculating the ratio of luminescence at 535 nm/485 nm. The binding constants for EDA, ALA, or EDA with ALA were calculated, as previously described^84^.

#### Gα1–Gγ1 dissociation assay

GPR120 was cloned into pcDNA3.1 vector for measurements of signal amplitude and potency of GPR120 activation by EDA and ALA. In brief, HEK 293 cells were co-transfected with GPR120 together with 75 ng Gα–Rluc8 (Gαs1, Gαq1, or Gαi1) and 200 ng YFP–Gγ1 plasmids. After 48 h, cells were treated with ALA, EDA, or their combination for 40 min in Tyrode’s buffer. BRET signals were measured 5 min after adding 5 µM Coelenterazine 400A (CAS number 70217-82-2, Shanghai Yuanye Bio-Technology), and the ratio of emission at 535 nm/485 nm was calculated.

### Measurement of intracellular cAMP

The intracellular cAMP levels were determined in HEK 293 cells transfected with GPR120 and a CRE/RLuc8 reporter. After 4 h stimulation with serially diluted ALA (with or without 10 µM EDA), intracellular cAMP was quantified using the Bright-Glo Luciferase Assay System. Dose–response curves were fitted to a four-parameter logistic model to determine the half-maximal effective concentration (EC_50_) values^85^.

#### Measurement of IP3

The intracellular IP3 levels in HEK 293 cells overexpressing GPR120 in response to EDA or ALA or EDA together with ALA by were determined using IP3 ELISA Kit (ZY-IP3-Ge, Guide-chem) according to the manufacturer’s protocol. In brief, HEK 293 cells expressing GPR120 or empty vector were stimulated with ALA and/or EDA for 20 min. Cells were lysed, and intracellular IP3 levels were measured using a commercial ELISA kit according to the manufacturer’s instructions.

#### β-Arrestin2 recruitment, Gαi1 signaling, and luciferase reporter assay

HEK 293 cells were cultured and transfected with FLAG-tagged β-arrestin2 and YFP-GPR120. Then 24 h after transfection, the cells were distributed into 96-well microplates at a density of 5 × 10^4^ cells per well. After 24 h of incubation at 37 °C, the cells were washed twice with Tyrode’s buffer and stimulated with EDA or ALA or EDA together with ALA at different concentrations for 10 min before cells were collected and subjected to immunoprecipitation and western blotting. Proteins from cell lysates were extracted with radioimmune precipitation buffer in the presence of phosphatase inhibitors and protease inhibitors (Roche Applied Science). Then 20 μg of proteins per lane were separated on a 10% polyacrylamide, precast SDS gel (Bio-Rad) followed by transfer on polyvinylidene difluoride membrane (Immobilon, Millipore), and western blotting was performed with indicated antibodies: anti-GPR120 (sc-390752, Santa Cruz), anti-Gαi1 (A8844, ABclonal), anti-pAKT-T308 (AP0304, ABclonal), anti-AKT1/2 (60203-2-Ig, Proteintech), anti-p4E-BP1 (Thr37/Thr46, ZRB1403, MERCK), anti-mTOR (SAB4501038, Sigma-Aldrich), anti-raptor (A21755, ABclonal), anti-pULK1 (Ser757, 6888, Cell Signaling), anti-pULK1 (D8H5, 8054, Cell Signaling), anti-β-actin (A2228, Sigma-Aldrich), and anti-α-tubulin (66031-1-Ig, Proteintech). For immunoprecipitations, lysates were incubated with 1 μg of anti-FLAG antibody (F7425, Millipore) or YFP antibody (AB1166-100, SICGEN) overnight at 4 °C, and immune complexes precipitated with Protein A/G-conjugated beads (80106 G, Invitrogen). Beads were washed with PBS and resuspended in sample buffer. Lysates and immune complexes were separated by SDS–PAGE and subjected to western blotting.

For reporter assay, HEK 293 cells were co-transfected with β-arrestin2–Rluc8 and YFP–GPR120. After 24 h of incubation at 37 °C, the cells were washed twice with Tyrode’s buffer and stimulated with EDA or ALA or EDA together with ALA at different concentrations for 10 min. Coelenterazine 400A at 5 μM (CAS number 70217-82-2, Shanghai Yuanye Bio-Technology) prepared with 1% Pluronic F-127 in Tyrode’s buffer was added 5 min before light emissions were measured using a Mithras LB 940 Multimode Microplate Reader (Dixie Scientific Serves LLC) with BRET filter sets. The BRET signal was determined by calculating the ratio of luminescence at 530 nm/485 nm.

#### Immunofluorescence

Mice were anesthetized by intraperitoneal injection of pentobarbital and transcardially perfused with PBS followed with 4% paraformaldehyde. Brains were removed and fixed in 4% paraformaldehyde for 24 h at 4 °C, cryoprotected in 15% and 30% sucrose, and sectioned at 30 μm (Thermo Fisher Scientific CryoStar NX50). Immunofluorescence was performed on the brain sections as described previously^23,24,86^. In brief, sections were blocked in 5% bovine serum albumin and 0.3% TritonX-100, then incubated with primary antibodies for 36–48 h at 4 °C. The following antibodies were used: anti-GPR120 (sc-390752, Santa Cruz), anti-GPR120 (AF5219, Affinity Biosciences), anti-CD11b (MA1-10081, M1/70, Invitrogen), anti-Iba1 (019-19741, Wako), anti-Iba1 (sc-32725, Santa Cruz), Anti-NeuN (MAB377, clone A60, Millipore), anti-Aβ_1-16_ (803003, Biolegend), and anti β-actin (A5441, Sigma-Aldrich). Afterward, sections were washed and incubated with species-appropriate Alexa Fluor secondary antibodies (Invitrogen) and counterstained with 4′,6-diamidino-2-phenylindole (DAPI) (D9542, Sigma). For amyloid plaque labeling, sections were washed with PBS and stained with 500 μM thioflavin-S solution (MedChemExpress, HY-D0972), or mice were injected intraperitoneally with 5 mg kg^−1^ Methoxy-X04 (4920, Tocris) 5 h before perfusion.

Fluorescence images were captured using a confocal laser-scanning microscope (Olympus FV3000) and a slide scanner (Olympus SLIDEVIEW VS200). The number of Aβ deposits was measured for each slice using six slices per mouse. Aβ plaque size, which is the total area stained for Aβ counted in a defined region relative to the total area of that region, was calculated by ImageJ (version 1.8.0). For confocal imaging with *z*-stack, a series of images were taken at 0.5 μm intervals through the region of interest, and optical stacks of 10 images (total stack depth, 5 μm) were generated for the figure presentations. The numbers of single or double labeled cells were quantified by sampling every section from the experimental animals^23,24,86^. At least three independent replicates were analyzed per group. Images were analyzed in ImageJ (version 1.8.0) to calculate the number of positive cells per square millimeter.

#### ELISA assays

After consecutive intragastric administration of ALA (20 μg per g body weight) and EDA (2 μg per g body weight) daily for 45 days, cytokine levels (IL-1β, IL-6, TNF) in brain homogenates were quantified using ELISA kit (Nanjing Jiancheng Biotechnology) according to the manufacturers’ instruction until the mice reached the age of 8 months. The absorbance at 450 nm was measured with a microplate reader (BioTek), and the results are presented in picograms per milligram.

For the levels of soluble and in soluble Aβ_1-42_ and Aβ_1-40_ in the brain, lysates were determined by ELISA kits (RE59791, IBL) following the manufacturer’s instructions. In brief, the brain tissues were homogenized in 1× RIPA buffer and centrifuged for 20 min at 20,000 *g* at 4 °C. The supernatants were transferred to a new tube for soluble Aβ42 and Aβ40 (TBS soluble). In addition, the homogenates in 1× Tris-buffer containing 1% Triton X-100 were centrifuged at 20,000 *g* for 40 min at 4 °C, and the supernatant fractions were saved as the Triton X-100 buffer soluble (TBXS) fraction. Then, the TBXS-insoluble pellets were dissolved in 5 M guanidine hydrochloride and formic acid for insoluble Aβ fraction^87,88^. All ELISA measurements were performed in duplicate using standard curves for absolute quantification, with results normalized to total protein concentration. Both biological replicates (*n* = 3 mice per group) and technical replicates were incorporated, clearly distinguished in data analysis.

#### Flow cytometry assay

To characterize Aβ plaques-associated PAMAs, 8-month-old APP/PS1 mice were pre-injected intraperitoneally with methoxy-X04 and perfused 5 h later. Brain tissues were minced and enzymatically dissociated using Papain (5 U ml^−1^, Sigma), DNase I (50 U ml^−1^, Sigma), and Collagenase IV (100 U ml^−1^, Thermo Fisher Scientific) in HBSS (Solarbio) for 30 min at 37 °C. Single-cell suspensions were filtered through a 70 μm cell strainer, separated on a 30% Percoll (Sigma) in ice-cold HBSS, and stained with allophycocyanin (APC)-CD11b (17-0112-82, Invitrogen), FITC–CD14 (11-0149-42, Invitrogen) and phycoerythrin (PE)-CD45 (12-0451-82, Invitrogen) in fluorescence-activated cell sorting buffer for 60 min at 4 °C. Cells were centrifuged and rewashed with fluorescence-activated cell sorting buffer for flow cytometry analysis (SONY, ID7000). Data were analyzed using FlowJo software (v10.4, BD Biosciences).

#### Peripheral monocyte-derived macrophages depletion

To deplete peripheral monocyte-derived macrophages, 4-month-old 5×FAD mice received intraperitoneal injections of 100 µg anti-CD14 antibody (150102, Biolegend, clone M14-23) or isotype control IgG2a (400502, Biolegend, clone RTK2758) for three consecutive days. Subsequently, mice were treated with ALA and EDA or vehicle for 1 month, with concurrent administration of IgG2a or CD14 antibody every 4 days. Following the final treatment, blood samples were collected, subjected to red blood cell lysis, and stained with FITC–CD45 (103108, Biolegend), APC-CD11b (17-0112-82, Invitrogen), and PE-Ly6C (128007, Biolegend) antibodies. Brain tissues were dissociated into single-cell suspensions and stained with APC-CD11b (17-0112-82, Invitrogen) and FITC–CD14 (11-0149-42, Invitrogen) antibodies. Then these cell suspensions were analyzed by flow cytometry. Monocyte-derived macrophages were gated as CD11b⁺Ly6Cʰⁱ events in blood and CD11b⁺CD14⁺ events in the brain. Depletion efficiency was calculated relative to the isotype control group. In addition, Aβ plaque burden was quantified in fixed mouse brain sections by immunofluorescence staining with anti-Aβ_1-16_ (803003, Biolegend) antibody.

#### Primary PAMAs isolation and culture

Primary PAMAs from GPR120^+/+^, GPR120^−/−^, Gαi1^+/+^, Gαi1^−/−^, Raptor^+/+^, and Raptor^−/−^ mice were prepared^89,90^. Briefly, mice at 8–12 weeks of age were anesthetized with pentobarbital and perfused with cold HBSS. The brain cortex tissue was quickly removed, digested into single-cell suspensions, and incubated with FITC–CD11b (11-0112-82, Invitrogen) antibody and propidium iodide (PI, R37169, Invitrogen). Live PAMAs (PI^−^CD11b^+^) were sorted by flow cytometry (SONY, MA900). Then cells were spun down and resuspended in fresh growth medium and seeded in cell culture plates for subsequent assays.

#### RNA isolation and quantitative reverse transcription PCR

Brain primary CD11b-positive cells of C57 and APP/PS1 mice were obtained by flow cytometry as described above. Total RNA was extracted using the TRIzol reagent (Takara, 9109) according to the manufacturer’s protocol. The concentration and purity of RNA were assessed using a spectrophotometer. Subsequently, complementary DNA was synthesized from 1 μg of total RNA per sample using the PrimeScript RT reagent Kit (Takara, RR036A) and following the protocol provided by the manufacturer. Quantitative real-time PCR was used to quantify *P2ry12*, *TREM2*,– *Ly6C*, and *CCR2* messenger RNA expression levels. The relative expression levels were normalized to GAPDH and calculated via the 2^−ΔΔCt^ method. Quantitative PCR primers were as follows: *P2ry12*, forward, 5′-ATGGATATGCCTGGTGTCAACA-3′, reverse, 5′-AGCAATGGGAAGAGAACCTGG-3′; *TREM2*, forward, 5′-CTGGAACCGTCACCATCACTC-3′, reverse, 5′-CGAAACTCGATGACTCCTCGG-3′; *Ly6C*, forward, 5′-GCAGTGCTACGAGTGCTATGG-3′, reverse, 5′-ACTGACGGGTCTTTAGTTTCCTT-3′; *CCR2*, forward, 5′-ATCCACGGCATACTATCAACATC-3′, reverse, 5′-CAAGGCTCACCATCATCGTAG-3′; and *GAPDH*, forward, 5′- AGGTCGGTGTGAACGGATTTG-3′, reverse, 5′- TGTAGACCATGTAGTTGAGGTCA-3′.

#### Aβ phagocytosis and degradation assay in vitro

For Aβ phagocytosis assay, human Aβ_1-42_ peptides were aggregated and labeled by pHrodo Red (P36600, Thermofisher) and diluted to a final concentration of 100 μM. Then 5 × 10^4^ cultured primary PAMAs were seeded overnight into a 12-well plate before adding pHrodo Red labeled Aβ_1-42_ oligomers (Aβ^pH^). After 2 h of treatment, Aβ uptake was determined by quantifying Aβ^pH^ fluorescence, which colocalized with the CD11b markers by immunofluorescence or GFP by transfected Lenti-CMV–Gαi1–IRES-GFP or Lenti-CMV–Gαi1–Q204L–IRES-GFP virus particles. Images in at least three randomized fields per treatment were taken by confocal microscopy, and Image-Pro Plus software 6.0 was used for data analysis.

For Aβ degradation assay, primary PAMAs were seeded in 35 mm confocal dish (biosharp) and incubated with carboxyfluorescein (FAM)-Aβ_1-42_ oligomers (200 nM) for 2 h. Then cells were washed by PBS and cultured in fresh DMEM. The degradation of FAM-Aβ_1-42_ was monitored in real time using a Leica MICA live-cell imaging microscope. Confocal images were acquired and analyzed using ImageJ software. FAM fluorescence intensity per area was quantified.

#### Golgi staining

Following anesthesia with pentobarbital, mice were perfused with saline for blood clearance, and brains were rapidly removed. Golgi staining was performed by using the FD Rapid GolgiStain kit (FD Neuro Technologies, PK401) according to the manufacturer’s instructions. Images of CA1 pyramidal cells were acquired with the microscope (Olympus FV1000). Spine density was quantified using ImageJ (version 1.8.0). Intact dendritic branches were selected for spine density quantification, and all types of spines, including mushroom, thin, and stubby spines, were counted.

#### Electrophysiology

Hippocampal slices (350 μm) were cut from the mice and were placed in a holding chamber for at least 1 h. A single slice was then transferred to the recording chamber and submerged and superfused with artificial cerebrospinal fluid (ACSF, 2 ml min^−1^) that had been saturated with 95% O_2_–5% CO_2_ (refs. ^23,24^). The composition of the ACSF was (in mM): 124 NaCl, 3 KCl, 1.25 NaH_2_PO_4_, 2 MgCl_2_, 2 CaCl_2_, 26 NaHCO_3_, 10 dextrose. Field potential recordings were performed with micropipettes at 30 ± 2 °C filled with ACSF. Excitatory postsynaptic potentials (EPSPs) were intracellularly recorded from CA1 pyramidal neurons in current-clamp mode and evoked by stimulation of the Schaffer-collateral fibers at a frequency of 0.1 Hz. LTP was induced by three trains of 100 Hz stimulation lasting 500 ms, at an intertrain interval of 10 s.

### Statistics and reproducibility

The number of replicates and animals for each experiment are indicated in their respective figure legends and/or method details. No statistical methods were used to pre-determine sample sizes, but our sample sizes are similar to those reported in previous publications^23,24^. Animals were randomly assigned to each group. The investigators were blinded to group allocation during data collection, experimental procedures, and analysis. The data distribution was assumed to be normal, but this was not formally tested. No data were excluded from the analyses. Statistical analyses were performed using GraphPad Prism 9 (GraphPad Software) and SPSS version 16.0 (SPSS). Results are presented as the mean ± standard error of the mean (s.e.m.). For comparisons between two groups, an unpaired Student’s *t*-test was used. Multiple group comparisons were performed using one-way or two-way analysis of variance (ANOVA) followed by multiple comparisons test. Survival analysis was performed using the Kaplan–Meier method, with inter-group comparisons assessed by log-rank (Mantel–Cox) test. Statistical significance was considered at *P < 0.05, **P < 0.01, ***P < 0.001 and ****P < 0.0001.

### Reporting summary

Further information on research design is available in the Nature Portfolio Reporting Summary linked to this article.

## Supplementary information

Supplementary Information Supplementary Figs. 1–14 and Tables 1–4.

Reporting Summary

Supplementary Video 1 ALA induces conformational changes in GPR120, related to Fig. 3.

Supplementary Video 2 EDA induces conformational changes in GPR120, related to Extended Data Fig. 4.

Supplementary Video 3 Changes in GPR120 signaling properties, related to Fig. 4.

## Source data

Source Data Fig. 1 Statistical source data.

Source Data Fig. 2 Statistical source data.

Source Data Fig. 2 Unprocessed western blots.

Source Data Fig. 3 Statistical source data.

Source Data Fig. 4 Statistical source data.

Source Data Fig. 4 Unprocessed western blots.

Source Data Fig. 5 Statistical source data.

Source Data Fig. 6 Statistical source data.

Source Data Fig. 7 Statistical source data.

Source Data Extended Data Fig. 1 Statistical source data.

Source Data Extended Data Fig. 2 Statistical source data.

Source Data Extended Data Fig. 3 Statistical source data.

Source Data Extended Data Fig. 7 Statistical source data.

Source Data Extended Data Fig. 7 Unprocessed western blots.

Source Data Extended Data Fig. 8 Statistical source data.

Source Data Extended Data Fig. 9 Statistical source data.

Source Data Extended Data Fig. 9 Unprocessed western blots.

## Data Availability

Source data are provided with this paper. All other data supporting the findings of this study are available from the corresponding authors on reasonable request.
