# Supplementary material for: Allosteric activation of a cell-type-specific GPR120 inhibits amyloid pathology of Alzheimer’s disease
Source: Nat Aging. 2025 Dec 19;6(1):181–99. doi: 10.1038/s43587-025-01028-4 (PMC12823430; doi:10.1038/s43587-025-01028-4)
Supplement: Supplementary file 1 — Supplementary Figs. 1–14 and Tables 1–4. [file 43587_2025_1028_MOESM1_ESM.pdf]

nature aging

Supplementary information https://doi.org/10.1038/s43587-025-01028-4

Allosteric activation of a cell-type-specific GPR120 inhibits amyloid pathology of Alzheimer's disease

In the format provided by the authors and unedited

Supplementary Fig. 1. Black rice diet produces no effects on social and anxiety-like behaviors.

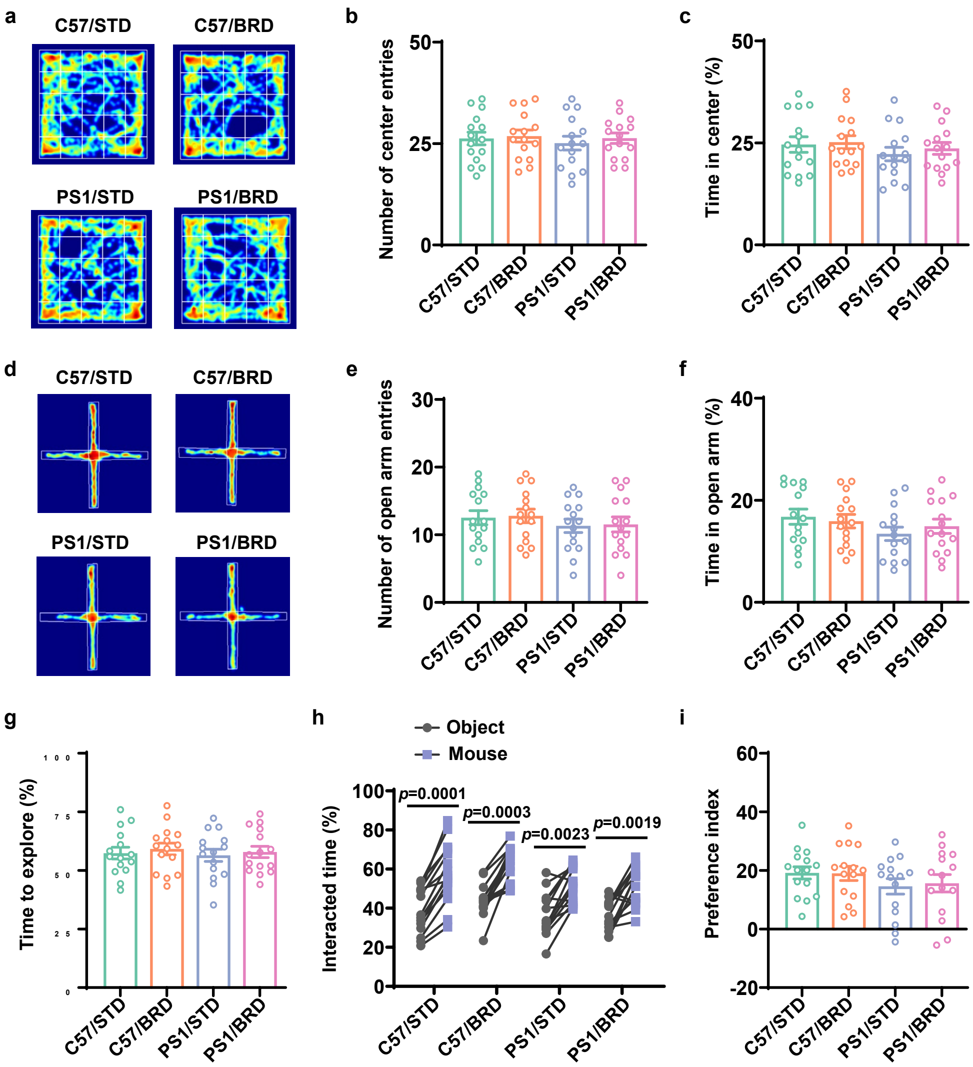

Supplementary Fig. 1. Black rice diet produces no effects on social and anxiety-like behaviors. a-c, Representative heat maps (**a**) and plots (**b** and **c**) showing the number of entrances into the center (**b**) and time spent in the center (**c**) of C57 and PS1 mice administered with STD or BRD in the open field test. **d-f,** Representative heat maps (**d**) and plots (**e** and **f**) showing the number of entrances into the open arm (**e**) and time spent in the open arm (**f**) of C57 and PS1 mice administered with STD or BRD in the elevated plus maze test. **g-i,** Plots showing the percentage of time spent exploration in the chamber (**g**), the percentage of time interaction with an object or a conspecific (**h**) and the preference index of exploration with a conspecific versus an object (**i**) in the novel object recognition test. Data are presented as mean ± SEM, *n*=15 mice per group. One-way ANOVA with Bonferroni's multiple comparisons test was used for **b**, **c**, **e**, **f**, **g** and **i**, and paired Student's t-test for **h**, the exact *p*-values presented in the graphs.

Supplementary Fig. 2. ALA or EDA alone produces no effects on memory declines in APP/PS1mice.

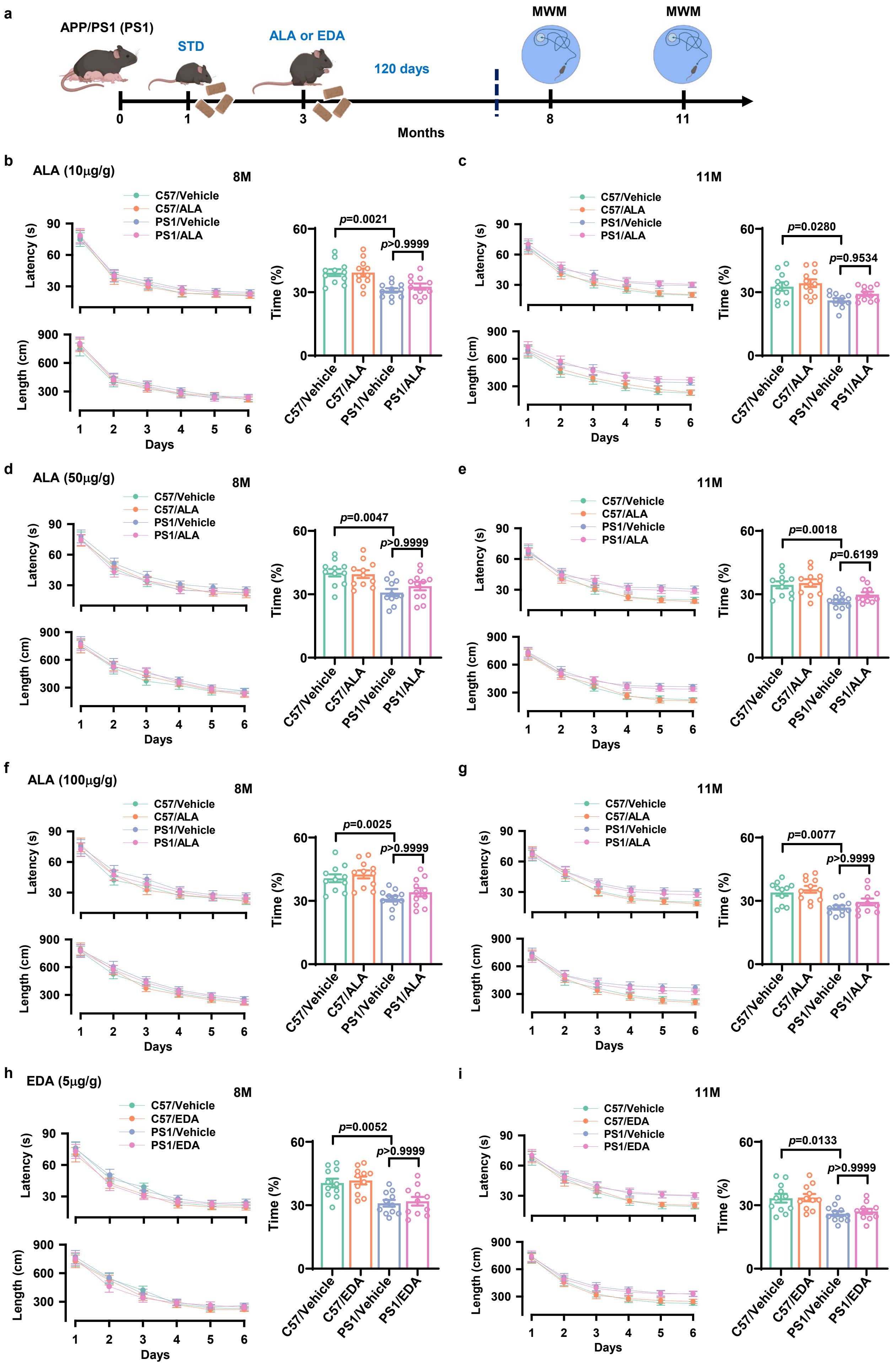

Supplementary Fig. 2. ALA or EDA alone produces no effects on memory declines in APP/PS1 mice. a, Experimental schedules for application of ALA or EDA for 120 consecutive days in 3-month APP/PS1 mice. The Morris water maze (MWM) task was performed when the mice were at 8 or 11 months (M) old of age. **b-g,** Application of ALA alone at 10 (**b** and **c**), 50 (**d** and **e**) or 100 (**f** and **g**) μg/day per g body weight produces no effects on learning and memory in AD mice at 8 (**b, d** and **f**) or 11 (**c, e** and **g**) months of age. The latency and the length of swim path to reach the hidden platform during the training sessions and the representative heat maps and the percentage of time spent in searching of a hidden platform in targeting quadrant (quadrant 2) during the probe trial of the individual C57 and PS1 mice treated with saline vehicle or ALA. **h, i,** Application of EDA alone at 5 μg/day per g body weight produces no effects on learning and memory in AD mice at 8 (**h**) or 11 (**i**) months of age. The latency and the length of swim path to reach the hidden platform during the training sessions and the representative heat maps and the percentage of time spent in searching of a hidden platform in targeting quadrant (quadrant 2) during the probe trial of the individual C57and PS1 mice treated with saline vehicle or EDA. For **b-i**, data are presented as mean ± SEM, *n*=11 mice per group. Two-way ANOVA with Bonferroni's multiple comparisons test was used for analysis of latency and length, one-way ANOVA with Bonferroni's multiple comparisons test was used for analysis of time. The exact *p*-values presented in the graphs.

Supplementary Fig. 3. ALA and EDA levels in mice after administration of ALA and EDA.

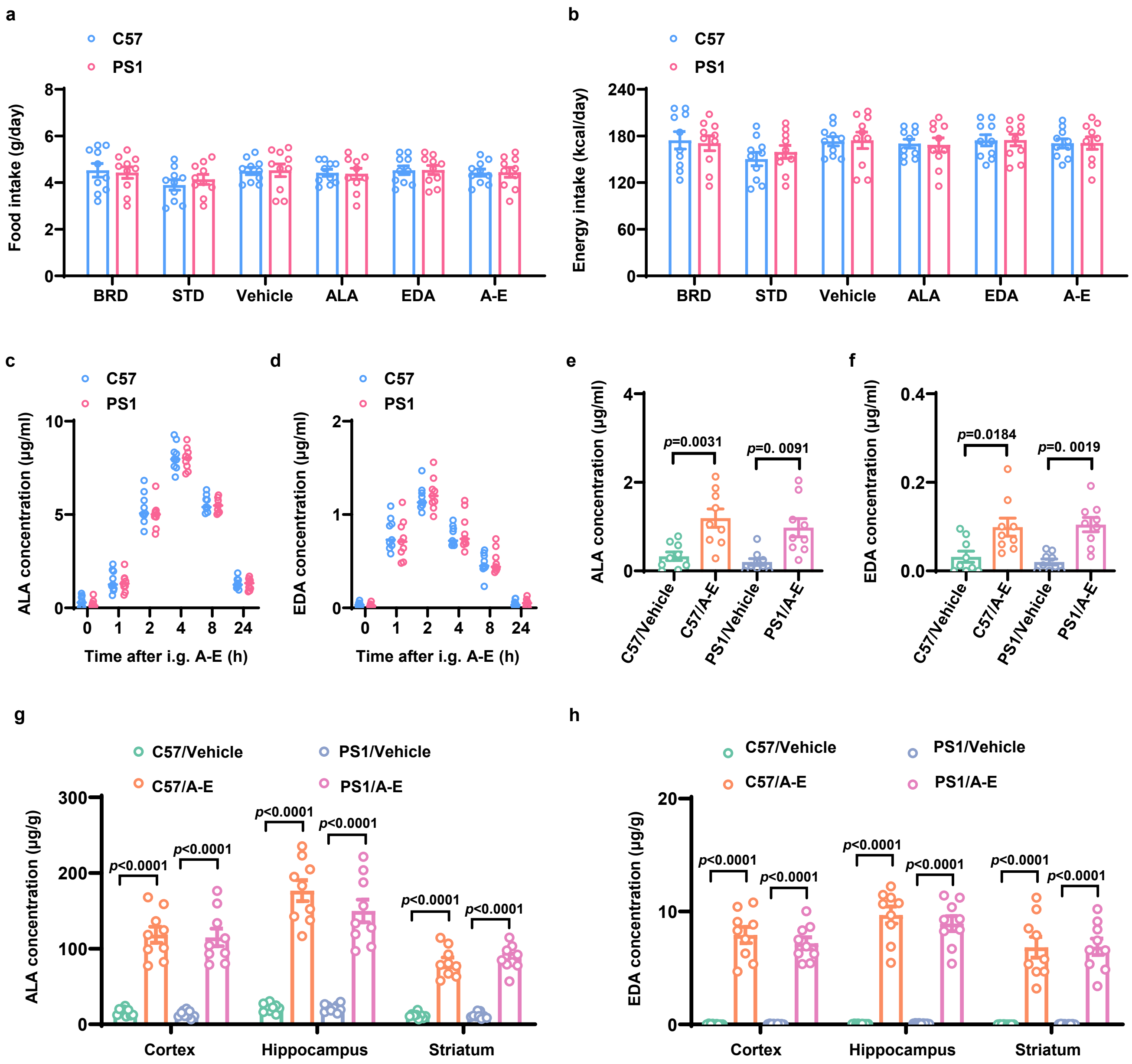

Supplementary Fig. 3. ALA and EDA levels in mice after administration of ALA and EDA. a, b, The average of daily food intake (**a**) and calories (**b**) of C57 and PS1 mice during administration of ALA, or EDA, or ALA with EDA. **c, d,** Time-cross circulating concentrations of ALA (**c**) and EDA (**d**) in C57 and APP/PS1 (PS1) mice after intragastric (i.g.) administration of ALA with EDA (A-E, ALA for 20 μg per g body weight, EDA for 2 μg per g body weight). **e, f,** The circulating concentrations of ALA (**e**) and EDA (**f**) in C57 and APP/PS1 (PS1) mice after administration of saline vehicle or A-E. The mice at 5 months old of age were intragastric administered with control vehicle or A-E for 45 consecutive days, the circulating concentrations of ALA (**e**) and EDA (**f**) were detected immediately. **g, h,** Concentrations of ALA (**g**) and EDA (**h**) in the cortex, hippocampus, and striatum of C57 and PS1 mice after administration of saline vehicle or A-E for 45 consecutive days. For **a**-**h**, data are presented as mean ± SEM from three independent experiments performed in triplicates (*n*=9). One-way ANOVA with Bonferroni's multiple comparisons test was used, the exact *p*-values presented in the graphs.

Supplementary Fig. 4. ALA and EDA levels in brain tissues of 8-month-old age mice.

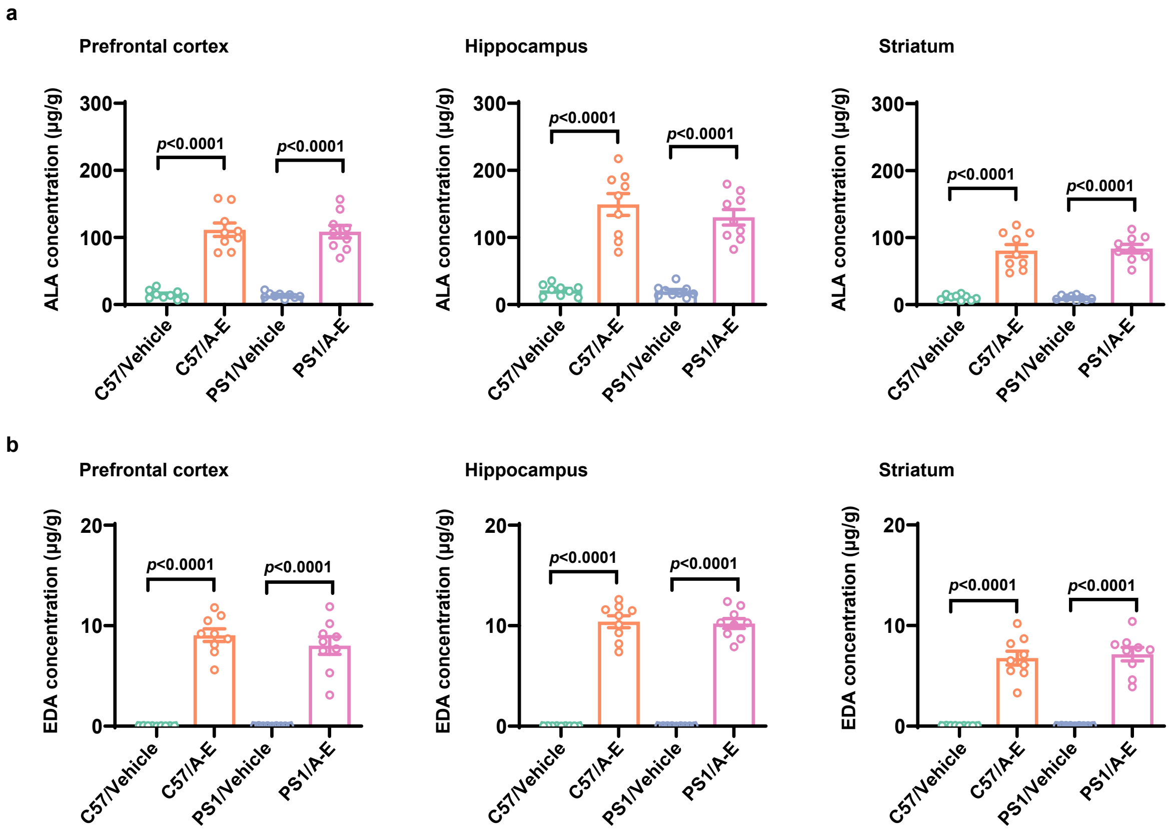

Supplementary Fig. 4. ALA and EDA levels in brain tissues of 8-month-old age mice. a, b, Concentrations of ALA (**a**) and EDA (**b**) in the cortex, hippocampus, and striatum of C57 and PS1 mice after administration of saline vehicle or ALA with EDA (A-E). The mice at 5 months old of age were intragastric administered with control vehicle or A-E (ALA for 20 μg per g body weight, EDA for 2 μg per g body weight) for 45 consecutive days. Brain sections were prepared from the mice at 8 months old of age and the concentrations of ALA (**a**) and EDA (**b**) in the brain tissues were detected. Data are presented as mean ± SEM from three independent experiments performed in triplicates (*n*=9). One-way ANOVA with Bonferroni's multiple comparisons test was used, the exact *p*-values presented in the graphs.

Supplementary Fig. 5. Peripheral monocyte-derived macrophages do not effect Aβ pathology.

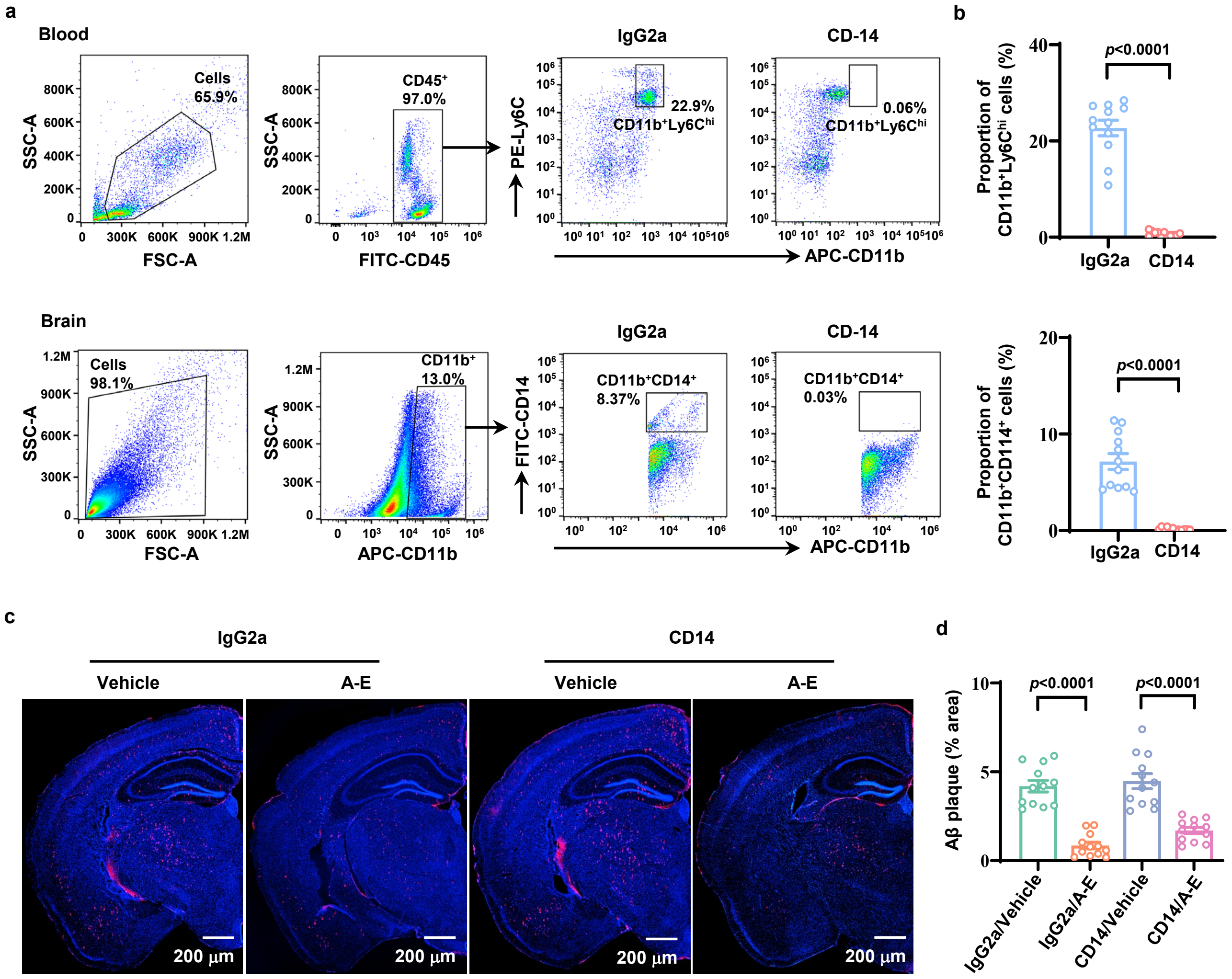

Supplementary Fig. 5. Peripheral monocyte-derived macrophages do not effect Aβ pathology. a, The proportion of CD11b$^+$Ly6C^{hi} peripheral monocytes in blood (top) and CD11b$^+$CD14$^+$ peripheral monocytes-derived macrophages in brain (bottom) of 5×FAD mice after treatment with IgG2a or anti-CD14 antibody. **b,** Plots showing the ratios of CD11b$^+$Ly6C^{hi} and CD11b$^+$CD14$^+$ cells. Data are presented as mean ± SEM, *n*=12 biological replicates from 4 mice per group, t-test. **c, d,** Representative images **(c)** and quantification **(d)** of A$\beta_{1\text{-}16}$-labeled amyloid plaque area throughout brain sections of 5×FAD mice. Mice were treated with IgG2a or anti-CD14 antibody and co-administered saline vehicle or ALA+EDA (A-E). Experiments were repeated at least three times independently with similar results. Data are presented as mean ± SEM, *n*=12 biological replicates from 4 mice per group, one-way ANOVA with Bonferroni's multiple comparisons test was used. The exact *p*-values presented in the graphs.

Supplementary Fig. 6. GPR120 is expressed in cortical neurons.

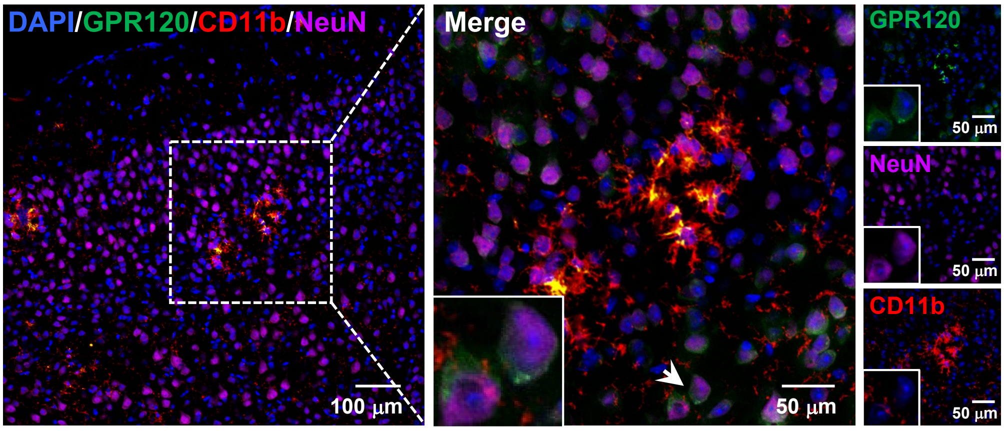

Supplementary Fig. 6. GPR120 is expressed in cortical neurons. Representative images of the APP/PS1 mouse cortex labeled with anti-GPR120 (green), anti-CD11b (red), anti-NeuN (magenta) and DAPI (blue). Images are shown at 20× (left) and 40× (right) magnification. White arrows in the 40× image indicate GPR120 positive neurons (GPR120$^+$/NeuN$^+$ cells). Experiments were repeated at least three times independently with similar results.

Supplementary Fig. 7. Generation of mutant mice by deletion of GPR120 in PAMAs or CTNs of AD mice.

a

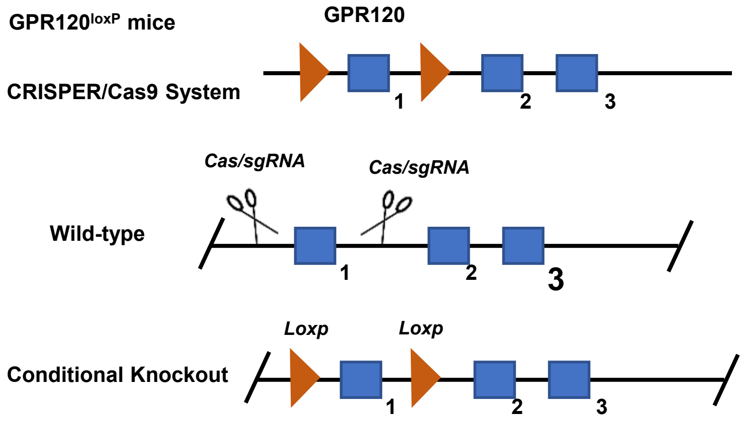

Primer Name	Sequence(5'-3')	PCR size	Primer illustration
JS01216-FFAR4-5wt-tF1	GGGTCTTCTCAGAGCAGGCTC	Fl: 223bp	FFAR4
JS01216-FFAR4-3wt-tR1	GCAGAGCTACTGAAAGGAAGC		
JS11216-FFAR4-wt-tF2	GGGTCTTCTCAGAGCAGGCTC	WT: 418bp	WT

b

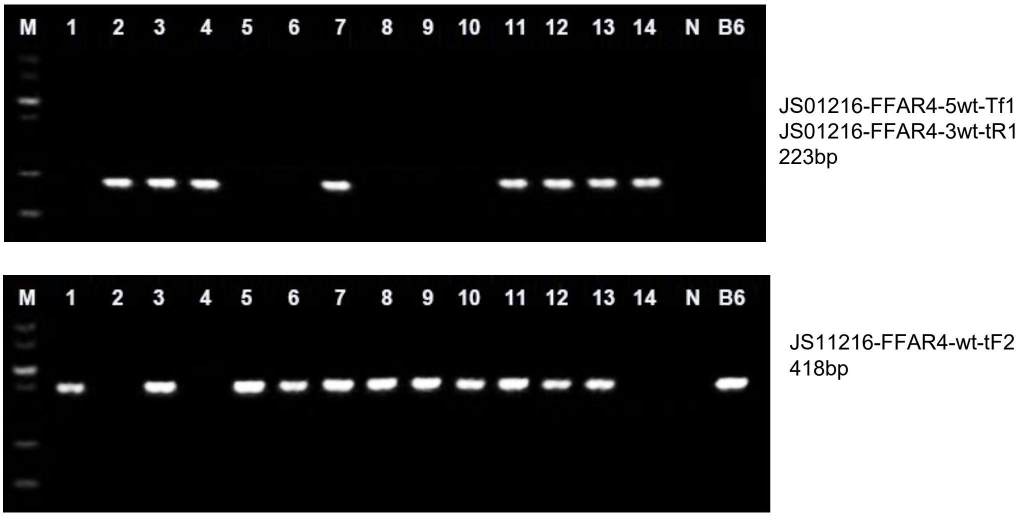

Supplementary Fig. 7. Generation of mutant mice by deletion of GPR120 in PAMAs or CTNs of AD mice. a, Vector design for generation of GPR120^{loxP} mice by inserting a *loxP* site upstream of exon 1 followed by a FRT-flanked neomycin (neo) resistance cassette, followed by *loxP* sites flanking downstream of exon 1 of GPR120 gene. **b,** Representative images showing the genotyping in homozygous (lanes 2, 4, 14), heterozygous (lanes 3, 7, 11, 12, 13) and wildtype (lanes1, 5, 6, 8, 9, 10) control mice from two different litters.

Supplementary Fig. 8. Deletion of GPR120 in PAMAs eliminates the therapeutic effects of ALA and EDA in APP/PS1 mice.

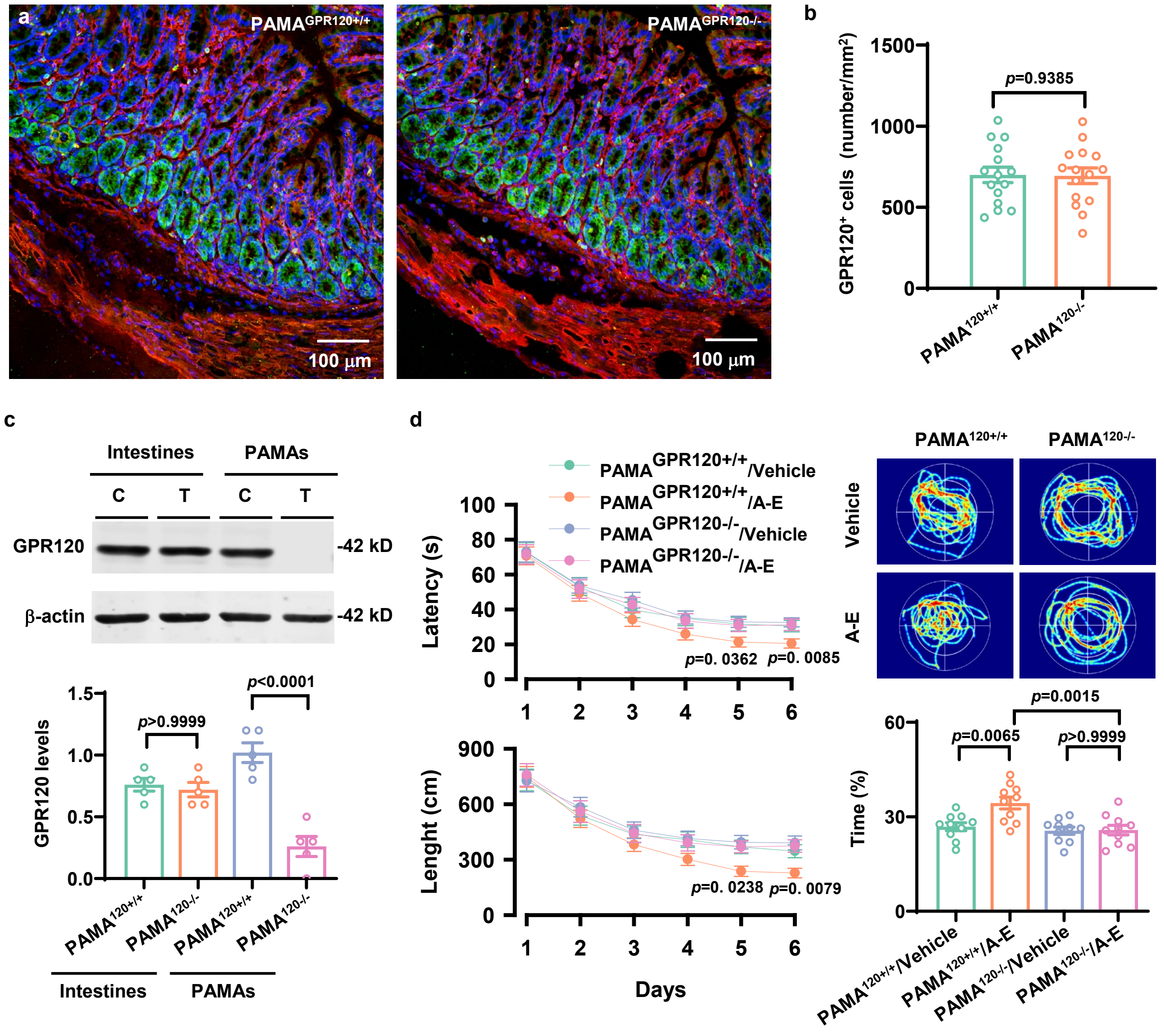

Supplementary Fig. 8. Deletion of GPR120 in PAMAs eliminates the therapeutic effects of ALA and EDA in APP/PS1 mice. a, b, Merged images (**a**) and a plot (**b**) showing the labeling of anti-GPR120 (green) with anti-β-actin (red) in intestines of APP/PS1-PAMA$^{GPR120-/-}$ mice after intraventricular application of tamoxifen. Experiments were repeated at least three times independently with similar results. Data are presented as mean ± SEM, *n*=15 slides from 5 mice per group, t-test. **c,** Representative blots (up) and a plot (down) showing GPR120 in intestinal tissue cells and PAMAs from PPS/PS1 and PS1/GPR120^{CreERT2} mice treated with control vehicle (C) or tamoxifen (T). Data are presented as mean ± SEM, *n*=5 mice per group, t-test. **d,** Deletion of GPR120 in PAMAs by intraventricular application of tamoxifen eliminates the therapeutic effects of A-E. The latency and the length of swim path to reach a hidden platform during the training sessions and the percentage of time spent in searching of a hidden platform in targeting quadrant (quadrant 2) during the probe trial of the individual PS1 mice at 11 months old of age with deletion of GPR120 in PAMAs. The mice were treated with saline vehicle or A-E. Data are presented as mean ± SEM, *n*=10 mice per group. Two-way ANOVA with Bonferroni's multiple comparisons test was used for analysis of latency and length, one-way ANOVA with Bonferroni's multiple comparisons test was used for analysis of time. The exact *p*-values presented in the graphs.

Supplementary Fig. 9. ALA with EDA produce little effects on IP3 and cAMP accumulation.

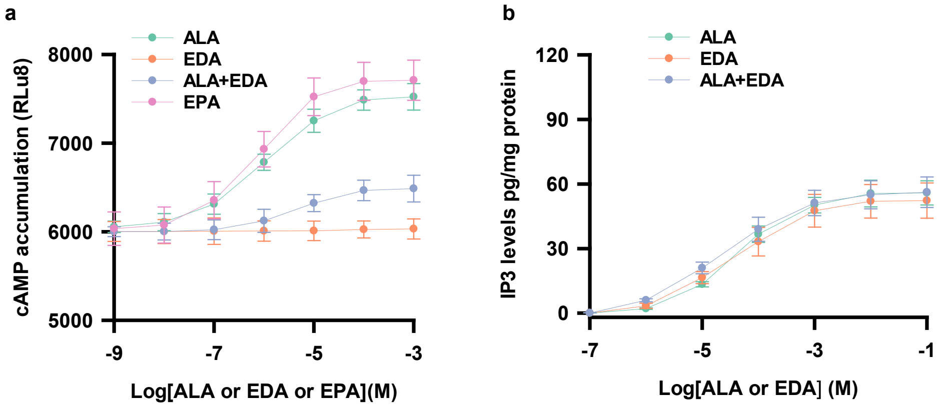

Supplementary Fig. 9. ALA with EDA produce little effects on IP3 and cAMP accumulation. a, b, Concentration-dependent curves of cAMP (**a**) and IP3 accumulations (**b**) in HEK293 cells with GPR120 in response to ALA or EDA or ALA in the absence or in the presence of 10 μM EDA (ALA+EDA). Data are presented as mean ± SEM (*n*=5). EPA, which has been known to stimulate GPR120-Gαs was used as a positive control.

Supplementary Fig. 10. ALA and EDA inhibit amyloid pathology in APP/PS1 mice.

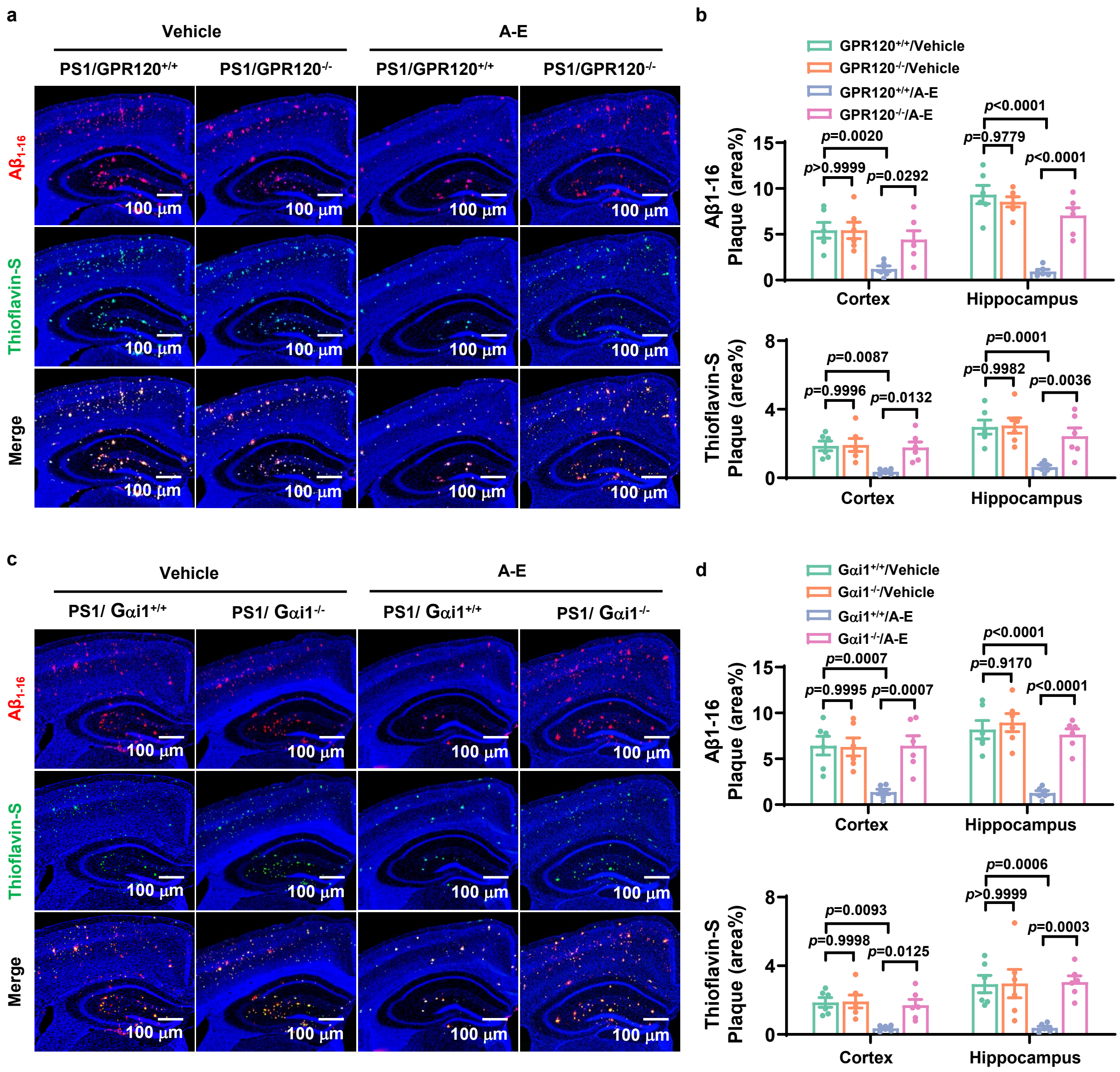

Supplementary Fig. 10. ALA and EDA inhibit amyloid pathology in APP/PS1 mice. Representative images (**a** and **c**) showing brain sections stained with anti-A$\beta_{1\text{-}16}$ antibody (red) and thioflavin-S (green). Plots (**b** and **d**) showing the areas of A$\beta_{1\text{-}16}$-labled thioflavin-S-labeled amyloid plaques in the cortex and hippocampus of PS1/PAMA$^{GPR120+/+}$ and PS1/PAMA$^{GPR120-/-}$ mice (**a** and **b**), or PS1/PAMA$^{G\alpha i1+/+}$ and PS1/PAMA$^{G\alpha i1-/-}$ mice (**c** and **d**) at 8 months old of age. Experiments were repeated at least three times independently with similar results. Data are presented as mean ± SEM, *n*=6 mice per group, one-way ANOVA with Bonferroni's multiple comparisons test was used, the exact *p*-values presented in the graphs.

Supplementary Fig. 11. ALA and EDA lower the levels of soluble and insoluble A$\beta_{1\text{-}42}$.

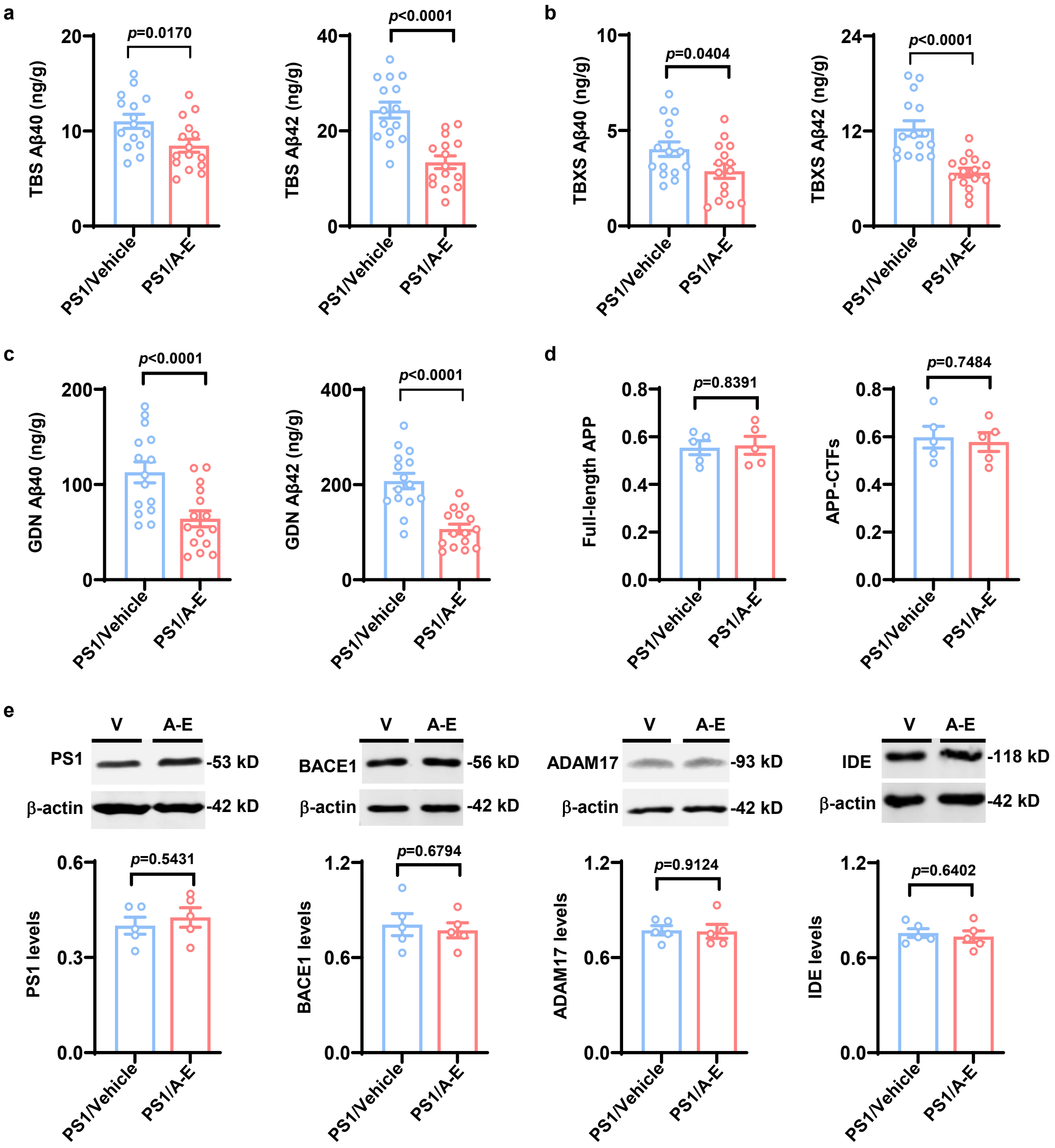

Supplementary Fig. 11. ALA and EDA lower the levels of soluble and insoluble A$\beta_{1\text{-}42}$. a-c. Plots showing TBS soluble (**a**), detergent-soluble (TBSX, **b**) and insoluble (GDN, **c**) A$\beta_{1\text{-}40}$ and A$\beta_{1\text{-}42}$ peptides levels in the cortical tissues from PS1 mice treated with saline vehicle or ALA with EDA (A-E) by ELISA. Data are presented as mean ± SEM, *n*=15 biological replicates from 5 mice per group. **d**, ALA and EDA produce no effects on APP by ELISA. Data are presented as mean ± SEM, *n*=5 mice per group. **e**, Representative blots and plots showing the levels of APP-processing/Aβ-degrading enzymes, including PS1, BACE1, ADAM17, and insulin-degrading enzyme (IDE) in the cortical tissues from PS1 mice treated with vehicle (lane V) or ALA + EDA (A-E), Data are presented as mean ± SEM, *n*=5 mice per group. Unpaired Student's t-test was used for **a**-**e**, the exact *p*-values presented in the graphs.

Supplementary Fig. 12. ALA and EDA accelerate the uptake and clearance of $A\beta_{1-42}$ in PAMAs.

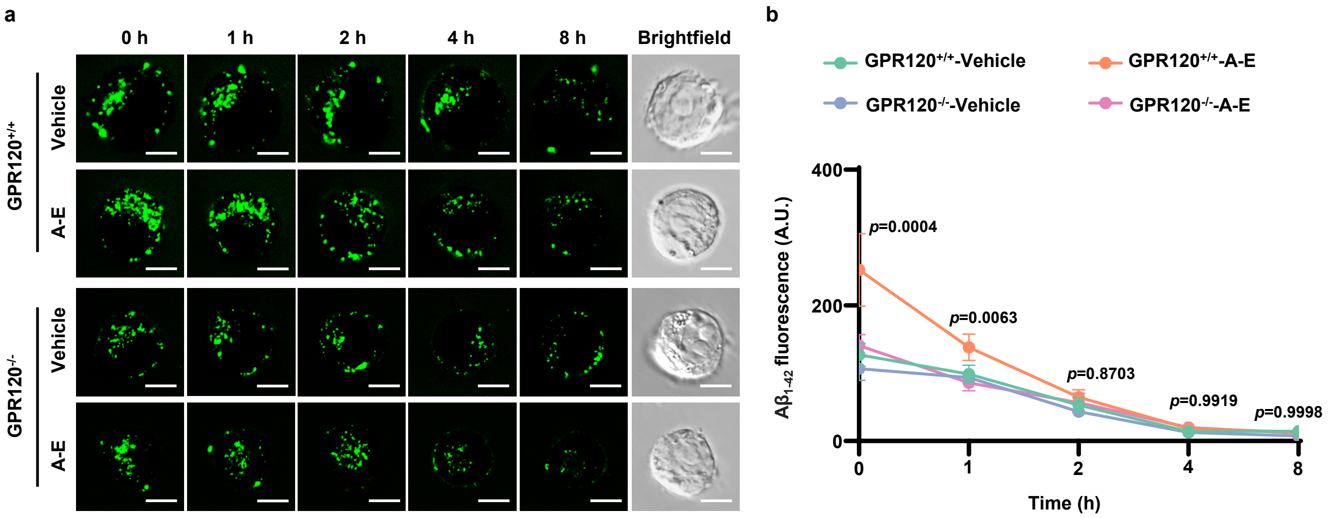

Supplementary Fig. 12. ALA and EDA accelerate the uptake and clearance of $A\beta_{1-42}$ in PAMAs. A, Representative images showing fluorescently labeled $A\beta_{1-42}$ oligomers (FAM-$A\beta_{1-42}$) clearance in cultured PAMAs from $PAMA^{GPR120+/+}$ and $PAMA^{GPR120-}$ mice. Cells were treated with vehicle or ALA with EDA (A-E) for 10 min, and then incubated with FAM-$A\beta_{1-42}$ (1 μM) for 2 hours. Subsequently, the cells were thoroughly washed with DMEM three times and fluorescence images were taken after washed for 0, 1, 2, 4 and 8 hours (h). **b**, Plots showing the rate of FAM-$A\beta_{1-42}$ degradation in PAMAs from $PAMA^{GPR120+/+}$ and $PAMA^{GPR120-/-}$ mice with vehicle or A-E treatment by calculating the fluorescence intensity of each group. Scale bar, 10 μm. Data are presented as mean ± SEM, *n*=3 independent experiments, two-way ANOVA with Tukey's multiple comparisons test was used, the exact *p*-values presented in the graphs.

Supplementary Fig. 13. ALA with EDA do not affect p-ULK1 level in PAMAs.

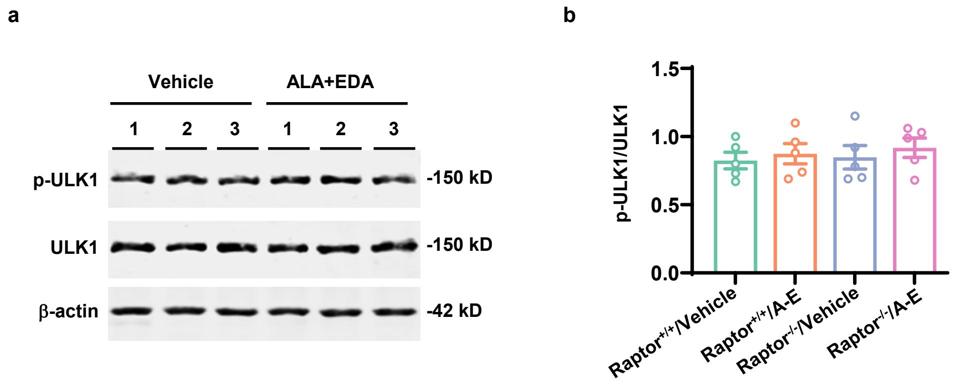

Supplementary Fig. 13. ALA with EDA do not affect p-ULK1 level in PAMAs. Representative blots (**a**) and a plot (**b**) showing the levels of p-ULK1(Ser757) and total ULK1 in PAMAs from PAMAs$^{Raptor+/+}$ and PAMAs$^{Raptor-/-}$ mice treated with vehicle or ALA+EDA. Data are presented as mean ± SEM, *n* = 5 mice per group.

Supplementary Fig. 14. ALA with EDA reduce inflammation in the brain.

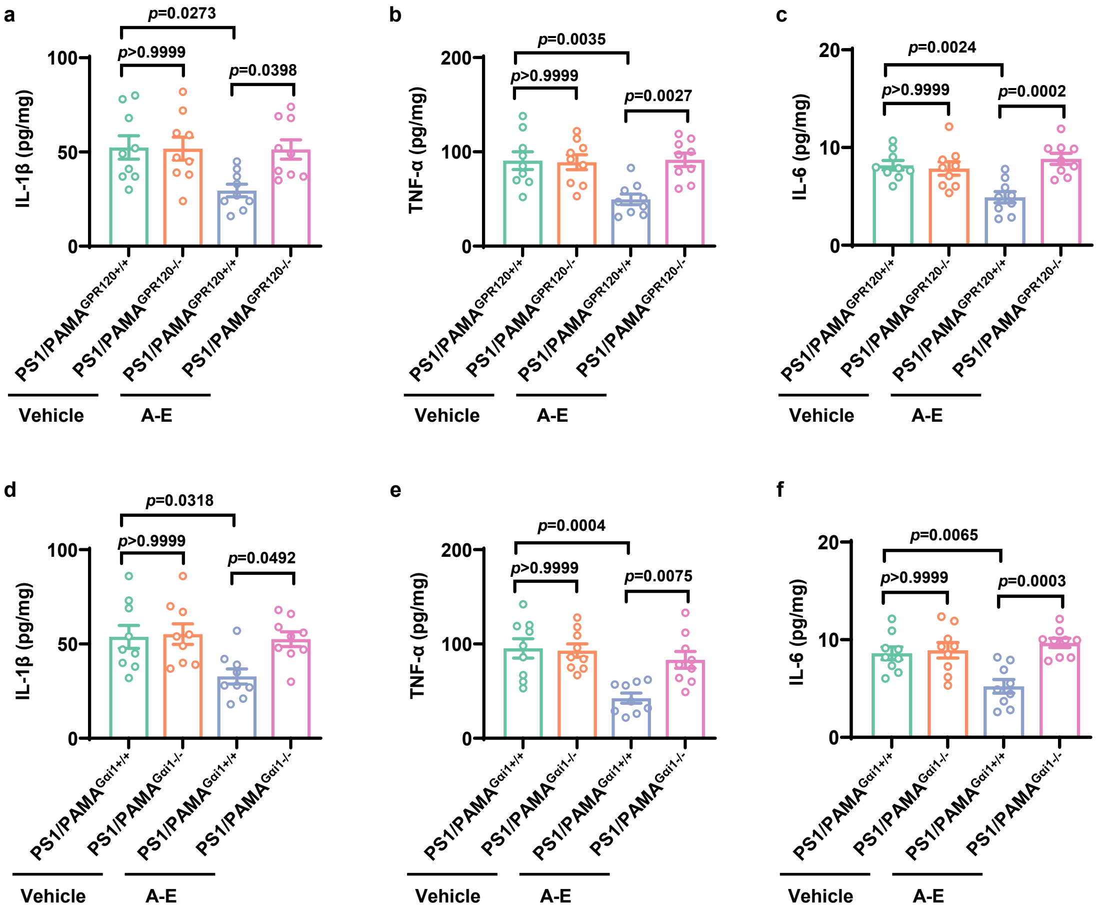

Supplementary Fig. 14. ALA with EDA reduce inflammation in the brain. a-c. Plots showing the levels of inflammatory factors IL-1β (**a** and **d**), TNF-α (**b** and **e**) and IL-6 (**c** and **f**) in the cortical tissues from PS1/PAMA$^{GPR120+/+}$ and PS1/PAMA$^{GPR120-/-}$ (**a-c**), or PS1/PAMA$^{G\alpha i1+/+}$ and PS1/PAMA$^{G\alpha i1-/-}$ (**d-f**) mice treated with vehicle or ALA with EDA (A-E) by ELISA. Data are presented as mean ± SEM from three independent experiments performed in triplicates (*n*=9). One-way ANOVA with Bonferroni's multiple comparisons test was used, the exact *p*-values presented in the graphs.

The original blots related to Supplementary Fig. 8c, Supplementary Fig. 11e and Supplementary Fig. 13a.

Supplementary Fig. 8c:

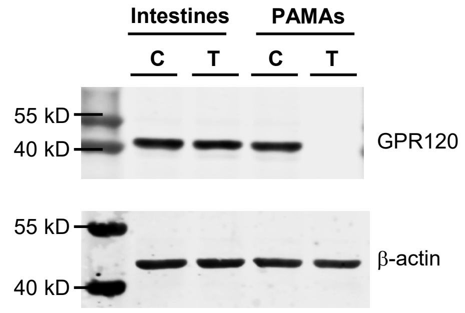

Supplementary Fig. 11e

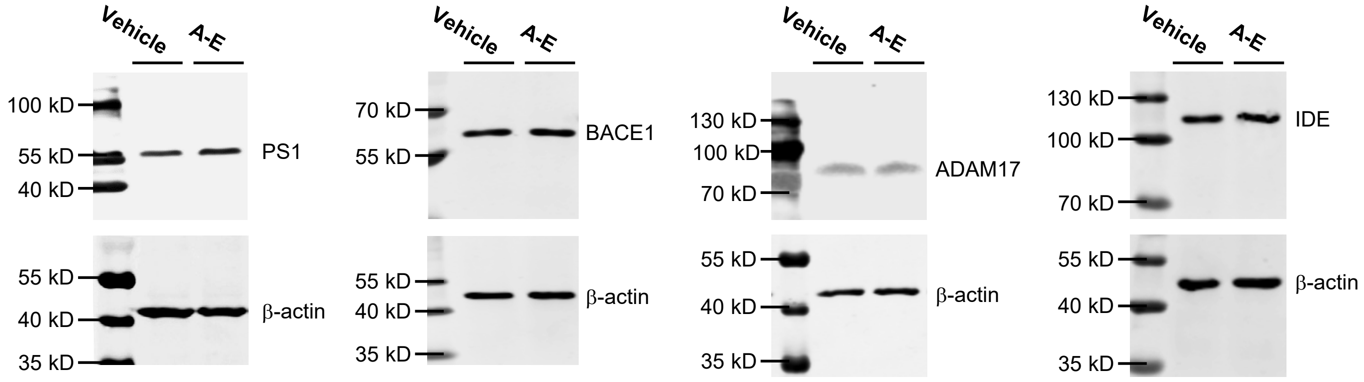

Supplementary Fig. 13a

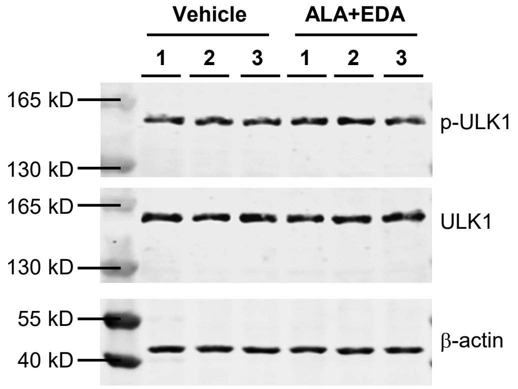

Supplementary table 1. Nutrients in BRD versus STD

Product	STD	BRD
	gm%	gm%
Protein	13	12
Carbohydrate	73	68
Fat	4	4
Total		
kcal/gm	3.8	3.6
Ingredient	gm	gm
Casein	140	87
L-Cystine	1.8	1.8
Black Rice		537
Corn Starch	495.692	108.95
Maltodextrin 10	125	125
Sucrose	100	100
Cellulose, BW200	50	44
Soybean Oil	40	24
t-Butylhydroquinone	0.008	0.014
Mineral Mix S10022M	35	35
Vitamin Mix V10037	10	10
Choline Bitartrate	2.5	1.6
Total	1000	1074.5

Supplementary table 2. The amount of free fatty acids in BRD

Fatty acids	Concentration (ng/g)		FC (BRD/STD)
	BRD	STD	
Decanoic acid	709.73±79.70	997.60±195.21	0.71
Undecanoic acid	0±0	47.18±8.01	0
Dodecanoic acid	1128.14±121.07	1232.06±184.04	0.92
Tridecanoic acid	64.98±9.01	25.78±10.57	2.52
Pentadecanoic acid	3024.72±216.98	397.53±37.83	7.61
Palmitic acid	37207.82±15580.70	39448.59±3339.11	0.94
Margaric acid	6328.39±864.50	256.44±19.90	24.68
Stearic acid	132315.80±9150.01	5286.31±692.39	25.03
Traumatic acid	6.73±1.07	5.94±1.50	1.13
Hexadecenoic acid	8125.43±822.72	417.72±57.15	19.45
Margaroleic acid	449.01±41.19	24.57±3.15	18.28
Oleic acid	4448.94±1151.89	4059.79±197.42	1.10
Elaidic acid	1403.14±217.52	284.49±38.12	4.93
Erucic acid	208.70±15.87	4.5408±2.36	45.96
Eicosadienoic acid	12830.85±1367.11	349.00±39.81	36.76
Linoleic acid	5170.84±1033.30	10453.32±609.20	0.49
Gamma-Linolenic acid	7366.85±827.25	85.417±9.38	86.25
Arachidonic Acid	29.41±4.40	33.18±5.09	0.89
Alpha-Linolenic acid	206682.60±8565.25	26603.67±3797.88	7.77
Eicosapentaenoic acid	44.62±5.22	27.49±3.88	1.62

Supplementary table 2. The amount of free fatty acids in BRD. Free fatty acids in STD and BRD were measured by tandem mass spectrometry (LC-MS/MS) and the ratios between BRD and STD were calculated. Data are mean ± SEM from three independent experiments (*n*=6) performed in triplicates.

Supplementary table 3. List of MD simulations

Sitmulation set	Ligands	Composition of protein	Membrane componant	Sitmulation time(ns)
1	ALA	GPR120	POPC	50
2	ALA	GPR120	POPC and ALA	20
3	ALA	GPR120	POPC and EDA	20
4	ALA	GPR120	POPC, ALA and EDA	20
5	ALA	GPR120	DPPC&DSPC&DOPC&SDPC&SM&CHOL	20
6	EDA	GPR120	POPC	50
7	EDA	GPR120	POPC and ALA	20
8	EDA	GPR120	POPC and EDA	20
9	EDA	GPR120	POPC,ALA and EDA	20
10	EDA	GPR120	DPPC&DSPC&DOPC&SDPC&SM&CHOL	20

Sitmulation set	Ligands	Composition of protein	Membrane componant	Sitmulation time(ns)
1	ALA	GPR120 and Gi heterotrimer	POPC	50
2	ALA	GPR120 and Gq heterotrimer	POPC	50
3	ALA	GPR120 and Gas heterotrimer	POPC	50
4	ALA	GPR120 and Gi heterotrimer	POPC ALA and EDA	20
6	EDA	GPR120 and Gi heterotrimer	POPC	50
7	EDA	GPR120 and Gq heterotrimer	POPC	50
8	EDA	GPR120 and Gas heterotrimer	POPC	50
9	EDA	GPR120 and Gi heterotrimer	POPC ALA and EDA	20

Abbreviations: (9*Z*,12*Z*,15*Z*)-Octadeca-9,12,15-trienoic acid (ALA), (11*E*,14*E*)-icosa-11,14-dienoic acid (EDA), 1,2-dipalmitoyl-sn-glycero-3 phosphocholine (DPPC), 1,2-distearoyl-sn-glycero-3 phosphocholine (DSPC), 1,2-dioleyl-sn-glycero-3 phosphocholine (DOPC), 1-stearoyl-2 docosahexaenoyl-sn-glycero-3-phosphocholine (SDPC) and sphingomyelin (SM), cholesterol (CHOL), and 1-Palmitoyl-2-oleoyl-sn-glycero-3-phosphorylcholine (POPC), respectively.

Supplementary table 4. MD system composition

Component	Number of molecules
POPC	337
GPR120	1
ALA or EDA	1
Hydration number	30
	**Concentration**
NaCl	0.15M

Component	Number of molecules
POPC	326
ALA	11
Total	337
GPR120	1
ALA or EDA	1
Hydration number	30
	**Concentration**
NaCl	0.15M

Component	Number of molecules
POPC	326
EDA	11
Total	337
GPR120	1
ALA or EDA	1
Hydration number	30
	**Concentration**
NaCl	0.15M

Component	Number of molecules
POPC	326
EDA	1
ALIN	10
Total	337
GPR120	1
ALA or EDA	1
Hydration number	30
	**Concentration**
NaCl	0.15M
