# Supplementary material for: Allosteric activation of a cell-type-specific GPR120 inhibits amyloid pathology of Alzheimer’s disease
Source: Nat Aging. 2025 Dec 19;6(1):181–99. doi: 10.1038/s43587-025-01028-4 (PMC12823430; doi:10.1038/s43587-025-01028-4)
Supplement: Supplementary file 2 — Reporting Summary [file 43587_2025_1028_MOESM2_ESM.pdf]

nature portfolio

Corresponding author(s): Youming Lu; Qifa Zhang

Last updated by author(s): Oct 25, 2025

Reporting Summary

Nature Portfolio wishes to improve the reproducibility of the work that we publish. This form provides structure for consistency and transparency in reporting. For further information on Nature Portfolio policies, see our Editorial Policies and the Editorial Policy Checklist.

Statistics

For all statistical analyses, confirm that the following items are present in the figure legend, table legend, main text, or Methods section.

n/a	Confirmed	
☐	☒	The exact sample size (*n*) for each experimental group/condition, given as a discrete number and unit of measurement
☐	☒	A statement on whether measurements were taken from distinct samples or whether the same sample was measured repeatedly
☐	☒	The statistical test(s) used AND whether they are one- or two-sided<br>*Only common tests should be described solely by name; describe more complex techniques in the Methods section.*
☒	☐	A description of all covariates tested
☐	☒	A description of any assumptions or corrections, such as tests of normality and adjustment for multiple comparisons
☐	☒	A full description of the statistical parameters including central tendency (e.g. means) or other basic estimates (e.g. regression coefficient) AND variation (e.g. standard deviation) or associated estimates of uncertainty (e.g. confidence intervals)
☐	☒	For null hypothesis testing, the test statistic (e.g. *F*, *t*, *r*) with confidence intervals, effect sizes, degrees of freedom and *P* value noted<br>*Give P values as exact values whenever suitable.*
☒	☐	For Bayesian analysis, information on the choice of priors and Markov chain Monte Carlo settings
☒	☐	For hierarchical and complex designs, identification of the appropriate level for tests and full reporting of outcomes
☒	☐	Estimates of effect sizes (e.g. Cohen's *d*, Pearson's *r*), indicating how they were calculated

Our web collection on statistics for biologists contains articles on many of the points above.

Software and code

Policy information about availability of computer code

Data collection	Mouse behaviors in home cage, MWM and Y maze were video–recorded using Noldus EthoVision XT (Noldus). LC-MS analysis was conducted by AB Sciex Qtrap 6500+ and AB ExionLC (AB Sciex). The MD simulations were set up using CHARMM-GUI membrane builder, and the analysis of simulation outputs and structural features was carried out utilizing Gromacs, ChimeraX, Pymol and VMD. BRET measurements were acquired using Mithras LB 940 multimode microplate reader (Berthold Technologies, Germany) with the program MikroWin, Version 4.41, or PHERAstar FS with the program PHERAstar control Version 4.00 R4. Images were captured by a Zeiss LSM800 laser scanning confocal microscope or Olympus FV3000 microscope. Electrophysiological recordings were obtained with multiclamp 700B (Axon Instrument, Molecular Devices). ELISA signals were measured on a microtiter plate reader (Thermo Fisher Scientific).
Data analysis	ZEN 3.3 (blue edition), FV31S-SW and ImageJ (NIH, Version 1.8.0) were used to analyze immunofluorescence images. ImageJ (NIH, Version 1.8.0) was used to evaluate the expression of immunoblot images and spine density in Golgi staining. Electrophysiology recordings were preprocessed using Clampfit and Plexon. Statistical analyses were performed with the GraphPad Prism 9.0 software, SPSS 20.0, and Sigma Plot 14.0. Detailed analysis was described in the method sessions.

For manuscripts utilizing custom algorithms or software that are central to the research but not yet described in published literature, software must be made available to editors and reviewers. We strongly encourage code deposition in a community repository (e.g. GitHub). See the Nature Portfolio guidelines for submitting code & software for further information.

Data

Policy information about availability of data

All manuscripts must include a data availability statement. This statement should provide the following information, where applicable:

- Accession codes, unique identifiers, or web links for publicly available datasets
- A description of any restrictions on data availability
- For clinical datasets or third party data, please ensure that the statement adheres to our policy

All data generated in this study are provided within the article and Supplementary Information. Further information regarding to the findings in the Present study are available from the corresponding authors upon request. All data, the mutant lines of mice are available from the authors upon reasonable request.

Research involving human participants, their data, or biological material

Policy information about studies with human participants or human data. See also policy information about sex, gender (identity/presentation), and sexual orientation and race, ethnicity and racism.

Reporting on sex and gender	N/A
Reporting on race, ethnicity, or other socially relevant groupings	N/A
Population characteristics	N/A
Recruitment	N/A
Ethics oversight	N/A

Note that full information on the approval of the study protocol must also be provided in the manuscript.

Field-specific reporting

Please select the one below that is the best fit for your research. If you are not sure, read the appropriate sections before making your selection.

☒ Life sciences ☐ Behavioural & social sciences ☐ Ecological, evolutionary & environmental sciences

For a reference copy of the document with all sections, see nature.com/documents/nr-reporting-summary-flat.pdf

Life sciences study design

All studies must disclose on these points even when the disclosure is negative.

Sample size	No statistical methods were used to predetermine sample sizes. Sample sizes were similar to those reported in the previous publications. All number of mice analyzed are reported in the figure legends.
Data exclusions	No data were excluded from the study.
Replication	The number of independent experiments and replicates are indicated in the corresponding figure legends. Experimental findings were reliably reproduced.
Randomization	Mice were randomly allocated for different experiments based on their genotypes. The results were compared between control (vehicle) and EDA and ALA-treated mice (A-E). For biochemical and immunohistochemical analysis, the sample order were randomized to avoid potential effects from order of samples with certain genotype.
Blinding	The behavior analysis was performed by the examiner who was blind to the genotype or treatment of the animals. In other experiments, all the samples were labelled only with ID numbers and did not indicate treatment groups.

Reporting for specific materials, systems and methods

We require information from authors about some types of materials, experimental systems and methods used in many studies. Here, indicate whether each material, system or method listed is relevant to your study. If you are not sure if a list item applies to your research, read the appropriate section before selecting a response.

Materials & experimental systems

n/a	Involved in the study
☐	☒ Antibodies
☐	☒ Eukaryotic cell lines
☒	☐ Palaeontology and archaeology
☐	☒ Animals and other organisms
☒	☐ Clinical data
☒	☐ Dual use research of concern
☒	☐ Plants

Methods

n/a	Involved in the study
☒	☐ ChIP-seq
☐	☒ Flow cytometry
☒	☐ MRI-based neuroimaging

Antibodies

Antibodies used

Staining:
anti-GPR120 (Santa Cruz, sc-390752, H-10, 1:200), anti-GPR120 (Affinity Biosciences, AF5219, 1:500), anti-Aβ (BioLegend, 803001, 6E10, 1:1000), anti-Iba1 (Wako, 019-19741, 1:500), anti-Iba1 (Santa Cruz, sc-32725, 1:200), anti-CD11b (Invitrogen/ThermoFisher, MA1-10080, M1/70, 1:200), anti-NeuN (Millipore/Merck, MAB377, clone A60, 1:1000), anti-Abeta (Abcam, ab126649, MOAB-2; 1:200), Cell nuclei were stained using 4',6–diamidino–2–phenylindole (DAPI, Sigma-Aldrich/Merck, D9542, 1:1000).
Western blot:
anti-GPR120 (Santa Cruz, sc-390752, H-10, 1:1000), anti-Gαi1 (ABclonal, A8844, 1:1000), anti-pAKT-T308 (ABclonal, AP0304, 1:1000), anti-AKT1/2 (Proteintech, 60203-2-Ig, 1:1000), anti-4E-BP1-Thr37/Thr46 (Merck, ZRB1403, clone 1N7, 1:1000), anti-mTOR (Sigma-Aldrich/Merck, SAB4501038, 1:1000), anti-raptor (A21755, ABclonal, 1:500) , anti-pULK1 (Cell Signaling Technology, 6888, Ser757, 1:1000), anti-pULK1 (Cell Signaling Technology, 8054, D8H5, 1:1000), anti-β-actin (Sigma-Aldrich/Merck, A2228, 1:2000), anti-α-tubulin (Proteintech, 66031-1-Ig, 1:2000), anti-FLAG (Millipore/Merck, F7425, 1:1000), anti-YFP (SICGEN, AB1166-100, 1:1000), anti-PS1 (Proteintech, 16163-1-AP, 1:1000), anti-ADAM17 (Sigma-Aldrich/Merck, AB19027, 1:1000), anti-APP (BioLegend, 800904, LN27, 1:500), anti-IDE (Santa Cruz, sc-393887, F-9, 1:500), anti-BACE1 (Abcam, ab183612, EPR19523, 1:500).
Secondary antibodies:
Alexa Fluor-488, Alexa Fluor-568 or Alexa Fluor-647-conjugated secondary antibodies (Invitrogen/ThermoFisher; A-11008; A-11001; A-11004; A-31573; A-31430; A-21434; A-21432, A-11007) were used.
Flow cytometry:
CD11b-FITC (Invitrogen/ThermoFisher, M1/70, 11-0112-82, 1:100), CD11b-APC (Invitrogen/ThermoFisher, M1/70, 17-0112-82, 1:100), CD14-FITC (Invitrogen/ThermoFisher, 61D3, 11-0149-42, 1:100), CD45-PE (Invitrogen/ThermoFisher, 30-F11, 12-0451-82, 1:100), CD45-FITC (Biolegend, 30-F11, 103108, 1:100), Ly6C-PE (Biolegend, HK1.4, 128007, 1:100)
Monocyte depletion:
anti-CD14 (Biolegend, M14-23, 150102, 100μg), IgG2a (Biolegend, RTK2758, 400502, 100μg)

Validation

1. Validation for immunostaining by the company and/or studies cited on company's websites.
Anti-GPR120 (Santa Cruz, sc-390752, H-10): https://www.scbt.com/zh/p/gpr120-antibody-h-10
Anti-GPR120 (Affinity Biosciences, AF5219): https://www.affbiotech.cn/goods-4526-AF5219-GPR120_Antibody.html
Anti-Aβ (BioLegend, 803001, 6E10): https://www.biolegend.com/en-us/products/purified-anti-beta-amyloid-1-16-antibody-11228
Anti-Iba1 (Wako, 019-19741): https://labchem-wako.fujifilm.com/us/product/detail/W01W0101-1974.html
Anti-Iba1 (Santa Cruz, sc-32725): https://www.scbt.com/zh/p/iba1-antibody-1022-5
Anti-CD11b (Invitrogen/ThermoFisher, MA1-10080, M1/70): https://www.thermofisher.cn/cn/zh/antibody/product/CD11b-Antibody-clone-M1-70-Monoclonal/MA1-10080
Anti-NeuN (Millipore/Merck, MAB377, clone A60): https://www.sigmaaldrich.cn/CN/zh/product/mm/mab377
Anti-Abeta (Abcam, ab126649, MOAB-2): https://www.abcam.cn/products/primary-antibodies/beta-amyloid-antibody-moab-2-ab126649.html
4',6–diamidino–2–phenylindole (DAPI, Sigma-Aldrich/Merck, D9542): https://www.sigmaaldrich.cn/CN/zh/product/sigma/d9542
2. Validation for Western blot by the company and/or studies cited on company's websites.
Anti-GPR120 (Santa Cruz, sc-390752, H-10): https://www.scbt.com/zh/p/gpr120-antibody-h-10
Anti-Gαi1 (ABclonal, A8844): https://abclonal.com.cn/catalog/A8844
Anti-pAKT-T308 (ABclonal, AP0304): https://abclonal.com.cn/catalog/AP0304
Anti-AKT1/2 (Proteintech, 60203-2-Ig): https://www.ptgcn.com/products/AKT-Antibody-60203-2-Ig.htm
Anti-4E-BP1-Thr37/Thr46 (Merck, ZRB1403, clone 1N7): https://www.sigmaaldrich.cn/CN/zh/product/sigma/zrb1403
Anti-mTOR (Sigma-Aldrich/Merck, SAB4501038): https://www.sigmaaldrich.cn/CN/zh/product/sigma/sab4501038
Anti-raptor (ABclonal, A21755): https://abclonal.com.cn/catalog/A21755
Anti-pULK1 (Cell Signaling Technology, 6888, Ser757): https://www.cellsignal.cn/products/primary-antibodies/phospho-ulk1-ser757-antibody/6888
Anti-pULK1 (Cell Signaling Technology, 8054, D8H5): https://www.cellsignal.cn/products/primary-antibodies/ulk1-d8h5-rabbit-mab/8054
Anti-β-actin (Sigma-Aldrich/Merck, A2228): https://www.sigmaaldrich.cn/CN/zh/product/sigma/a2228
Anti-α-tubulin (Proteintech, 66031-1-Ig): https://www.ptgcn.com/products/tubulin-Alpha-Antibody-66031-1-Ig.htm
Anti-FLAG (Millipore/Merck, F7425): https://www.sigmaaldrich.cn/CN/zh/product/sigma/f7425
Anti-YFP (SICGEN, AB1166-100): https://www.zhscience.com/Uploads/file/20210420/AB1166-100.pdf
Anti-PS1 (Proteintech, 16163-1-AP): https://www.ptgcn.com/products/PSEN1-Specific-Antibody-16163-1-AP.htm
Anti-ADAM17 (Sigma-Aldrich/Merck, AB19027): https://www.sigmaaldrich.cn/CN/zh/product/mm/ab19027
Anti-APP (BioLegend, 800904, LN27): https://www.biolegend.com/en-us/products/purified-anti-app-antibody-10901
Anti-IDE (Santa Cruz, sc-393887, F-9): https://www.scbt.com/zh/p/ide-antibody-f-9
Anti-BACE1 (Abcam, ab183612, EPR19523): https://www.abcam.cn/products/primary-antibodies/bace1-antibody-epr19523-ab183612.html
3. Validation for Flow cytometry by the company and/or studies cited on company's websites.

CD11b-FITC (Invitrogen/ThermoFisher, M1/70, 11-0112-82) :https://www.thermofisher.cn/cn/zh/antibody/product/CD11b-Antibody-clone-M1-70-Monoclonal/11-0112-82
CD11b-APC (Invitrogen/ThermoFisher, M1/70, 17-0112-82) :https://www.thermofisher.cn/cn/zh/antibody/product/CD11b-Antibody-clone-M1-70-Monoclonal/17-0112-82
CD14-FITC (Invitrogen/ThermoFisher, 61D3, 11-0149-42) :https://www.thermofisher.cn/cn/zh/antibody/product/CD14-Antibody-clone-61D3-Monoclonal/11-0149-42
CD45-PE (Invitrogen/ThermoFisher, 30-F11, 12-0451-82) :https://www.thermofisher.cn/cn/zh/antibody/product/CD45-Antibody-clone-30-F11-Monoclonal/12-0451-82
CD45-FITC (Biolegend, 30-F11, 103108, 1:100):https://www.biolegend.com/en-us/products/fitc-anti-mouse-cd45-antibody-99
Ly6C-PE (Biolegend, HK1.4, 128007, 1:100):https://www.biolegend.com/en-us/products/pe-anti-mouse-ly-6c-antibody-4904
3. Validation for Monocyte depletion by the company and/or studies cited on company's websites:
anti-CD14 (Biolegend, M14-23, 150102, 100μg):https://www.biolegend.com/en-us/products/purified-anti-mouse-cd14-antibody-11919
IgG2a (Biolegend, RTK2758, 400502, 100μg):https://www.biolegend.com/en-us/products/purified-rat-igg2a-kappa-isotype-ctrl-1845

Eukaryotic cell lines

Policy information about cell lines and Sex and Gender in Research

Cell line source(s)	HEK293T cell line was purchased from ATCC.
Authentication	HEK293T cells were obtained from ATCC and used without further authentication.
Mycoplasma contamination	All cell lines tested negative for mycoplasma.
Commonly misidentified lines (See ICLAC register)	No commonly misidentified cell lines were used in this study.

Animals and other research organisms

Policy information about studies involving animals; ARRIVE guidelines recommended for reporting animal research, and Sex and Gender in Research

Laboratory animals	APP/PS1 (Stock No: 034832-JAX), 5xFAD (Stock No: 034840-JAX), CaMKIIα-CreERT2 (Stock No: 012362), hCD11b-CreERT2 knock-in (Stock No: 038175), Cg-Rptortm1.1Dmsa/J (Stock No: 013188), Vglut2-Cre (Stock No: 028863) and Ai6 (Stock No: 007906) mice were purchased from the Jackson Laboratory (Ellsworth, ME, USA). FFAR4loxp (GPR120loxp) mice were generated and obtained from Shanghai Model Organisms Center (Shanghai, China). All transgenic lines were backcrossed to the C57BL/6J. Mice were bred and reared under the same conditions, and housed in groups of three to five mice/cage under a 12-h light-dark cycle, with lights on at 8:00 am, at a consistent ambient temperature (21±1°C) and humidity (50±5%). All the mice used in this study were 2 to 12-month-old.
Wild animals	No wild animals were used in this study
Reporting on sex	Only male mice were included in behavioral tests. In other experiments, both male and female mice were included.
Field-collected samples	No wild animals were used in this study
Ethics oversight	Animal studies were approved by the Committee on the Ethics of Animal Experiments of Huazhong University of Science and Technology, Wuhan, China ([2023] IACUC Number: 4136).

Note that full information on the approval of the study protocol must also be provided in the manuscript.

Plants

Seed stocks	N/A
Novel plant genotypes	N/A
Authentication	N/A

Flow Cytometry

Plots

Confirm that:

☒ The axis labels state the marker and fluorochrome used (e.g. CD4-FITC).

☒ The axis scales are clearly visible. Include numbers along axes only for bottom left plot of group (a 'group' is an analysis of identical markers).

☒ All plots are contour plots with outliers or pseudocolor plots.

☒ A numerical value for number of cells or percentage (with statistics) is provided.

Methodology

Sample preparation

Single-cell suspensions of the brain cortex tissues of GPR120+/+, GPR120-/-, Gαi1+/+, Gαi1-/-, Raptor+/+, or Raptor-/- mice were prepared, and then directly stained with FITC-CD11b (Invitrogen/ThermoFisher, MA1-10081) antibody and Propidium Iodide (PI, Invitrogen/ThermoFisher, R37169). Live brain macrophages (PI-CD11b+) were sorted by flow cytometry (SONY, MA900). For measurement of cell types of Aβ plaques-associated PAMAs, 8-months APP/PS1 mice were pre-injected intraperitoneally with methoxy-X04. 5 hours after injection, mice were given anesthesia and perfused with ice-cold HBSS and isolated brain quickly. The brain tissues were minced into pieces, dissociated enzymatically in Papain (5 U/mL, Sigma), DNase I (50 U/mL, Sigma), and Collagenase IV (100 U/mL, Thermo Fisher Scientific) in HBSS (Solarbio) for 30 min at 37°C. Homogenize the dissociated tissues by pipetting gently up and down. The homogenates were filtered through a 70-µm cell strainer and centrifuged at 550g for 5 min. Then, cells were resuspended in 30% Percoll (Sigma) in ice-cold HBSS and centrifuged at 800 × g at 4°C for 15 min.
For measurement of the depletion efficiency of peripheral monocyte-derived macrophages, 4-month-old 5×FAD mice received intraperitoneal injections of 100 µg anti-CD14 antibody (150102, Biolegend, clone M14-23) or isotype control IgG2a (400502, Biolegend, clone RTK2758) for 3 consecutive days. Subsequently, mice were administrated with ALA and EDA or vehicle for one month, with concurrent administration of IgG2a or CD14 antibody every 4 days. Following the final treatment, blood samples were collected, subjected to RBC lysis, and brain tissues were dissociated into single-cell suspensions.

Instrument

Flow cytometry assay was performed using the SONY MA900 Flow Cytometer with the fluorescence channel voltages adjusted using an unstained sample. For measurement of cell types of Aβ plaques-associated PAMAs, cell pellets were incubated with APC-CD11b (17-0112-82, Invitrogen), FITC-CD14 (11-0149-42, Invitrogen) and PE-CD45 (12-0451-82, Invitrogen). For measurement of the depletion efficiency of peripheral monocyte-derived macrophages, blood samples were stained with FITC-CD45 (103108, Biolegend), APC-CD11b (17-0112-82, Invitrogen) and PE-Ly6C (128007, Biolegend) antibodies, and brain samples were stained with APC-CD11b (17-0112-82, Invitrogen) and FITC-CD14 (11-0149-42, Invitrogen) antibodies. Flow cytometry assay was performed using SONY ID7000 Flow Cytometer.

Software

Data analysis was performed using FlowJo (V10) analytical software

Cell population abundance

PAMAs were gated for FITC-CD11b expression. Aβ plaques-associated macrophage were gated for methoxy-X04, APC-CD11b, and PE-CD45 expression. Aβ plaques-associated peripheral monocytes were gated for methoxy-X04, APC-CD11b, and FITC-CD14 expression. Monocyte-derived macrophages were gated as $CD11b^{+}Ly6C^{hi}$ events in blood and $CD11b^{+}CD14^{+}$ events in the brain.

Gating strategy

FSC-A and SSC-A were used to identify the starting cell population (population 1, cells). PI were applied to exclude dead cells and CD11b were applied to gate PAMAs. FSC-A and methoxy-X04 were used to identify the Aβ plaques-associated cells. CD11b were applied to gate PAMAs. CD14 were used to identify peripheral monocytes and CD45 for macrophages.

☒ Tick this box to confirm that a figure exemplifying the gating strategy is provided in the Supplementary Information.
