# Supplementary figures and images for: Allosteric activation of a cell-type-specific GPR120 inhibits amyloid pathology of Alzheimer’s disease
Source: Nat Aging. 2025 Dec 19;6(1):181–99. doi: 10.1038/s43587-025-01028-4 (PMC12823430; doi:10.1038/s43587-025-01028-4)

Figure 2d:

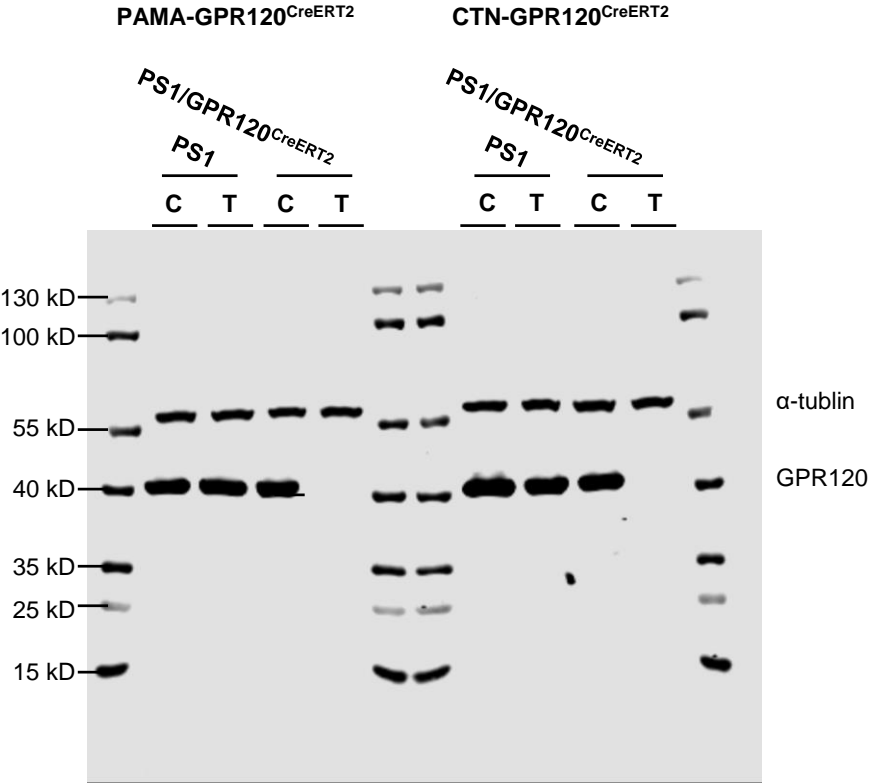

Supplement: Supplementary file 8 — Unprocessed western blots. [file 43587_2025_1028_MOESM8_ESM.pdf]

Fig. 4j:

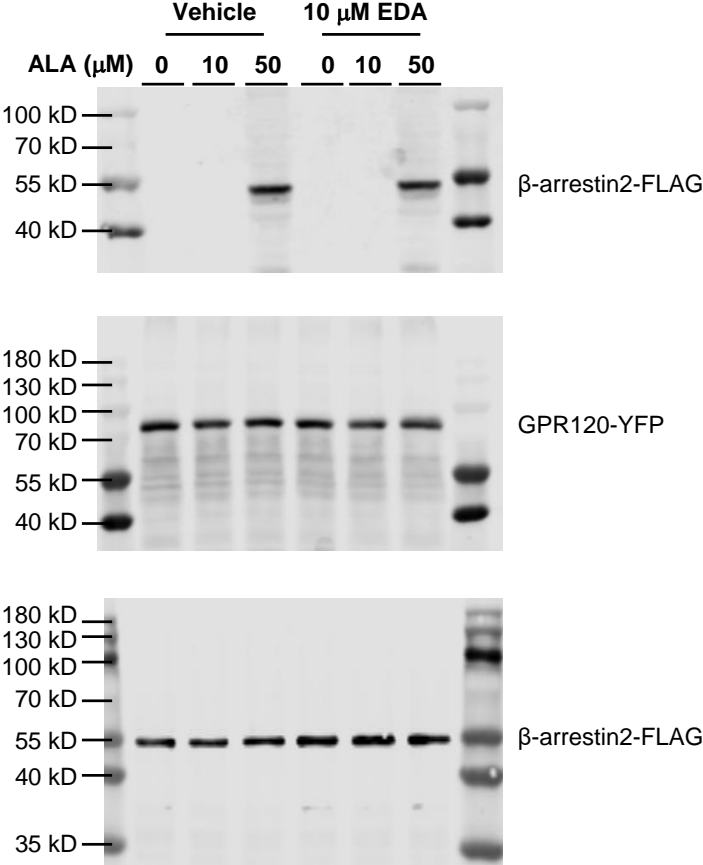

Supplement: Supplementary file 11 — Unprocessed western blots. [file 43587_2025_1028_MOESM11_ESM.pdf]

Extended Data Fig. 7

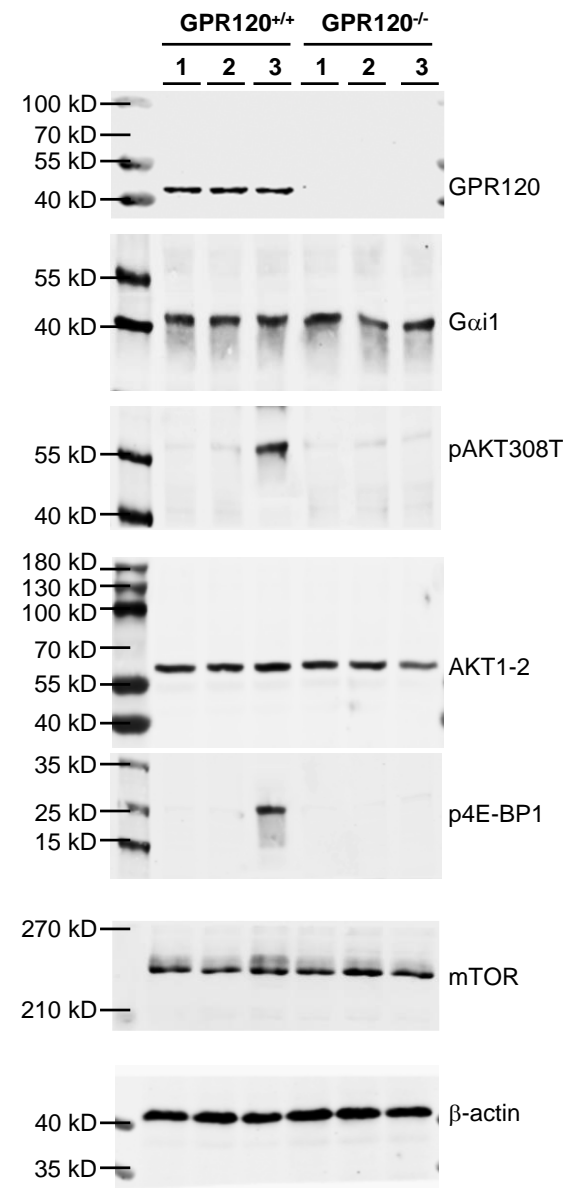

Extended Data Fig. 7b:

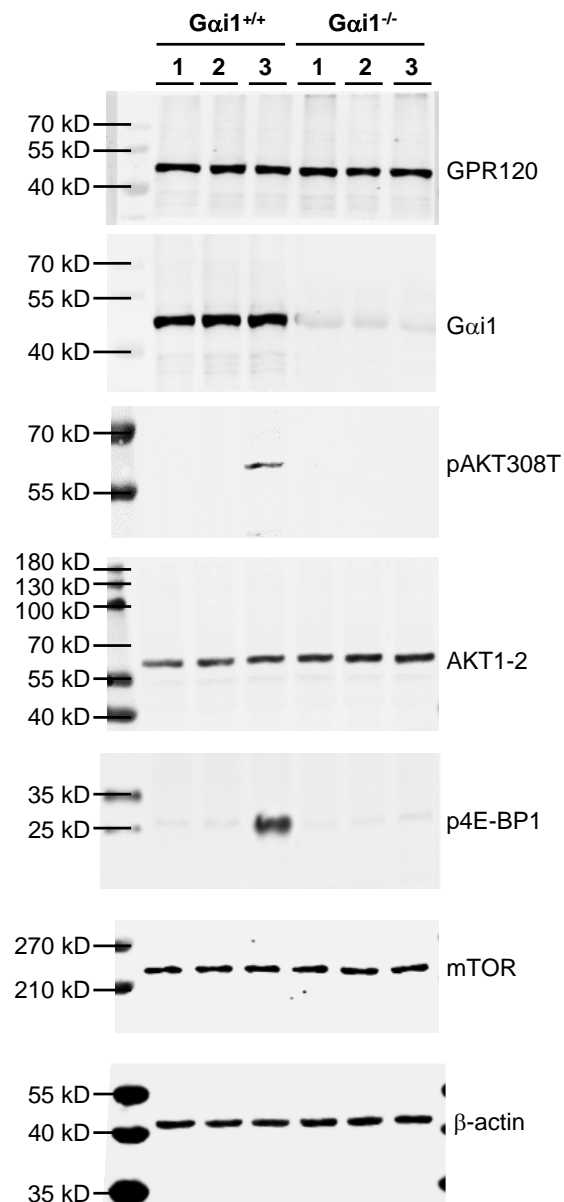

Extended Data Fig. 7c:

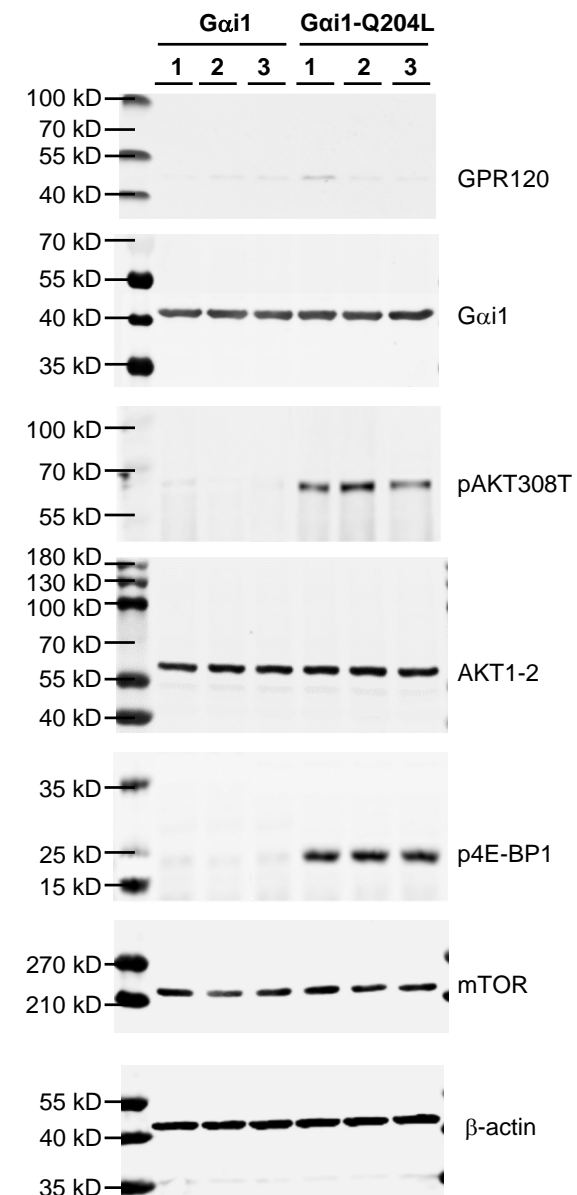

Supplement: Supplementary file 19 — Unprocessed western blots. [file 43587_2025_1028_MOESM19_ESM.pdf]
