# Supplementary material for: Allosteric activation of a cell-type-specific GPR120 inhibits amyloid pathology of Alzheimer’s disease
Source: Nat Aging. 2025 Dec 19;6(1):181–99. doi: 10.1038/s43587-025-01028-4 (PMC12823430; doi:10.1038/s43587-025-01028-4)

Extended Data Fig. 9a

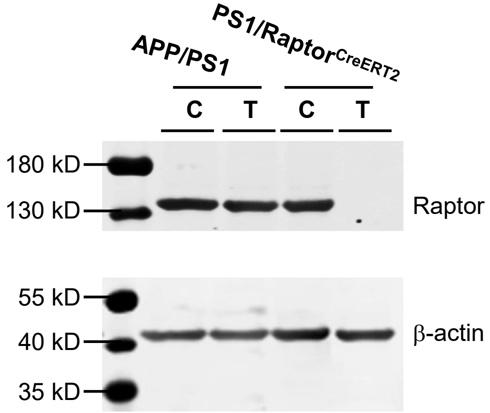

Extended Data Fig. 9b

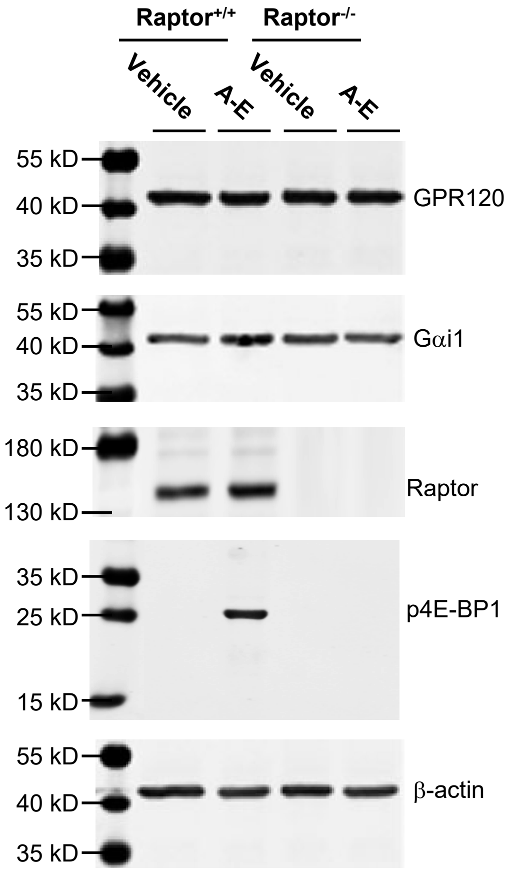

Supplement: Supplementary file 22 — Unprocessed western blots. [file 43587_2025_1028_MOESM22_ESM.pdf]
